# International consensus statement on the diagnosis and management of autosomal dominant polycystic kidney disease in children and young people

**DOI:** 10.1038/s41581-019-0155-2

**Published:** 2019-05-22

**Authors:** Charlotte Gimpel, Carsten Bergmann, Detlef Bockenhauer, Luc Breysem, Melissa A. Cadnapaphornchai, Metin Cetiner, Jan Dudley, Francesco Emma, Martin Konrad, Tess Harris, Peter C. Harris, Jens König, Max C. Liebau, Matko Marlais, Djalila Mekahli, Alison M. Metcalfe, Jun Oh, Ronald D. Perrone, Manish D. Sinha, Andrea Titieni, Roser Torra, Stefanie Weber, Paul J. D. Winyard, Franz Schaefer

**Affiliations:** 1 Division of Pediatric Nephrology, Department of General Pediatrics, Adolescent Medicine and Neonatology, Center for Pediatrics, Medical Center–University of Freiburg, Faculty of Medicine, Freiburg, Germany; 2 Department of Medicine IV, Medical Center–University of Freiburg, Faculty of Medicine, University of Freiburg, Freiburg, Germany; 3 Center for Human Genetics, Bioscientia, Ingelheim, Germany; 4 University College London, Great Ormond Street Hospital, Institute of Child Health, London, UK; 5 Department of Pediatric Radiology, University Hospital of Leuven, Leuven, Belgium; 6 Rocky Mountain Pediatric Kidney Center, Rocky Mountain Hospital for Children at Presbyterian St Luke’s Medical Center, Denver, CO USA; 7 Department of Pediatrics II, University Hospital Essen, Essen, Germany; 8 Renal Department, Bristol Royal Hospital for Children, Bristol, UK; 9 Division of Nephrology and Dialysis, Ospedale Pediatrico Bambino Gesù-IRCCS, Rome, Italy; 10 Department of General Pediatrics, University Children’s Hospital, Münster, Germany; 11 PKD International, Geneva, Switzerland; 12 PKD Charity, London, UK; 13 Division of Nephrology and Hypertension, Mayo Clinic, Rochester, MN USA; 14 Department of Pediatrics and Center for Molecular Medicine Cologne, University of Cologne, Faculty of Medicine and University Hospital Cologne, Cologne, Germany; 15 Department of Pediatric Nephrology, University Hospital of Leuven, Leuven, Belgium; 16 PKD Research Group, Laboratory of Pediatrics, Department of Development and Regeneration, GPURE, KU Leuven, Leuven, Belgium; 17 Faculty of Health and Wellbeing, Sheffield Hallam University, Sheffield, UK; 18 Department of Pediatrics, University Medical Center Hamburg–Eppendorf, Hamburg, Germany; 19 Division of Nephrology, Department of Medicine, Tufts Medical Center, Boston, MA USA; 20 Kings College London, Department of Paediatric Nephrology, Evelina London Children’s Hospital, London, UK; 21 Department of Nephrology, University of Barcelona, Barcelona, Spain; 22 Department of Pediatrics, University of Marburg, Marburg, Germany; 23 Division of Pediatric Nephrology, Center for Pediatrics and Adolescent Medicine, University Hospital, Heidelberg, Germany

## Abstract

These recommendations were systematically developed on behalf of the Network for Early Onset Cystic Kidney Disease (NEOCYST) by an international group of experts in autosomal dominant polycystic kidney disease (ADPKD) from paediatric and adult nephrology, human genetics, paediatric radiology and ethics specialties together with patient representatives. They have been endorsed by the International Pediatric Nephrology Association (IPNA) and the European Society of Paediatric Nephrology (ESPN). For asymptomatic minors at risk of ADPKD, ongoing surveillance (repeated screening for treatable disease manifestations without diagnostic testing) or immediate diagnostic screening are equally valid clinical approaches. Ultrasonography is the current radiological method of choice for screening. Sonographic detection of one or more cysts in an at-risk child is highly suggestive of ADPKD, but a negative scan cannot rule out ADPKD in childhood. Genetic testing is recommended for infants with very-early-onset symptomatic disease and for children with a negative family history and progressive disease. Children with a positive family history and either confirmed or unknown disease status should be monitored for hypertension (preferably by ambulatory blood pressure monitoring) and albuminuria. Currently, vasopressin antagonists should not be offered routinely but off-label use can be considered in selected children. No consensus was reached on the use of statins, but mTOR inhibitors and somatostatin analogues are not recommended. Children with ADPKD should be strongly encouraged to achieve the low dietary salt intake that is recommended for all children.

## Abstract (web summary)

This Consensus Statement developed on behalf of the Network for Early Onset Cystic Kidney Disease provides guidance on counselling, diagnosing and monitoring children with autosomal dominant polycystic kidney disease based on current evidence and a multi-stakeholder discussion of ethical issues.

## Introduction

Autosomal dominant polycystic kidney disease (ADPKD) is the most common genetic disease in adults, with an estimated prevalence of 1 in 500–2,500 (refs^1–4^). Cyst development starts early in life, and macroscopic cysts can become detectable in childhood. Substantial disease burden with massively enlarged kidneys or decreased glomerular filtration rate (GFR) usually does not occur until adulthood^5^; however, approximately 3% of children who carry ADPKD-causing mutations have either very-early-onset or unusually rapid progressive disease^5–7^. Thus, the absolute incidence of symptomatic ADPKD in childhood is thought to be higher than that of other severe paediatric kidney diseases such as autosomal recessive polycystic kidney disease (~1 in 20,000), nephrotic syndrome (~1 in 50,000)^8^ or haemolytic uraemic syndrome (~1 in 100,000 children)^9^.

The past 25 years have seen remarkable progress in knowledge of ADPKD. Advances have been made in unravelling the genetic origins of the disease, in non-invasive monitoring and in predicting disease progression; multiple large-scale clinical trials have been conducted; and the first pharmacological treatment for slowing disease progression — the vasopressin antagonist tolvaptan — has been licensed in the USA, Europe and Japan^10^. However, most ADPKD studies have been performed in adults, and their results are not always easily transferable to children.

Children with ADPKD constitute a mixed cohort of healthy individuals who may not require treatment for decades (referred to here as asymptomatic patients) and individuals who have disease manifestations, such as hypertension, and will benefit from treatment started as early as possible. Few children suffer from symptomatic disease manifestations such as pain, enuresis, haematuria or urinary tract or cyst infections. Both symptomatic and asymptomatic children are likely to be confronted with the effects of ADPKD in older relatives and to have questions or anxieties about their own future health. In addition, many children with an affected parent are unaware of their own disease status (referred to here as at-risk children), either because diagnostic testing has not been performed or because a negative ultrasonography scan does not exclude ADPKD in childhood. An important dilemma in the medical care of children with ADPKD is the balance between not medicalizing currently healthy individuals and not missing treatable disease manifestations in those affected at an early age. Medical professionals from different backgrounds, nurses, affected parents and at-risk children naturally have different views on where this balance lies.

The objective of this Consensus Statement is to provide clinical guidance on counselling, diagnosing and monitoring children with ADPKD in light of the current evidence and a multi-stakeholder discussion of ethical issues surrounding early diagnosis and monitoring.

## Methods

The consensus process was initiated by the Network for Early Onset Cystic Kidney Disease (NEOCYST), which is a consortium of clinical, genetic and translational researchers devoted to the study of early-onset cystic kidney diseases^11^. In addition to paediatric nephrologists from the consortium, external experts in paediatric ADPKD, adult ADPKD, cystic kidney disease genetics, paediatric radiology and patient representatives were invited to participate (Supplementary information). C.G., M.C., R.D.P., R.T., J.K., M.D.S., J.K., A.M.M., A.T. and D.M. prepared systematic literature reviews in advance of the consensus conference held on 1 December 2017 in Leuven, Belgium. Tabulated results of the literature reviews are included in the Supplementary information.

**Fig. 1:**
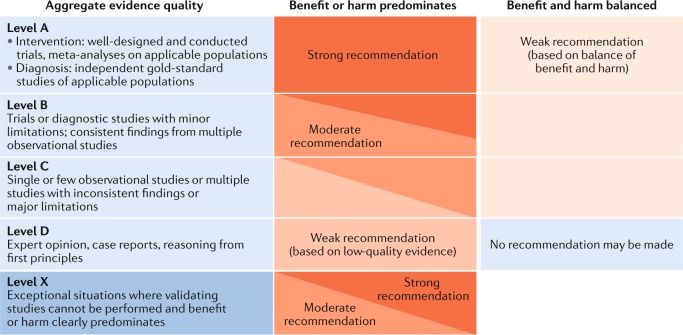
Matrix used for grading of evidence and assigning strength of recommendations. This matrix is currently used by the American Academy of Pediatrics^12,13^. Reproduced with permission from ref.^13^: *Pediatrics* **140**, e20171904 Copyright © 2017 by the AAP.

Initial recommendations were developed during the conference by discussion in thematic workgroups and plenary sessions. Evidence and recommendations were graded according to the method used in the current American Academy of Pediatrics (AAP) guidelines^12,13^ (Fig. 1). The grading of recommendations into strong, moderate and weak recommendations takes into account not only the quality of the evidence but also the balance of potential benefits and harms assessed by the consensus group^12^. The preliminary results of the consensus meeting were presented on 2 December 2017 at an international symposium on ‘Management of Polycystic Kidney Diseases from Childhood to Adulthood’ in Leuven, Belgium, where 104 participants voted live and anonymously on the major drafted recommendations. A first written draft was compiled by C.G. and reviewed by all members of the consensus group. Consequently, two rounds of anonymous voting were performed using the Delphi method until each recommendation reached at least 70% support. The results of the symposium votes and the Delphi votes are presented in the Supplementary information.

The final draft of the Consensus Statement was endorsed by the council of the International Pediatric Nephrology Association (IPNA) and the European Society of Paediatric Nephrology (ESPN) after thorough review by members of the ESPN Workgroup for inherited kidney diseases. The manuscript was also reviewed by ADPKD experts from the European Rare Kidney Disease Reference Network (ERKNet), whose helpful comments were incorporated. Suggestions for further research are listed in the Supplementary information.

## Screening in at-risk minors

The question of whether to screen at-risk children of parents affected by ADPKD is a regularly encountered but often contentious clinical issue^14^. Both genetic testing and ultrasonography screening should be considered diagnostic and require prior counselling (Box 1).

### Counselling

Uncertainty about a child’s disease status causes a high psychological burden for many families^15^. Parents of at-risk children should be informed about the possibilities, limits and consequences of genetic and clinical testing of their children by appropriately skilled personnel. They should understand that screening examinations do not always yield definitive results and importantly that a normal kidney ultrasonography scan has a low negative predictive value in children. Offering diagnostic screening does not imply that this screening is advised but provides parents with the opportunity to make an informed choice. As time for non-directional genetic counselling may be limited in general practice and adult nephrological care, referral to a geneticist or specialized ADPKD clinic may be required. In the case of a child being presented to a paediatric nephrology clinic for testing, adequate parental understanding of the ethical issues should be confirmed before screening. Reliable external information, such as patient support groups, can also help in decision-making.

An important argument against diagnostic testing for ADPKD in childhood is respecting the autonomy of the children or young adults to decide whether to undergo testing for a genetic disease for which a diagnosis might not have therapeutic consequences until adulthood. Treatments to slow disease progression in children with ADPKD are limited, and no clear evidence exists to suggest that presymptomatic detection improves outcomes^16^. In addition, establishing a clinical or genetic diagnosis may have a substantial impact on the future ability of the child to secure insurance policies or to gain access to certain professions. Considerations about insurance vary substantially by place of residence; in some countries, the results of genetic screening tests can legally be kept confidential, whereas in others a positive family history alone will affect insurability.

On the other hand, both the American and the European Society of Human Genetics consider presymptomatic testing of minors for conditions with adult-onset acceptable if preventive actions can be initiated before adulthood^17,18^. Cohort studies in children with ADPKD show an elevated incidence of hypertension, proteinuria and left ventricular hypertrophy, which affect prognosis and are amenable to treatment^19^. Although these data are from tertiary referral centre populations and thus may be biased towards more severe cases, they demonstrate that a subgroup of children exists in whom preventive treatment may be beneficial. Another potential advantage of early diagnosis is that the teenage years can provide a valuable opportunity to integrate lifestyle interventions such as a healthy, low-salt diet and adequate fluid intake into the development of eating habits (discussed further below). The advent of treatment to slow disease progression and of pre-implantation genetic diagnosis have persuaded some clinicians to advocate screening for young adults^20^. For children, the situation is less clear as pharmacological studies in paediatric patients are ongoing and safe treatment options for children with acceptable adverse effects and proven benefits have not yet been established.

### Immediate or delayed screening

In our view, parents and young people may reasonably opt for either immediate diagnostic testing to confirm disease status or regular clinical screening to identify disease manifestations with the option of later diagnostic testing. Regular clinical screening mainly comprises measurement of blood pressure and proteinuria, which should be performed at the same intervals as those recommended for children with proven symptomatic or asymptomatic disease (see below). The feasibility of regular blood pressure monitoring in the community may vary in different settings and should be taken into account.

We recommend that parents receive non-directional counselling about the potential benefits and uncertainties of current diagnostic screening tests. Health-care professionals should inform parents with the aim of shared decision-making and encourage them to keep the best interests of the child in mind. Teenagers and competent younger children should be involved in the decision-making process as much as possible, and ideally a family approach will facilitate discussion to balance the views of the parents and the young person. We also encourage talking to children and adolescents about the possibility of disease transmission early on in an age-appropriate way as this positively influences coping and family interactions^21–23^. If discussion with the child is deferred until the formal age of majority, families may benefit from the help of a genetic counselling service or a similarly trained professional^22^.

Box 1 Screening in at-risk minors **Recommendation 1.1**

All parents of at-risk minors should be counselled about inheritance of autosomal dominant polycystic kidney disease (ADPKD) and the potential benefits and harms of diagnostic screening (evidence level X; recommendation level strong).

**Recommendation 1.2**

Parents of at-risk minors should be offered access to diagnostic screening after counselling (evidence level X; recommendation level moderate).

**Recommendation 1.3**

For asymptomatic minors at risk of ADPKD, repeated screening for treatable, but usually asymptomatic disease manifestations (that is, hypertension and proteinuria) without diagnostic testing or immediate diagnostic screening (by ultrasonography or genetic testing) should be considered equally valid approaches to clinical care. The decision whether to perform diagnostic screening should be shared between parents (or legal guardians) and health-care professionals. Minors should be involved where possible (evidence level X; recommendation level moderate).

**Recommendation 1.4**

If the decision is taken not to perform diagnostic screening in childhood, parents should be made aware of their responsibility to inform their children of disease risk when they reach legal age of majority (evidence level X, recommendation level moderate).

## Radiological diagnosis

### Renal ultrasonography

The current gold standard for radiological diagnosis of ADPKD is renal ultrasonography (Box 2). Ultrasonography is an inexpensive and non-invasive method of examination that is particularly suitable for children because of their smaller body size and the fact that the procedure does not require sedation or ionizing radiation. Diagnostic ultrasonography criteria for ADPKD have been established only for adults^24^; however, in children, there is usually no clinical need to make a genetic diagnosis regardless of the ultrasonography findings or to perform more sensitive diagnosis using MRI (which may require sedation) if the renal ultrasonography scan is normal as cyst burden correlates with the risk of the main complications such as hypertension^25^. In line with clinical practice guidelines for adults^26–28^, we recommend reserving genetic testing to selected situations (see below). We have provided detailed guidance on the prenatal and postnatal imaging of single and multiple kidney cysts and their differential diagnosis in separate statements^29,30^.

#### Diagnostic specificity and sensitivity

In a *PKD1* gene linkage analysis study, the diagnostic specificity of one or more cysts on ultrasonography in at-risk children was 89% in those younger than 5 years of age and 100% in those older than 5 years of age^31^. Failure to confirm cysts on follow-up has been reported rarely with single but not with multiple cysts^32–34^ and may be due, for example, to a mistaken dilated calyx, mistaken prominent medullary pyramid or technical difficulties in obese children. For young adults, established imaging diagnostic criteria require at least three renal cysts on ultrasonography^24,35^ or ten on MRI^36^. However, the studies on which these criteria are based did not include patients younger than 15 years of age. Numerous studies have shown that, owing to the gradual appearance of cysts, children with ADPKD usually have a much lower number of cysts than adults, and young children may not yet have detectable cysts on ultrasonography, especially in families with a mild phenotype^31–34,37–42^. Diagnostic sensitivity is therefore better in older than in younger children and with the use of high-resolution versus lower-resolution ultrasonography. Parents should be counselled that the negative predictive value of a normal ultrasonography scan in childhood is limited and that later appearance of cysts may be due to a milder underlying genetic alteration (for example, *PKD2* or *GANAB* (glucosidase-α neutral AB form) mutations) or variability of the individual clinical course^37^. As cysts develop slowly and children with fewer cysts have later onset of hypertension and proteinuria^25,43–45^, children with a normal ultrasonography scan should not be subjected to frequent repeat scans.

#### Multiple cysts

The incidence of simple cysts in children is very low^46^. Multiple kidney cysts in childhood are therefore highly suggestive of ADPKD or another cystic nephropathy (such as cystic dysplasia or multicystic-dysplastic kidney) and should be investigated. Clinical work-up will include inquiry about (related) symptoms, detailed history and physical examination and may require further investigations of other organ systems. Parental examination may reveal previously undetected ADPKD^47^.

#### Solitary cysts

In children with a positive family history, a solitary cyst is a very likely sign of ADPKD, but in rare cases the cyst may not be confirmed on follow-up^32–34^. In children with a negative family history, ultrasonography of the parents should be performed and, if the results are normal, further work-up or follow-up of the child is needed to exclude the appearance of multiple cysts or the development of a complex cyst.

### MRI

In adults and probably also teenage children, MRI is more sensitive than ultrasonography for detection of kidney cysts in ADPKD^36,43,48^. In neonates and infants, high-resolution ultrasonography can detect even small cysts, but no studies have defined a gold standard for imaging kidney cysts in this age group. As MRI usually requires sedation in children younger than 6 years and is more expensive than ultrasonography imaging, it is not the diagnostic method of choice for paediatric ADPKD.

Box 2 Radiological diagnosis **Recommendation 2.1**

Ultrasonography is the current radiological method of choice to screen for autosomal dominant polycystic kidney disease (ADPKD) in children (evidence level B; recommendation level moderate).

**Recommendation 2.2**

In a child under 15 years with a positive family history of ADPKD, sonographic detection of one or more kidney cysts is highly suggestive of ADPKD (evidence level B, recommendation level moderate). In a fetus or neonate with a positive family history of ADPKD, hyperechogenic and/or enlarged kidneys (>2 s.d.) on ultrasonography are suggestive of ADPKD (evidence level C; recommendation level moderate).

**Recommendation 2.3**

If kidney ultrasonography is normal in an at-risk child, this finding does not exclude ADPKD. However, if making a diagnosis based on ultrasonography is requested, it is not necessary to rescreen at intervals shorter than 3 years (evidence level C; recommendation level moderate).

**Recommendation 2.4**

Multiple cysts with negative family history require clinical work-up for cystic kidney diseases (evidence level B, recommendation level moderate).

**Recommendation 2.5**

Detection of a solitary cyst in childhood requires follow-up imaging (evidence level C; recommendation level moderate).

**Recommendation 2.6**

There are no established MRI-based diagnostic criteria for ADPKD in children younger than 15 years (evidence level B–C; recommendation level moderate).

## Molecular diagnosis

In adults, genetic testing for ADPKD is usually not done because of the clearly established imaging diagnostic criteria and the technical challenges of sequencing *PKD1*. However, knowledge about genotype–phenotype correlations is increasing, as is the need for more accurate estimation of prognosis in view of novel therapies^49^.

Both very-early-onset ADPKD and rapidly progressive disease may be due to unusual genetic constellations, such as biallelic mutations (homozygous, compound heterozygous or digenic) with at least one weak *PKD1* or *PKD2* hypomorphic allele^5,50–52^. Combinations of an ADPKD allele with an allele of another cystic nephropathy such as *TSC2* (for example, as a contiguous gene deletion syndrome (CGS)) may also radically alter the renal disease phenotype^53^. *HNF1B* mutations can mimic ADPKD with important consequences for prognosis, the likelihood of comorbidities (for example, congenital hepatic fibrosis in *PKHD1* or maturity onset diabetes of the young type 5 (MODY5) in *HNF1B*) and the risk of disease in siblings^54^. Where available, simultaneous analysis of a panel of polycystic kidney disease genes is recommended for children with very-early-onset disease independent of their family history or rapidly progressive cystic kidney disease and negative family history (Box 3). An unusually severe clinical course with a positive family history may also be a sign of an unusual genetic constellation, but the likelihood is much lower than in the aforementioned cases. Patients with incidental finding of a single cyst and no extrarenal features should primarily receive clinical work-up and be followed up by imaging. Genetic testing is unlikely to reveal a monogenic cause or change management.

We currently recommend next-generation sequencing panel examination rather than testing single ADPKD genes because of the large phenotypic overlap between different cystic kidney diseases and large genetic heterogeneity^54^. A cystic kidney disease panel should include adequate coverage of *PKD1*, *PKD2*, *PKHD1*, Daz-interacting zinc-finger protein 1-like (*DZIP1L*), *HNF1B* and genes for other ciliopathies such as nephronophthisis (NPHP) and Bardet–Biedl syndrome (BBS).

Box 3 Molecular diagnosis **Recommendation 3.1**

We recommend offering genetic testing for cystic kidney disease genes to infants and children with very-early-onset (VEO) symptomatic disease independent of family history and to those with progressive disease (increasing cyst number or kidney volume) and a negative family history (evidence level B; recommendation level moderate).

**Recommendation 3.2**

In patients with a positive family history and unusually severe clinical course, genetic testing may be beneficial (evidence level D; recommendation level weak).

**Recommendation 3.3**

We do not recommend genetic testing in patients with a single cyst, no extrarenal findings and a negative family history of autosomal dominant polycystic kidney disease (ADPKD) (evidence level B–C; recommendation level moderate).

**Recommendation 3.4**

For genetic testing in children with VEO polycystic kidney disease or unusually progressive disease with a negative family history, we suggest using a multigene panel, including cystic kidney disease genes with a protocol adequately covering *PKD1* rather than testing single ADPKD genes (evidence level C; recommendation level weak).

## Hypertension

Hypertension is one of the most common complications of ADPKD in childhood. A systematic review by Marlais et al. that included >900 children with ADPKD from 14 studies reported that the prevalence of hypertension was 20% (95% CI 15–27%); this prevalence was shown to increase with age in a meta-regression analysis^19^. This finding might be influenced by tertiary centre referral bias because the clinical experience with adult patients suggests that hypertension frequently becomes apparent later in life. The average age of onset of hypertension in adults with ADPKD is 30–34 years^55^ and precedes loss of renal function. A study from large centres that included children and adults confirmed a risk of hypertension of approximately 20% for those younger than 19 years of age, which is significantly higher than that of this age group in the healthy population (<2%)^56^. Elevated blood pressure is significantly associated with kidney volume and cyst score in children with ADPKD^25,43–45,57^.

### Blood pressure measurement

As hypertension is the main treatable disease manifestation of ADPKD in childhood, we recommend adhering to the stringent yearly monitoring intervals for clinic blood pressure measurements recommended by the AAP and the European Society of Hypertension (ESH) for otherwise healthy children^13^ (Box 4). This recommendation also applies to at-risk children of affected parents who have chosen to defer diagnostic testing.

Ambulatory blood pressure monitoring (ABPM) is more reproducible and accurate than clinic blood pressure measurement^58^, and ABPM values associate more closely with left ventricular hypertrophy in children^59^ and with renal disease progression or death in adults with chronic kidney disease (CKD)^60^ than do clinic blood pressure values. A substantial proportion of children with CKD have masked hypertension^61^, justifying the routine use of ABPM for high-risk children^13^. In addition, ABPM can exclude white coat hypertension and help to avoid unnecessary treatment^62^. ABPM becomes more useful as children age because measurements are less well tolerated by younger children, who also have a lower prevalence of hypertension^19^, and reliable reference values are available only for individuals with a height of ≥120 cm. Isolated night-time hypertension with normal daytime blood pressure, which can be picked up only on ABPM, has been reported in 16–18% of children with ADPKD^25,57^. This finding underlines the usefulness of ABPM in this patient group. Monitoring intervals will depend on local availability, level of clinic blood pressure and/or the use of antihypertensive medication.

Home blood pressure measurements are less suitable than repeated clinic or ABPMs for initial diagnosis of hypertension owing to the current lack of definitive paediatric reference values^13^. However, home measurements can be useful to assess changes over time, monitor treatment and increase compliance, thus helping to reduce the frequency of more costly ABPM^63,64^. The use of home blood pressure measurement will depend on the compliance of the family and the child, as well as the availability of monitors that have been specifically validated for children.

### Antihypertensive treatment

#### Blood pressure thresholds

No studies on blood pressure thresholds for antihypertensive treatment in paediatric hypertension have been published. Owing to the high cardiovascular mortality of patients with childhood-onset CKD^65^ and the beneficial effect of lowering blood pressure on progression of renal disease^66^, we support the low antihypertensive treatment threshold (ninetieth percentile for age, sex and height, which equals 130/85 mmHg on clinic measurements for those ≥16 years of age) that is recommended for children with CKD by Kidney Disease: Improving Global Outcomes (KDIGO)^67^.

#### Blood pressure targets

A randomized controlled trial (RCT) of antihypertensive therapy in children with CKD stage 2–4 showed a significant beneficial effect on renal survival when treatment was targeted to reduce 24-hour mean arterial pressure to below the fiftieth percentile on ABPM^66^. However, a post hoc analysis reported no further benefit for an achieved blood pressure below the seventy-fifth percentile^66^. The HALT-PKD Study A demonstrated a significant benefit of a lower blood pressure goal (95/60 to 110/75 mmHg) versus a standard blood pressure target (120/70 to 130/80 mmHg) in terms of total kidney volume (TKV), left ventricular mass index (LVMI) and albuminuria in adults with ADPKD and stage 1–2 CKD^68^. However, a smaller randomized trial of intensive blood pressure control in children with ADPKD did not reach statistical significance^69^. Thus, the long-term benefits of lower blood pressure need to be balanced against the need for more medications and higher risk of adverse effects in the short term. We consider the KDIGO and ESH blood pressure target for children with CKD (less than the seventy-fifth percentile) to be more evidence-based for use in children with ADPKD than the stricter AAP target (less than the fiftieth percentile).

#### First-line treatment

Compared with other antihypertensive agents, angiotensin-converting enzyme (ACE) inhibitors and angiotensin receptor blockers (ARBs) have the largest evidence base for efficacy and safety in paediatric patients and in patients with renal hypertension. In patients with proteinuria, the superiority of ACE inhibitors and ARBs over other antihypertensive drug classes has been clearly demonstrated^70^. Whether renin–angiotensin–aldosterone system (RAAS) inhibitors have superior efficacy to β-blockers or calcium channel blockers in adults with ADPKD is less clear^71–74^. Dual RAAS blockade does not seem to have additional benefit on disease progression over that of improved blood pressure control compared with an ACE inhibitor or ARB alone in adults with early or late ADPKD^68,75^. Diuretics should be used with caution as they may increase vasopressin levels and seem to have deleterious effects on estimated GFR (eGFR) in comparison to ACE inhibitors in ADPKD^76^. In an animal model of ADPKD, calcium channel blockers promoted cyst growth^77^, but the findings of human studies are inconsistent^73,78,79^.

Box 4 Hypertension **Recommendation 4.1**

All children at risk of or diagnosed with autosomal dominant polycystic kidney disease (ADPKD) should have their blood pressure measured at least once a year (that is, at the same interval as healthy children) (evidence level C; recommendation level moderate).

**Recommendation 4.2**

Twenty-four-hour blood pressure on ambulatory blood pressure monitoring (ABPM) is the preferred method for defining hypertension in children aged 5 years and older (evidence level C; recommendation level moderate).

**Recommendation 4.3**

In children with confirmed ADPKD, ABPM should be performed at least once from age 5 years (evidence level B; recommendation level moderate).

**Recommendation 4.4**

For children with ADPKD on antihypertensive medication, we suggest monitoring blood pressure control by regular home blood pressure measurements (evidence level C; recommendation level weak).

**Recommendation 4.5**

We suggest that children with ADPKD receive antihypertensive treatment if their blood pressure repeatedly exceeds the ninetieth percentile or is >130/85 mmHg if age >16 years (evidence level D; recommendation level moderate).

**Recommendation 4.6**

For treatment of hypertensive children with ADPKD, we suggest a target blood pressure below the seventy-fifth percentile or <125/72 mmHg if age >16 years (evidence level C; recommendation level moderate).

**Recommendation 4.7**

Lowering blood pressure below the fiftieth percentile or <120/70 mmHg if age >16 years may provide additional long-term benefit for hypertensive children with ADPKD (evidence level D; recommendation level weak).

**Recommendation 4.8**

We recommend using angiotensin-converting enzyme (ACE) inhibitors or angiotensin receptor blockers as first-line antihypertensive treatment in children with ADPKD who have hypertension and albuminuria (evidence level B; recommendation level moderate).

**Recommendation 4.9**

We suggest using ACE inhibitors or angiotensin receptor blockers as first-line antihypertensive treatment in children with ADPKD who have hypertension without albuminuria (evidence level C; recommendation level moderate).

## Proteinuria

As mentioned above, the incidence of proteinuria is increased in children with ADPKD. In the systematic review by Marlais et al., the prevalence of proteinuria in these children was 20% (96% CI 9–40%)^19^; however, this finding may also be influenced by tertiary centre bias. Proteinuria does not seem to be associated with hypertension but is one of the most established risk factors for progression of CKD in adults and children regardless of the cause of CKD^80–85^. Owing to its therapeutic and prognostic relevance, monitoring of proteinuria and/or albuminuria should be considered standard care for children with ADPKD (Box 5).

Proteinuria can be considered not only as a marker of CKD but also as a cause of further tubulointerstitial damage and fibrosis, as well as glomerular hypertrophy^86^. Reduction of proteinuria using ACE inhibitors or ARBs is associated with significant improvement in renal survival in patients with CKD^87^, although RCTs in children are lacking. Control of proteinuria is therefore an important aspect of current treatment recommendations for CKD^67^. In children with ADPKD who have proteinuria, ACE inhibitors or ARBs should be used as the primary treatment as in other chronic kidney diseases.

Albuminuria tends to be mild in adults with ADPKD; for example, in the TEMPO 3:4 study, the median albumin/creatinine ratio (ACR) was 3.2 mg/mmol. Of all patients, 49% of patients had moderate albuminuria (ACR ≥3 mg/mmol) and just 3.4% had severe albuminuria (ACR ≥30 mg/mmol)^88^. We therefore recommend measuring ACR in a laboratory rather than performing dipstick testing, which is a less sensitive and specific method.

Box 5 Proteinuria **Recommendation 5.1**

Children with autosomal dominant polycystic kidney disease (ADPKD) and those at risk of ADPKD should be monitored for albuminuria (evidence level C; recommendation level weak).

**Recommendation 5.2**

If proteinuria is present, angiotensin-converting enzyme inhibitors or angiotensin receptor blockers should be used as primary treatment as in other chronic kidney diseases (evidence level C; recommendation level moderate).

## Routine monitoring of cyst growth

In asymptomatic children, routine monitoring of cyst growth should not be performed too frequently as ultrasonography findings are very unlikely to influence clinical management decisions and the psychological burden of regular ‘cyst counting’ should be considered. Cyst number and TKV correlate with hypertension^25,43–45^, but ultrasonography cannot replace direct measurement of blood pressure, which remains essential in clinical practice. Although children with very-early-onset ADPKD have poorer outcomes^89^, no studies have examined whether the prediction of ‘rapid progressors’ by repeated imaging in children is feasible and clinically helpful. Our recommendation (Box 6) would change in the future if paediatric studies show a predictive value of kidney imaging on later progression to end-stage renal disease (ESRD; as has been shown in adults^90^) and a treatment to slow disease progression is licensed specifically for children who are at risk of early progression to ESRD.

As discussed below, ultrasonography examination is an essential tool in the investigation of symptomatic children, for example, when investigating urinary tract infections (UTIs), cyst haemorrhage, gross haematuria, renal stones or cyst infections. Before transition from paediatric to adult care, ultrasonography findings may also provide some guidance as to whether to refer the patient to a general practitioner or directly to a nephrologist.

Box 6 Routine monitoring of cyst growth **Recommendation 6.1**

In asymptomatic children with autosomal dominant polycystic kidney disease (ADPKD), the clinical value of repeated ultrasonography is unclear. Depending on the clinical course and the age of the patient, ultrasonography may provide insights into the dynamics of disease progression but, in routine clinical care of classical ADPKD, we suggest that monitoring intervals shorter than 3 years are unnecessary (evidence level X, recommendation level weak).

## Monitoring progression in ADPKD trials

Patient and renal survival are the most meaningful long-term outcomes in ADPKD trials but are difficult to assess in paediatric populations. GFR is nearly always within the normal range during childhood ADPKD^19,33^, thus eGFR decline is a suitable marker of disease progression only in the small subgroup of children with very advanced ADPKD^89^. Height-adjusted TKV (htTKV) on MRI is the most established imaging surrogate parameter for monitoring disease progression in adult ADPKD trials^91^. To date, only one study has investigated the correlation of MRI measurements with disease severity in children with ADPKD. This study found a correlation of MRI cyst volume and TKV with current hypertension status, as well as a predictive value of cyst volume for the development of hypertension^43^. In contrast to adults, kidneys in children with early ADPKD can usually be imaged within one ultrasonography viewing field (maximum dimension ~17 cm), which enables adequate measurements for volume calculation using the ellipsoid formula^92^. However, quantification of cyst number in older children is probably more accurate with MRI once multiple small cysts become too numerous for counting on ultrasonography. For TKV, MRI measurements seem to be slightly larger than ultrasonography measurements in children with ADPKD, with discrepancies mainly for larger kidneys^43,48^. Correlation of hypertension to kidney volume on renal ultrasonography has been demonstrated in three paediatric studies^25,44,45^. 3D ultrasonography is a promising new tool for TKV measurements in children but requires further validation^48^. As non-cooperative children require sedation for MRI, use of this approach does not seem to be warranted in a research setting without direct benefit for the child (Box 7). Use of MRI planimetry to measure TKV may be appropriate for adolescents in clinical trials or children with very large kidneys.

Box 7 Monitoring progression in ADPKD trials **Recommendation 7.1**

Clinical trials in children with autosomal dominant polycystic kidney disease (ADPKD) should monitor hypertension, proteinuria, kidney volume, cyst volume (or number) and (estimated) glomerular filtration rate (evidence level X; recommendation level moderate).

**Recommendation 7.2**

For kidney volume measurements in clinical trials, total kidney volume determined by MRI is recommended to monitor progression in cooperative children. Ultrasonography monitoring of kidney size and cyst number is preferable to MRI in non-cooperative children (evidence level X; recommendation level moderate).

## Lifestyle interventions and treatments

### Maintenance of normal weight

No RCTs of lifestyle interventions with relevant outcome measures have been conducted in patients with ADPKD. However, there is no evidence to suggest that lifestyle recommendations for the general paediatric population, as well as those for children and adults with CKD, do not apply to children with ADPKD (Box 8). The importance of maintaining normal weight is underlined by the observation that obesity is an independent predictor of faster loss of renal function in adults with early ADPKD^93^.

### Salt intake

The dietary salt intake of the general population of infants, toddlers and older children on a Western diet far exceeds the recommended amounts^94^. In adults with CKD, high salt intake is associated with higher blood pressure, proteinuria and progression to ESRD^95–98^. Higher sodium intake also blunts the antihypertensive and antiproteinuric effects of RAAS blockade^99,100^. In patients with ADPKD, urinary sodium excretion correlates with kidney growth^98,101^. Moreover, in patients with later-stage ADPKD, higher urinary sodium levels (a surrogate for sodium intake) increased the risk of a composite end point of a 50% reduction in eGFR, ESRD or death^98^. Few interventional trials exist, but restricting salt intake lowers blood pressure and proteinuria in adults with ADPKD or CKD^102,103^. In accordance with numerous guidelines for CKD, we recommend that children with ADPKD should aim to achieve the recommended intake for healthy children, which may require extra assistance (for example, advice from a dietician).

### Water and protein intake

High water intake to suppress endogenous vasopressin production is often recommended for patients with ADPKD^26,27^. However, evidence from interventional and observational studies does not confirm a benefit of this intervention^104^, and a randomized trial is still ongoing^105^. Studies suggest that adults with ADPKD are more sensitive to water deprivation than those with IgA nephropathy and produce higher levels of endogenous vasopressin to reach similar levels of urine osmolality to those of healthy individuals^106,107^. Dehydration should therefore be avoided, and patients should be encouraged to drink to satisfy thirst^108^. A low-osmolar diet (low sodium, low protein and adjusted water intake to decrease urinary osmolality to <280 mOsM/kg (280 mmol/kg)) decreased the levels of endogenous copeptin (a surrogate marker of vasopressin) in a short study of adults with ADPKD, but a potential long-term benefit on cyst growth remains speculative^109^. In children with non-ADPKD CKD, an RCT did not find a beneficial effect of a low-protein diet on GFR decline^110^. Unnecessary protein restriction should be avoided in children to reduce the risk of malnutrition.

### Vasopressin analogues

Vasopressin analogues are one of several treatment options for nocturnal enuresis in school-age children^111^. A 1994 study reported a significant increase in urinary frequency and a decrease in urinary concentrating ability in children with severe ADPKD (more than ten cysts)^33^. By contrast, children with ten or fewer cysts had a nonsignificantly increased self-reported urinary frequency and no decrease in concentrating ability compared with children of parents with ADPKD who did not have any cysts on ultrasonography. A study that included 16 children who were diagnosed with ADPKD because of their symptoms found that only 1 of these children presented with enuresis^16^; this frequency is probably similar to that of the general paediatric population. As vasopressin antagonists reduce the rate of cyst growth and eGFR loss in patients with ADPKD^112,113^, vasopressin analogues can reasonably be considered to be detrimental in these patients; therefore, it seems wise to prefer other treatment options for the management of nocturnal enuresis in children with ADPKD^111^.

### Statins

In a prospective, double-blind RCT in 110 children and young adults aged 8–22 years with ADPKD and good renal function, the addition of pravastatin to lisinopril (with target blood pressure in the fiftieth to seventy-fifth percentile) resulted in a significantly slower increase in htTKV than placebo^114^. As expected, eGFR did not differ between the groups. Although LDL and total cholesterol levels did not correlate directly with clinical outcome variables (htTKV, albuminuria and LVMI), the statin-induced change in urinary biomarkers of endothelial dysfunction was associated with prospective change in htTKV^115^. Routine statin treatment for cardiovascular indications is more prevalent in adults than in children. A secondary analysis of the HALT-PKD trials reported no effect of self-reported statin use versus no statin use on TKV or composite end points in adults with ADPKD^116^. Therefore, the encouraging findings in the only controlled paediatric study of statin therapy in ADPKD published to date need to be balanced against the lack of evidence of beneficial effects on renal outcome in the uncontrolled adult study^116^ and the lack of regulatory approval of statins for ADPKD. A controlled trial in adults is ongoing^117^. Paediatric safety data for statins in large cohorts have been published only for children with familial hypercholesteremia^118–121^, and the risk of statin therapy in pregnancy is unclear^122^. We were therefore unable to reach a consensus on the use of statins to slow disease progression in children with ADPKD.

### Vasopressin antagonists

Tolvaptan has been licensed to delay disease progression in adults with ADPKD who are likely to go on to develop ESRD^112,113^ and has also been shown to reduce ADPKD-related pain^123^. Currently, no direct data exist to support the use of vasopressin antagonists in children and adolescents with ADPKD, and no safety studies in this group have been published. However, a multinational, double-blind, placebo-controlled trial of tolvaptan in teenagers with ADPKD is currently underway^124^. Although early initiation of treatment resulting in a longer lifetime treatment period may theoretically lead to a greater absolute prevention of eGFR loss than that achieved with later treatment initiation^125^, the medium-term protection against relative eGFR loss is much lower in patients with preserved renal function than in those with more advanced CKD. Tolvaptan is known to cause occasional hepatic injury in adult ADPKD^126^, but the impact of this agent on liver enzymes in children is not yet known. In addition, treatment with vasopressin antagonists causes substantial polyuria, which is likely to affect sleep and daily activities and thus may influence quality of life. Patients are likely to require additional counselling and support to successfully adhere to such a disruptive treatment during adolescence.

### mTOR inhibitors

Prospective RCTs did not find an eGFR benefit of mTOR inhibitors in adults with ADPKD, and these agents were associated with important adverse effects such as worsening proteinuria, hyperlipidaemia and cytopenias^127–129^. We therefore recommend that mTOR inhibitors should not be used in children and adolescents with classical ADPKD.

In patients with *PKD1*/*TSC2* CGS, mTOR inhibitors are potentially beneficial as renal cysts have been reported to decrease in children with tuberous sclerosis receiving treatment with mTOR inhibitors for other indications^130^. However, as cyst volume does not automatically equate to GFR benefit^127^ and there is no published experience of these drugs in *PKD1*/*TSC2* CGS, they should be reserved for the licensed indications of subependymal giant cell astrocytoma and large angiomyolipomas.

### Somatostatin analogues

Use of somatostatin analogues to delay disease progression in ADPKD has been studied only in adults. RCTs indicate that these agents are beneficial in patients with severe liver disease but do not have a sustained beneficial effect on renal function^131–135^. No severe cases of ADPKD-related liver disease in children have been reported in the literature, and paediatric experience with these drugs is limited. We therefore recommend that somatostatin analogues should not be used in children with ADPKD.

Box 8 Lifestyle interventions and treatments^a^ **Recommendation 8.1**

A healthy lifestyle including physical activity and maintenance of normal weight should be promoted in all patients with autosomal dominant polycystic kidney disease (ADPKD) (evidence level B–C; recommendation level moderate).

**Recommendation 8.2**

Children with ADPKD should be encouraged to achieve the recommended low dietary salt intake (evidence level B; recommendation level moderate).

**Recommendation 8.3**

High water intake and avoidance of excessive protein intake may be beneficial in slowing progression of renal failure in children with ADPKD (evidence level D; recommendation level weak).

**Recommendation 8.4**

Use vasopressin analogues (for example, desmopressin) with caution in children and young people with ADPKD and enuresis due to potential negative effects on cyst growth (evidence level X, recommendation level moderate).

**Recommendation 8.5**

Do not routinely offer vasopressin antagonists to children and young people with ADPKD. Off-label use of vasopressin antagonists can be considered at clinician discretion in children at high risk of early progression based on large total kidney volume, rapid kidney growth, family history, etc. (evidence level D, recommendation level weak).

**Recommendation 8.6**

mTOR inhibitors should not be used in children and adolescents with classical ADPKD (evidence level B, recommendation level moderate).

**Recommendation 8.7**

There is insufficient evidence from adult studies supporting the use of somatostatin analogues in ADPKD. They should not be used in children with ADPKD (evidence level C, recommendation level moderate).

^a^No consensus could be reached on the use of statins to slow disease progression in children with ADPKD.

## Management of complications

### Abdominal pain

Abdominal pain is reported in 10–20% of children with ADPKD^16,33,136^. As abdominal and back pain are very common symptoms among children and adolescents in general, further investigations and treatment need to be guided by acuity, intensity and associated findings^137^ (Box 9). Even in the early stages of ADPKD, episodes of nonspecific abdominal pain are frequently reported by adults and tend to be underestimated by physicians^138^. However, patients might have a biased perception of pain, especially if investigations for abdominal pain led to their incidental diagnosis of ADPKD. Chronic pain requires a multidisciplinary approach to avoid overtreatment or undertreatment and to improve self-management^139^. Chronic and/or high-dose use of nonsteroidal anti-inflammatory agents (NSAIDs) should be avoided because of potential renal adverse effects^140,141^.

### Urinary tract and cyst infections

Cohort studies suggest an increased incidence of UTIs in children with ADPKD (up to 15–25%)^6,16,33,37,136^. These studies may be biased as imaging for UTI may have prompted the diagnosis of ADPKD. As no studies have suggested an increased incidence of complicated or prolonged infections in children with ADPKD, local standards for the diagnosis and treatment of UTI in otherwise healthy children should be applied.

Suspicion of upper UTI in children with ADPKD should lead to examination of urine (and blood) cultures, as well as renal ultrasonography, much the same as in otherwise healthy children^142^. Cyst infection is a very rare complication of childhood ADPKD. If suspected clinically or on ultrasonography imaging, experience in adults suggests that ^18^F-FDG–PET/computed tomography (CT) is superior to contrast CT or MRI to confirm the diagnosis and localize the infected cyst, but this approach can produce false-negative results^143,144^. Treatment of cyst infection has a high failure rate and requires long-term use of antibiotics^145^. Precise localization of an infected cyst can be extremely difficult in patients with many cysts but is necessary only for refractory infection when cyst drainage may be required.

### Haematuria and cyst haemorrhage

Macroscopic haematuria is reported in 5–15% of children with ADPKD^6,16,33,37,45,136,146^. However, studies may overestimate the incidence, as imaging for haematuria may have prompted the diagnosis. Macroscopic haematuria is associated with enlarged TKV in adults^147^ but was not more common in children with severe versus moderate versus no cysts^33^, nor in children with very-early-onset disease versus those with later onset^146^. Gross haematuria before the age of 30–35 years is associated with worse renal survival in adults with ADPKD^148,149^. Observations in adults with severe cyst haemorrhage seem to suggest a benefit of treatment with tranexamic acid^150,151^, but the efficacy of this therapy has not been investigated in children.

### Nephrolithiasis

Nephrolithiasis is an exceedingly rare complication in children with ADPKD, and ultrasonography should be used as the first-line imaging modality to rule out stones or other urinary tract obstructions. If kidney stones are found, additional risk factors for stone disease should be investigated, and a high fluid intake and symptomatic treatment are recommended.

### Liver cysts

As the size and number of ADPKD-related liver cysts are known to increase in pregnancy, avoidance of exogenous oestrogens and hormone replacement therapy is generally recommended for women with ADPKD^152^. However, the prevalence of hepatic cysts in children with ADPKD is <5%, with no reports of severe cases^6,16,33,136,146,153^. The risk of future aggravated liver disease in young women with ADPKD considering hormonal contraceptive therapy should be balanced against the risk of unplanned pregnancy. Assessment of the burden of liver cysts and family history might assist in clinical decision-making.

Box 9 Management of complications **Recommendation 9.1**

Children with autosomal dominant polycystic kidney disease (ADPKD) presenting with abdominal pain should receive normal work-up also considering other causes of pain (evidence level D, recommendation level weak).

**Recommendation 9.2**

Diagnosis and treatment of lower urinary tract infection in children with ADPKD should be the same as in otherwise healthy children (evidence level D, recommendation level weak).

**Recommendation 9.3**

In a child with ADPKD and fever, pyelonephritis and cyst infection should be considered. Kidney ultrasonography is the first imaging modality to investigate the aetiology (evidence level D, recommendation level weak).

**Recommendation 9.4**

In a child with ADPKD and gross haematuria, cyst haemorrhage and nephrolithiasis should be considered. Kidney ultrasonography is the first imaging modality to investigate the aetiology (evidence level D, recommendation level weak).

**Recommendation 9.5**

All young women with ADPKD considering contraceptive therapy should receive counselling on potential aggravation of polycystic liver disease with exogenous oestrogen exposure (evidence level D, recommendation level weak).

## Screening for extrarenal complications

### Mitral valve prolapse

A systematic study published in 1995 reported mitral valve prolapse in 12% of children with ADPKD^154^. This prevalence was significantly higher than that of their healthy siblings (3%) but is within the range reported for healthy children and is lower than that reported for adults with ADPKD (25%)^155–157^. As children without a murmur are unlikely to have haemodynamically relevant mitral valve prolapse, they do not require echocardiographic screening for mitral valve prolapse (Box 10).

### Intracranial aneurysm

In adults with ADPKD, screening for intracranial aneurysms is generally recommended only if additional risk factors are present such as a positive family history, previous intracranial aneurysms or a high-risk profession^26,27,158,159^. However, some authors disagree with this recommendation, arguing that screening is cost-effective^160^. Treatment strategies for unruptured intracranial aneurysms are still controversial. As rupture of intracranial aneurysm is an exceedingly rare complication in childhood^161^, routine screening is not justified. In rare cases with a positive family history and a strong desire to ease anxiety by screening, an individualized approach is justified.

### Liver cysts

No reports exist of clinically relevant complications of ADPKD-related liver cysts in children^6,16,33,37,146,153^. Congenital hepatic fibrosis is not a feature of classical ADPKD. Ultrasonography of the liver may be a reasonable investigation at first presentation of children with suspected ADPKD if alternative diagnoses are being considered or in the case of acute abdominal pain. However, we do not recommend regular screening for liver cysts in children with confirmed ADPKD.

### Referral to specialized centres

Newborn babies and infants with severe cystic disease comprise a heterogeneous group who pose numerous challenges to diagnosis and management. Extended genetic testing of these patients is recommended to inform genetic and prognostic counselling in a specialized centre. However, neonates or fetuses with hyperechogenic kidneys and a family history of ADPKD who do not have symptoms or enlarged kidneys should not be considered to have severe disease. Children with *TSC2*/*PKD1* CGS typically have severe polycystic kidney disease and may reach ESRD in young adulthood^162,163^. We recommend referral to a specialized centre and multidisciplinary care for these patients.

Box 10 Screening for extrarenal complications **Recommendation 10.1**

In children with autosomal dominant polycystic kidney disease (ADPKD) without a heart murmur, screening for mitral valve prolapse is not recommended (evidence level D, recommendation level weak).

**Recommendation 10.2**

Screening for intracranial aneurysms is not recommended for children with ADPKD (evidence level D, recommendation level weak).

**Recommendation 10.3**

Regular screening for liver cysts is not recommended in children with confirmed ADPKD (evidence level D, recommendation level weak).

**Recommendation 10.4**

Early referral to a specialized centre is recommended for the management of children with very-early-onset ADPKD or autosomal recessive polycystic kidney disease-like presentations (evidence level D, recommendation level moderate).

**Recommendation 10.5**

Referral to a specialized centre and multidisciplinary care is recommended for patients with *TSC2*/*PKD1* contiguous gene syndrome (evidence level D, recommendation level weak).

## Psychosocial aspects

The consensus group shares the view of a current multidisciplinary position statement on ADPKD that emphasizes the need for “a holistic and comprehensive assessment of the manifestations, complications, prognosis and impact of the disease (in physical, psychological and social terms) on the patient and their family”^164^. Although young adults with ADPKD usually carry a much lower burden of physical impairment than older patients, they have to make lifestyle, career and family planning choices that will have lifelong effects. Studies in families with ADPKD have shown a high psychological burden of both ‘genetic guilt’ and anxiety about future health problems.

We recommend that families of children with ADPKD should be encouraged to discuss the risk of disease transmission with their children (Box 11). Parents affected by inheritable disease often find communication with their children difficult and desire professional assistance, including developmentally appropriate disease information and advice on managing their children’s emotional reactions^21^. Qualitative studies show that avoiding such discussions can be a source of family tensions through misunderstanding, blame and secrecy^23^, whereas open communication with younger children promotes effective coping strategies and makes families more resilient^23^. Multi-family discussion groups can be a valuable tool for promoting such intrafamilial discussions^165^.

Several lifestyle interventions can be recommended for patients with ADPKD (see above), irrespective of their CKD stage. As teenage and young-adult years provide the unique opportunity to establish healthy living habits without having to break previous habits, this period is an important age for counselling. Relevant messages include the importance of a healthy, low-salt diet, adequate fluid intake, regular physical exercise, avoiding obesity, abstention from smoking and avoidance of nephrotoxic medication. Young women should also be counselled to avoid high-oestrogen-containing contraception products because of potential exacerbation of later liver disease.

Issues of genetic guilt or fear of the future disease course can have a substantial impact on the psychological well-being of young people and families affected by ADPKD. Integrated care should therefore include active inquiry about anxieties and sources of psychological support. Positive messages that can be used to promote a proactive attitude towards disease management are, for example, ‘you are not ill’, ‘you have the opportunity and time to influence later outcome by preventive measures’ or ‘many career choices are open to you’. Young adults may also value discussion about wise disclosure of their renal disease to outsiders. Contact with affected peers via patient communities should be encouraged. Finally, reminding parents of their value as positive role models may be helpful.

Discontinuity of care because of transfer from paediatric to adult nephrology care is an important risk factor for adverse outcome in more severely affected individuals. Local and international guidance on transition should be followed to prevent loss of medical follow-up^166^.

Box 11 Psychosocial aspects **Recommendation 11.1**

Families should be encouraged to openly discuss their disease and future genetic risks with their children, for example, by provision of age-appropriate information and by providing support for family members in managing their own and their children’s emotions (evidence level B, recommendation level moderate).

**Recommendation 11.2**

Care of teenagers with autosomal dominant polycystic kidney disease (ADPKD) should address lifestyle measures, as well as relevant medical issues of prevention (evidence level X, recommendation level moderate).

**Recommendation 11.3**

Care of teenagers with ADPKD should address psychological issues and convey positive messages (evidence level X, recommendation level moderate).

**Recommendation 11.4**

Transition to adult nephrology care should follow best-practice guidelines (evidence level X, recommendation level moderate).

## Conclusions

Although the main burden of disease in ADPKD does not occur until adulthood, a substantial proportion of paediatric and adolescent patients have treatable disease manifestations and a small percentage have symptomatic disease or very-early-onset disease in infancy. This consensus group hopes that our recommendations will improve the care of children with or at risk of ADPKD. We recommend the provision of balanced counselling for families with respect to diagnostic screening, regular monitoring of blood pressure and proteinuria and the avoidance of frequent imaging to monitor cyst growth. We also provide guidance on managing complications, lifestyle interventions and psychosocial aspects. As the pharmacological management of adult ADPKD advances and the results of a paediatric trial of tolvaptan are pending, future challenges will include defining in medical, psychological and economic terms which patients will benefit from early initiation of treatment to delay disease progression.

## Supplementary information

Supplementary Information
