# Supplementary material for: International consensus statement on the diagnosis and management of autosomal dominant polycystic kidney disease in children and young people
Source: Nat Rev Nephrol. 2019 May 22;15(11):713–26. doi: 10.1038/s41581-019-0155-2 (PMC7136168; doi:10.1038/s41581-019-0155-2)
Supplement: Supplementary file 1 — Supplementary Information [file 41581_2019_155_MOESM1_ESM.pdf]

nature REVIEWS NEPHROLOGY

SUPPLEMENTARY INFORMATION
In format as provided by the authors

International consensus statement on the diagnosis and management of autosomal dominant polycystic kidney disease in children and young people

Charlotte Gimpel, Carsten Bergmann, Detlef Bockenhauer, Luc Breysem, Melissa A. Cadnapaphornchai, Metin Cetiner, Jan Dudley, Francesco Emma, Martin Konrad, Tess Harris, Peter C. Harris, Jens König, Max C. Liebau, Matko Marlais, Djalila Mekahli, Alison M. Metcalfe, Jun Oh, Ronald D. Perrone, Manish D. Sinha, Andrea Titieni, Roser Torra, Stefanie Weber, Paul J. D. Winyard and Franz Schaefer

https://doi.org/10.1038/s41581-019-0155-2

International consensus statement on the diagnosis and management of autosomal dominant polycystic kidney disease in children and young people

Supplementary Material

Content

Table S1 consensus group members & working groups **3**

Appendix 1 Suggestions for further research **5**

Appendix 2 Feedback from ADPKD symposium, results of Delphi voting and feedback from academic societies **6**

Background *6*

Considering screening in at-risk minors *7*

Radiological diagnosis of ADPKD *8*

Molecular diagnosis of ADPKD *9*

Hypertension *10*

Proteinuria *11*

Routine monitoring of cyst growth *11*

Monitoring disease progression in clinical trials *12*

Life-style interventions and treatments to slow disease progression *12*

Managing complications *13*

Screening for extrarenal complications *14*

Psychosocial aspects *14*

Appendix 3 Literature reviews **15**

Methodology of literature reviews *15*

Table S2 Reliability of Radiological Diagnosis of ADPKD **16**

Tables S3 Radiological markers of ADPKD progression, with a special focus on children **21**

Table S3a Studies in children correlating radiological markers with disease markers *21*

Table S3b Studies in adults correlating radiological markers with (prospective loss of) renal function *23*

Table S4 Incidence of complications and unusual presentations of ADPKD in children and young people 29

Tables S5 Literature reviews on hypertension in ADPKD 36

Tables S5a Incidence of hypertension in children and adolescents with ADPKD 36

Table S5b Correlation of blood pressure to outcome in children with ADPKD (excluding controlled trials) 38

Table S5c Controlled trials of antihypertensive treatments in ADPKD 40

Tables S6 Literature review on Non-pharmacological treatments to slow the progression of ADPKD 46

Table S6a Water intake and progression of ADPKD 46

Table S6b Salt intake and progression of ADPKD 49

Table S6c Caffeine intake and progression of ADPKD 50

Tables S7 Literature review on vasopressin inhibitors in ADPKD 51

Table S7a Studies assessing the EFFICACY of vasopressin receptor 2 antagonists in reducing symptoms or progression of ADPKD 51

Table S7b Studies on the SAFETY of vasopressin receptor 2 antagonists in ADULTS with ADPKD 55

Table S7c Studies on the SAFETY of vasopressin receptor antagonists IN CHILDREN (for any indication) 57

Tables S8 Literature review on statin therapy in ADPKD, with a special focus on children and adolescents 60

Table S8a Trials of statins in ADPKD 60

Table S8b Studies reporting rates of side-effects of statins in children 63

Table S9 mTOR inhibitors for delaying progression of ADPKD 66

Tables S10 somatostatin analogues for ADPKD 68

Table S10a somatostatin analogues for delaying progression of kidney disease in ADPKD 68

Table S10b somatostatin analogues for delaying progression of liver disease in ADPKD 71

Table S12 Patient perspectives and quality of life in patients with ADPKD 74

Table S1 consensus group members & working groups

Name	Area of expertise	Working group	Affiliation
Bergmann, Carsten	Clinical Genetics, renal genetics	.	Department of Medicine IV, Medical Center – University of Freiburg, Faculty of Medicine, University of Freiburg, Freiburg, Germany. Center for Human Genetics, Bioscientia, Ingelheim, Germany
Bockenhauer, Detlef	Pediatric nephrology, cystic kidney diseases	Complications	University College London, Great Ormond Street Hospital, Institute of Child Health, UK
Breysem, Luc	Pediatric radiology, imaging pediatric ADPKD	Diagnosis and monitoring progression	Department of Pediatric Radiology, University Hospital of Leuven, Belgium.
Cadnapaphornchai, Melissa A.	Pediatric ADPKD, imaging of pediatric ADPKD, hypertension	Hypertension and treatments,	Rocky Mountain Pediatric Kidney Center, Rocky Mountain Hospital for Children at Presbyterian St. Luke's Medical Center, Denver, Colorado, USA
Cetiner, Metin	Pediatric nephrology, renal ultrasound, Bardet-Biedl Syndrome	Diagnosis and monitoring progression	Department of Pediatrics II, University Hospital Essen, Essen, Germany
Dudley, Jan	Guideline development, ADPKD	Diagnosis and monitoring progression	Renal Department, Bristol Royal Hospital for Children, Bristol, UK
Emma, Francesco	Pediatric nephrology	Hypertension and treatments,	Division of Nephrology and Dialysis, Ospedale Pediatrico Bambino Gesù—IRCCS, Rome, Italy
Fritz, Gabriele	Patient representative	Ethics & psychosocial issues	PKD Familiaere Zystennieren e.V. (PKD Cure, Germany)
Gimpel, Charlotte	Pediatric nephrology, guideline development	Ethics & psychosocial issues	Department of General Pediatrics, Adolescent Medicine and Neonatology, Center for Pediatrics, Medical Center - University of Freiburg, Germany
Konrad, Martin	Pediatric nephrology, cystic kidney diseases	Complications	Department of General Pediatrics, University Children's Hospital, Münster, Germany
Korst, Uwe	Patient representative	.	PKD International and PKD Familiäre Zystennieren e.V. (PKD Germany)
Harris, Tess	Patient representative	Ethics & psychosocial issues	PKD international and PKD Charity UK
Harris, Peter C.	Genetics of ADPKD	Diagnosis and monitoring progression	Division of Nephrology and Hypertension, Mayo Clinic, Rochester, Minnesota, USA
König, Jens	Pediatric nephrology, nephronophthisis	Complications	Department of General Pediatrics, University Children's Hospital, Münster, Germany
Liebau, Max C.	Pediatric nephrology, polycystic kidney disease	Diagnosis and monitoring progression	Department of Pediatrics and Center for Molecular Medicine Cologne, University of Cologne, Faculty of Medicine and University Hospital Cologne, Cologne, Germany

Marlais, Matko	Pediatric nephrology, Pediatric ADPKD	Literature reviews	University College London, Great Ormond Street Hospital, Institute of Child Health, UK
Mekahli, Djalila	Pediatric nephrology, polycystic kidney disease	Ethics & psychosocial issues	Department of Pediatric Nephrology, University Hospital of Leuven, Belgium; PKD Research Group, Laboratory of Pediatrics, Department of Development and Regeneration, GPURE, KU Leuven, Belgium
Metcalfe, Alison M.	Psychosocial aspects of adult-onset inherited diseases	Ethics & psychosocial issues	Faculty of Health and Wellbeing, Sheffield Hallam University, UK
Oh, Jun	Pediatric nephrology	Complications	Department of Pediatrics, University Medical Center Hamburg-Eppendorf, Hamburg, Germany
Perrone, Ronald D.	ADPKD in adults, imaging in ADPKD, hypertension and tolvaptan trials in ADPKD, psychosocial aspects	Hypertension and treatments, Ethics & psychosocial issues	Division of Nephrology, Department of Medicine, Tufts Medical Center, Boston, Massachusetts, USA
Schaefer, Franz	Pediatric nephrology, inherited kidney diseases, guideline development	Hypertension and treatments,	Division of Pediatric Nephrology, Center for Pediatrics and Adolescent Medicine, Heidelberg University Hospital, Heidelberg, Germany
Sinha, Manish D.	Pediatric ADPKD, pediatric hypertension	Hypertension and treatments,	Kings College London, Department of Paediatric Nephrology, Evelina London Children's Hospital, London, UK
Titieni, Andrea	Pediatric nephrology	Hypertension and treatments,	Department of General Pediatrics, University Children's Hospital, Münster, Germany
Torra, Roser	Adult nephrology, ADPKD, TSC	Complications	Department of Nephrology, University of Barcelona, Spain
Weber, Stefanie	Pediatric nephrology, renal genetics	Diagnosis and monitoring progression	Department of Pediatrics, University of Marburg, Germany
Winyard, Paul J.D.	Pediatric nephrology, prenatal cystic kidney disease	Ethics & psychosocial issues	University College London, Great Ormond Street Hospital, Institute of Child Health, London, UK

Appendix 1 Suggestions for further research

- Unbiased studies on incidence of hypertension and other complications in children and young people with ADPKD (without tertiary center bias)
- Unbiased studies on development of cysts with age in children with ADPKD in order to develop diagnostic guidelines for children and young people to complement the unified criteria.
- Standardized collection of data on longitudinal course of total kidney volume in children with ADPKD
- Evidence-based definition of fast progressors/ severe cases of pediatric ADPKD.
- Studies on the effect of antihypertensive / antiproteinuric treatment with angiotensin converting enzyme inhibitors on disease progression
- Effect of lifestyle modifications on long-term disease progression (in children)
- Role of genetic modifying factors in early onset ADPKD

Appendix 2 Feedback from ADPKD symposium, results of Delphi voting and feedback from academic societies

Background

On the day after the consensus meeting, preliminary recommendations were presented to participants of a symposium on ADPKD in childhood. Participants were invited to provide immediate, anonymous feedback via an online voting systems. 104 participants provided feedback on at least one statement. Of these, 37% were pediatric nephrologists, 25% adult nephrologists, 23% from other medical professions and 4% were lay persons (e.g. patients, carers), while 12% did not disclose their role. In terms of how many people with ADPKD they know or treat, about equal proportions indicated ≤ 10 (25%), 11-50 (26%), or > 50 (19%), but 30% did not indicate how many patients with ADPKD they knew. In terms of country of origin, 17% came from Belgium, 10% from France, 5% from Germany, 5% from the UK, 5% from North America, 8% from Southern Europe (Italy, Portugal, Spain), 2% from Eastern Europe (Poland & Lithuania), 3% from Turkey, 5% from elsewhere and 41% did not answer on their country of origin.

Several, but not all members of the consensus committee were present at the symposium, where results were displayed for a short period immediately after voting. No structured feedback or written summary of the voting results were provided to the consensus committee.

After the consensus meeting and the symposium, a draft of the recommendations and explanations was put online which all committee members could comment on and discuss interactively. When all members had reviewed the document and issues raised had been resolved, 2 rounds of Delphi voting were performed. 21 members took part in the 1st round and 20 in the 2nd round. An agreement rate or 70% was set to pass recommendations as consensus-based. This resulted in one statement not reaching consensus, while all others passed.

The final draft was then sent to the International Pediatric Nephrology Association (IPNA), the European Society of Pediatric Nephrology (ESPN, working group on inherited renal disorders), the European Renal Association - European Dialysis and Transplant Association (ERA/EDTA, working group on inherited kidney disorders (WGIKD), and the European Rare Kidney Disease Reference Network (ERKNet, workgroup on autosomal dominant structural disorders) where members of the relevant working groups that were not part of the consensus group reviewed the manuscript. Where formal voting was required by the society, members of the consensus group who were also part of the working group abstained from voting. A number of smaller amendements were made to the text as stipulated by the academic societies and presented to the consensus group before submission.

Considering screening in at-risk minors

Rec 1.1: All parents of at-risk minors should be counselled about inheritance of ADPKD, potential benefits and harms of diagnostic screening.

Delphi 1st round: 100% agree

Rec 1.2: Parents of at-risk minors should be offered access to screening after counselling.

Delphi 1st round: 86% agree, 14% neither agree nor disagree

Feedback from academic societies indicated that the wording was ambiguous regarding the meaning of screening (diagnostic or for complications). Therefore "diagnostic" was added for make the sentence unambiguous even out of context. As the intended meaning had been clear to the consensus group members, there was no further vote.

Final wording: **Parents of at-risk minors should be offered access to diagnostic screening after counselling.**

Rec 1.3:

Preliminary wording: **For children at risk of ADPKD, we propose that repeated screening for disease manifestations (mainly hypertension and proteinuria) is an EQUALLY VALID alternative to diagnostic screening.**

Symposium feedback:

Of 95 voters: 24% strongly agree, 42% agree, 24% disagree, 9% strongly disagree.

Voting results by background:

	Strongly agree	Agree	Disagree	Strongly disagree	Total
**Adult Nephrologist**	6<br>26%	9<br>39%	5<br>22%	3<br>13%	23
**Other medical**	7<br>33%	7<br>33%	5<br>24%	2<br>10%	21
**Patient, carer or lay person**	0<br>0%	2<br>50%	2<br>50%	0<br>0%	4
**Pediatric Nephrologist**	8<br>22%	17<br>46%	10<br>27%	2<br>5%	37
**Not provided**	2<br>17%	5<br>42%	1<br>8%	2<br>17%	10
**Total**	23<br>24%	40<br>42%	23<br>24%	9<br>9%	95

1st consensus wording: Repeated screening for treatable disease manifestations and immediate diagnostic screening should be considered equally valid approaches to the care of asymptomatic minors at-risk of ADPKD.

Delphi 1st round: 67% agree, 14% neither agree nor disagree, 19% disagree

2nd consensus wording: For asymptomatic minors at-risk of ADPKD, repeated screening for treatable, but usually asymptomatic disease manifestations (i.e. hypertension and proteinuria) without diagnostic testing and immediate diagnostic screening (by ultrasound or genetic testing) should be considered equally valid approaches to clinical care and the decision whether to perform diagnostic screening should be shared between parents (or legal guardians) and health-care professionals. Minors should be involved where possible.

Delphi 2nd round: 82% agree, 6% neither agree nor disagree, 12% disagree

Comment suggesting to change "and" to "or" in presenting the two alternatives and separating the two sentences for ease of understanding were integrated without another vote.

Final wording: For asymptomatic minors at-risk of ADPKD, repeated screening for treatable, but usually asymptomatic disease manifestations (i.e. hypertension and proteinuria) without diagnostic testing or immediate diagnostic screening (by ultrasound or genetic testing) should be considered

equally valid approaches to clinical care. The decision whether to perform diagnostic screening should be shared between parents (or legal guardians) and health-care professionals. Minors should be involved where possible.

Rec 1.X: The decision to perform diagnostic screening in at-risk minors should be shared between parents (or legal guardians) and health-care professionals. Minors should be involved where possible.

Delphi 1st round: 95% agree, 5% neither agree nor disagree

This recommendation was integrated into recommendation 1.3 to stress the important link between opting for or forgoing immediate diagnostic testing and the informed view of parents and minors themselves.

Rec 1.4: If the decision is taken not to perform diagnostic screening in childhood, parents should be made aware of their responsibility to inform their children of disease risk when they reach legal majority.

Delphi 1st round: 90% agree, 5% neither agree nor disagree, 5% disagree

Radiological diagnosis of ADPKD

Rec 2.1: Ultrasound is the current radiological method of choice to screen for ADPKD in children.

Delphi 1st round: 95% agree, 5% neither agree nor disagree

Rec 2.2:

Preliminary wording: **In a child under 15 years with a positive family history of ADPKD, sonographic detection of one or more kidney cysts is highly suggestive of ADPKD.**

Symposium feedback: 39% strongly agree, 49% agree, 11% disagree, 1% strongly disagree.

1st consensus wording: **Sonographic detection of one or more kidney cysts and/or kidney enlargement (>2 SD) in the presence of positive family history is highly suggestive of ADPKD in children younger than 15 years.**

Delphi 1st round: 90.5% agree, 9.5% neither agree nor disagree

Final wording: **Rec 2.2a In a child under 15 years with a positive family history of ADPKD, sonographic detection of one or more kidney cysts is highly suggestive of ADPKD.**
2.2b Additionally, in a fetus or neonate with a positive family history of ADPKD, hyperechogenic and/or enlarged kidneys (>2 SD) on ultrasound are suggestive of ADPKD.

Rec 2.3:

This recommendation was not included in the draft version. The Delphi question was:
Should we include a novel recommendation on when to re-screen a child at-risk with a negative initial ultrasound?

Delphi 1st round:

48% - If diagnostic screening is requested, and initial US is negative, rescreen later, but not earlier than 3-5 years afterwards
24% - If diagnostic screening is requested, and initial US is negative, rescreen after the age of 18 to be sure
19% - If diagnostic screening is requested, and initial US is negative, rescreen after the age of 15 to be sure
10% - I don't have an opinion on this
0% - No, I'm against making a statement on this
0% - No need to rescreen, I would be confident enough that the child is not affected.

Final wording: **If kidney ultrasound is normal in an at-risk child, this does not exclude ADPKD. However, it is not necessary to rescreen at intervals shorter than 3 years.**

Delphi 2nd round: 90% agree, 10% neither agree nor disagree

Rec 2.4: Multiple cysts with negative family history require clinical work-up for cystic kidney diseases.

Delphi 1st round: 100% agree

Rec 2.5:

Preliminary wording: **Children with a solitary renal cyst should have a follow-up ultrasound, independent of family history.**

Symposium feedback: 22% strongly agree, 57% agree, 17% disagree, 4% strongly disagree

1st consensus wording: **Detection of a solitary cyst in childhood requires follow-up imaging.**

Delphi 1st round: 76% agree, 19% neither agree nor disagree, 5% disagree

Rec 2.6: There are no established MRI-based diagnostic criteria for ADPKD in childhood below 15 years.

Delphi 1st round: 76% agree, 19% neither agree nor disagree, 5% disagree

“in childhood below 15 years” changed to “in children younger than 15 years” at request of editor.

Molecular diagnosis of ADPKD

Rec 3.1: We recommend offering genetic testing for cystic kidney disease genes to infants and children with very-early onset (VEO) symptomatic disease independent of family history and to those with progressive disease (increasing cyst number or kidney volume) and a negative family history.

Delphi 1st round: 95% agree, 5% disagree

Rec 3.2: In patients with a positive family history and unusually severe clinical course, genetic testing may be beneficial.

Delphi 1st round: 90.5% agree, 9.5% neither agree nor disagree

Rec 3.3:

Preliminary wording: **Genetic testing is not recommended in children with a single cyst and a negative family history of ADPKD.**

Symposium feedback: 49% strongly agree, 42% agree, 7% disagree, 2% strongly disagree.

Final wording: **We do not recommend genetic testing in patients with a single cyst, no extrarenal findings and a negative family history for ADPKD.**

Delphi 1st round: 95% agree, 5% neither agree nor disagree

Rec 3.4: For genetic testing, we recommend using a NGS panel including cystic kidney disease genes with a protocol adequately covering *PKD1* rather than testing single ADPKD genes.

Delphi 1st round: 90.5% agree, 9.5% neither agree nor disagree

Feedback from the academic societies led to rephrasing “we recommend” into “we suggest” as recommendation level is weak. A further discussion with another reviewer, the clarification of which patient group is addressed here was added (which was clear only from the context before).

Final wording: **For genetic testing in children with very early onset polycystic kidney disease or unusually progressive disease with a negative family history, we suggest using a NGS panel including cystic kidney disease genes with a protocol adequately covering *PKD1* rather than testing single ADPKD genes.**

Hypertension

Rec 4.1:

Question to symposium members: **In children with confirmed ADPKD, routine clinical examination (blood pressure, proteinuria) should be done every …**

Symposium feedback:
76% - 12 months
20% - 2 years
1% - 5 years
0% - 10 years
3% - not at all

Final wording: **All children at-risk or diagnosed with ADPKD should have blood pressure measured at least once a year (i.e. at the same interval as healthy children).**

Delphi 1st round: 95% agree, 5% disagree

Rec 4.2: 24-hour blood pressure on ABPM is the preferred method for defining hypertension in children aged 5 years and older.

Delphi 1st round: 86% agree, 9% neither agree nor disagree, 5% disagree

Rec 4.3:

Preliminary wording: **All children diagnosed with ADPKD should undergo at least one ambulatory 24-hour blood pressure measurement.**

Symposium feedback: 39% strongly agree, 34% agree, 23% disagree, 4% strongly disagree.

Final wording: **In children with confirmed ADPKD, ABPM should be performed at least once from age 5 years.**

Delphi 1st round: 71% agree, 24% neither agree nor disagree, 5% disagree

Rec 4.4: For children with ADPKD on antihypertensive medication, we suggest monitoring blood pressure control by regular home blood pressure measurements.

Delphi 1st round: 86% agree, 9% neither agree nor disagree, 5% disagree

Rec 4.5:

Question to symposium members: **Antihypertensive treatment should be initiated if blood pressure exceeds the …**

Symposium feedback:
14% - 50th percentile (120/70 mm Hg in a 16 year old)
36% - 75th percentile (125/72 mm Hg in a 16 year old)
42% - 90th percentile (130/85 mm Hg in a 16 year old)
9% - 95th percentile (140/90 mm Hg in a 16 year old)

Final wording: **We suggest that children with ADPKD receive antihypertensive treatment if their blood pressure repeatedly exceeds the 90th percentile (or > 130/85 mm Hg if age > 16 years).**

Delphi 1st round: 90.5% agree, 9.5% neither agree nor disagree

Rec 4.6: For treatment of hypertensive children with ADPKD, we suggest a target blood pressure below the 75th percentile (or < 125/72 mm Hg if age > 16 years).

Delphi 1st round: 86% agree, 9% neither agree nor disagree, 5% disagree

Rec 4.7: Lowering BP below the 50th percentile (or < 120/70 mm Hg if age > 16 years) may provide additional long-term benefit for hypertensive children with ADPKD.

Delphi 1st round: 76% agree, 24% neither agree nor disagree

Rec 4.8: We recommend using ACE inhibitors or angiotensin receptor blockers inhibitor as first-line antihypertensive treatment in of children with ADPKD who have hypertension and albuminuria.

Delphi 1st round: 90.5% agree, 9.5% neither agree nor disagree

Rec 4.9: We suggest using ACE inhibitors or angiotensin receptor blockers as first-line antihypertensive treatment in of children with ADPKD who have hypertension without albuminuria.

Delphi 1st round: 95% agree, 5% neither agree nor disagree

Proteinuria

Rec 5.1:

1st consensus wording: **Children with ADPKD and those at-risk of ADPKD should be monitored for proteinuria.**

Delphi 1st round: 81% agree, 14% neither agree nor disagree, 5% disagree

Would you prefer the wording "microalbuminuria and proteinuria"?

29% agree, 29% neither agree nor disagree, 43% disagree. (Written comments related mainly to the fact that "albuminuria" was preferable to "microalbuminuria")

Do you want to recommend a suggested monitoring interval for proteinuria in at-risk and confirmed asymptomatic cases?

33% – No, I would prefer to leave this an intended ambiguity
14% – yearly
10% – every 2 years
24% – every 3 years
10% – every 5 years
5% – in intervals > 5 years
5% – I don't have an opinion on this

Final wording: **Children with ADPKD and those at-risk of ADPKD should be monitored for albuminuria.**

Rec 5.2: If proteinuria is present, ACE inhibitors or angiotensin receptor blockers should be used as primary treatment as in other chronic kidney diseases.

Delphi 1st round: 95% agree, 5% neither agree nor disagree

Routine monitoring of cyst growth

Rec 6.1:

Question to symposium members: **In children with confirmed ADPKD, routine renal ultrasound should be performed every …**

Symposium feedback:

17% – 12 months
48% – 2 years
26% – 5 years
2% – 10 years
6% – not at all

1st consensus wording: Depending on the clinical course and the age of the patient ultrasound may provide insights into the dynamics of disease progression, but the clinical significance of ultrasound findings in asymptomatic children with ADPKD is unclear. A suggested interval of routine ultrasound monitoring of asymptomatic children is 3-5 years.

Delphi 1st round: 67% agree, 24% neither agree nor disagree, 9% disagree

My personal favorite for a monitoring interval is …

9.5% – no routine imaging in asymptomatic children

0% – yearly
9.5% – 2 years
52% – 3 to 5 years
5% – 5 years
19% – 5 to 10 years
5% – I don't have an opinion on this

2nd consensus wording: In asymptomatic children with ADPKD the clinical significance of ultrasound findings is unclear. Depending on the clinical course and the age of the patient, ultrasound may provide insights into the dynamics of disease progression, but in routine care monitoring intervals < 3 years are unnecessary.

Delphi 2nd round: 75% agree, 20% neither agree nor disagree, 5% disagree

(Written comments stipulated changing "ultrasound findings" to "repeated ultrasound findings" and "routine care" to "routine clinical care of classical ADPKD".)

Final consensus wording: **In asymptomatic children with ADPKD the clinical value of repeated ultrasounds is unclear. Depending on the clinical course and the age of the patient, ultrasound may provide insights into the dynamics of disease progression, but in routine clinical care of classical ADPKD monitoring intervals shorter than 3 years are unnecessary.**

Feeback from academic societies suggested that, as the evidence level and recommendation is low/weak, the wording should include "suggestion".

Final wording: **In asymptomatic children with ADPKD the clinical value of repeated ultrasounds is unclear. Depending on the clinical course and the age of the patient, ultrasound may provide insights into the dynamics of disease progression, but in routine clinical care of classical ADPKD we suggest that monitoring intervals shorter than 3 years are unnecessary.**

Monitoring disease progression in clinical trials

Rec 7.1: Clinical trials in children with ADPKD should monitor hypertension, proteinuria, kidney volume, cyst volume (or number) and (estimated) glomerular filtration rate.

Delphi 1st round: 100% agree

Rec 7.2: For kidney volume measurements in clinical trials, TKV determined by MRI is recommended to monitor progression in cooperative children. US monitoring of kidney size and cyst number is preferable to MRI in non-cooperative children.

Delphi 1st round: 81% agree, 14% neither agree nor disagree, 5% disagree

Life-style interventions and treatments to slow disease progression

Rec 8.1: A healthy lifestyle including physical activity and maintenance of normal weight should be promoted in all ADPKD patients.

Delphi 1st round: 100% agree

Rec 8.2: Children with ADPKD should be especially encouraged to achieve the recommended low dietary salt intake.

Delphi 1st round: 90.5% agree, 9.5 neither agree nor disagree

Rec 8.3: High water intake and avoidance of excessive protein intake may be beneficial in slowing progression of renal failure.

Delphi 1st round: 76% agree, 24% neither agree nor disagree

Rec 8.4: Use vasopressin analogues (e.g. desmopressin) with caution in children and young people with enuresis due to potential effects on cyst growth.

Delphi 1st round: 76% agree, 19% neither agree nor disagree, 5% disagree

Rec 8.X:
Preliminary wording: **Statin therapy can be considered to slow disease progression in children with ADPKD 8 years and older.**

Symposium feedback: 1% strongly agree, 23% agree, 68% disagree, 7% strongly disagree.

1st consensus wording: **Consider offering statin therapy in children with ADPKD aged 8 years and older.**

Delphi 1st round: 43% agree, 33% neither agree nor disagree, 24% disagree

2nd consensus wording: It is permissible to offer statin therapy in children with ADPKD aged 8 years and older.

Delphi 2nd round: 60% agree, 25% neither agree nor disagree, 15% disagree

Final wording: **No consensus could be reached on the use of statins to slow disease progression in children with ADPKD.**

Rec 8.5:
Preliminary wording: **Currently, there is insufficient data supporting the use of vasopressin antagonists in children and adolescentes with ADPKD**

Symposium feedback: 30% strongly agree, 60% agree, 9% disagree, 1% strongly disagree.

Off-label use of vasopressin antagonists can be considered in children with ADPKD at high risk of early progression (based e.g. on large TKV, rapid kidney growth, family history, loss of eGFR)

Symposium feedback: 14% strongly agree, 59% agree, 21% disagree, 6% strongly disagree.

Final wording: **Do not routinely offer vasopressin antagonists to children and young people with ADPKD. Off-label use of vasopressin antagonists can be considered at clinician discretion in children at high risk of early progression based on large TKV, rapid kidney growth, family history etc.**

Delphi 1st round: 81% agree, 14% neither agree nor disagree, 5% disagree

Rec 8.6: mTOR inhibitors should not be used in children and adolescents with classical ADPKD.

Delphi 1st round: 76% agree, 19% neither agree nor disagree, 5% disagree

Rec 8.7: There is insufficient evidence from adult studies supporting the use of somatostatin analogues in ADPKD. They should not be used in children with ADPKD.

Delphi 1st round: 90.5% agree, 9.5% neither agree nor disagree

Managing complications

Rec 9.1: Children with ADPKD presenting with abdominal pain should receive normal work-up also considering other causes of pain.

Delphi 1st round: 90% agree, 5% neither agree nor disagree, 5% disagree

Rec 9.2: Diagnosis and treatment of lower urinary tract infection in ADPKD should be the same as in other children.

Delphi 1st round: 86% agree, 9% neither agree nor disagree, 5% disagree

"in children with" inserted before ADPKD and "otherwise healthy" at request of editor.

Rec 9.3: In a child with ADPKD and fever, pyelonephritis and cyst infection should be considered. Kidney ultrasound is the first imaging modality to investigate the etiology.

Delphi 1st round: 95% agree, 5% neither agree nor disagree

Rec 9.4: In a child with ADPKD and gross hematuria cyst hemorrhage and nephrolithiais should be considered. Kidney ultrasound is the first imaging modality to investigate the etiology.

Delphi 1st round: 95% agree, 5% neither agree nor disagree

Rec 9.5: All young women with ADPKD considering contraceptive therapy should receive counseling on potential aggravation of polycystic liver disease with exogenous estrogen or progesterone exposure.

Delphi 1st round: 95% agree, 5% neither agree nor disagree

Feedback from academic societies suggested a more differentiated approach to estrogens and progesterones. Because this is a minor issue in pediatric practices, progesterone was removed from the text of the recommendation rather than expanding the explanation.

Final wording: **All young women with ADPKD considering contraceptive therapy should receive counseling on potential aggravation of polycystic liver disease with exogenous estrogen exposure.**

Screening for extrarenal complications

Rec 10.1: In children with ADPKD without a heart murmur, screening for mitral valve prolapse is not recommended.

Delphi 1st round: 81% agree, 9.5% neither agree nor disagree, 9.5% disagree

Rec 10.2: Screening for intracranial aneurysms is not recommended for children with ADPKD.

Delphi 1st round: 90.5% agree, 9.5% neither agree nor disagree

Rec 10.3: Regular screening for liver cysts is not recommended in children with confirmed ADPKD.

Delphi 1st round: 86% agree, 9% neither agree nor disagree, 5% disagree

Rec 10.4: Early referral to a specialized center is recommended for the management of children with very early onset ADPKD / ARPKD-like presentations.

Delphi 1st round: 100% agree

Rec 10.5: Referral to a specialized center and multidisciplinary care is recommended for patients with TSC2/PKD1 contiguous gene syndrome.

Delphi 1st round: 95% agree, 5% neither agree nor disagree

Psychosocial aspects

Rec 11.1: Families should be encouraged to openly discuss their disease and future genetic risks with their children, e.g. by provision of age-appropriate information and by providing support for family members in managing their own and their children's emotions.

Delphi 1st round: 100% agree

Rec 11.2: Care of teenagers with ADPKD should address lifestyle measures as well as relevant medical issues of prevention.

Delphi 1st round: 100% agree

Rec 11.3: Care of teenagers with ADPKD should address psychological issues and convey positive messages.

Delphi 1st round: 100% agree

Rec 11.4: Transition to adult nephrology care should follow best practice guidelines.

Delphi 1st round: 100% agree

Appendix 3 Literature reviews

Methodology of literature reviews

MEDLINE and the Cochrane library were systematically searched up to November 2017 with other key references considered later up to August 2018. The searches were restricted to original articles published in English.

A strict Patient-Intervention-Comparison-Outcome format (PICO) proved unrealistic for most questions addressed in this statement, due to the general lack of pediatric controlled trials in ADPKD, but also because many pediatric ADPKD studies address outcomes which are considered subsidiary in adult ADPKD studies. However, a number of literature reviews were still helpful in order to depict our current knowledge about manifestations and severity of pediatric ADPKD (which remain controversial) and knowledge about radiological disease markers during childhood.

Each study was assessed for validity akin to criteria proposed by the Grading of Recommendations Assessment, Development and Evaluation working group (GRADE) (1). This includes ranking each study with respect to risk of bias (limitations in study design or execution), indirectness (how directly the purpose of the study was related to the question of the literature review), inconsistency (whether results were consistent across studies), and imprecision (whether study results were precise enough to draw adequate conclusions) on a scale of “not serious”, “serious” and “very serious”. The categories “serious” or “very serious” indicates that concerns in this domain led to downgrading of the evidence. Reviewers could also list other considerations (e.g. suspicion of publication bias, influence of plausible residual confounding) and rated the study for overall level of importance in answering the health question (on a scale of 1-9, where 9 is most important).

Table S2 Reliability of Radiological Diagnosis of ADPKD

Correlation of radiological markers with genetically confirmed diagnosis of ADPKD, with a special emphasis on children

Title	First Author	Journal	Year	Study design	Nº & type of patients	Methodological details	Prediction of genetic diagnosis	Risk of bias	Indirectness	Inconsistency	Imprecision	Other considerations	Importance (1-9)
**Studies in at-risk children and young adults determining sensitivity and specificity compared to genetic analysis**													
**Ultrasound**													
**PKD 1 only**													
Unified criteria for ultrasonographic diagnosis of ADPKD	Pei, Y.	J Am Soc Nephrol. 20(1):205-12	2009	Diagnostic study in at-risk individuals	113 patients with PKD1 mutations aged 15-29 years + similar number of unaffected siblings (*n* aged <18yr unspecified).	Ultrasound with 3 or 5 MHz sector probe. Simulated data set of 1000 patients bootstrapped from the original dataset	<u>≥1 renal cyst:</u><br>sensitivity 0.991 (0.969 to 1.000)<br>specificity 0.976 (0.945 to 1.000)<br><u>≥2 renal cysts (uni- or bilateral):</u><br>sensitivity 0.981 (0.951 to 1.000)<br>specificity 0.988 (0.963 to 1.000)<br><u>≥3 renal cysts (uni- or bilateral):</u><br>Sensitivity 0.943 (0.891 to 0.981)<br>Specificity 1.000	Serious[1]	Not serious	Not serious	Not serious	Partial overlap with Ravine et al and Parfrey et al	7
Autosomal dominant polycystic kidney disease types 1 and 2: assessment of US sensitivity for diagnosis	Nicolau, C.	Radiology. 213(1):273-6	1999	Diagnostic study in at-risk individuals	146 individuals at risk of PKD1 aged btwn. 9 months-30 years (*n* aged <18yr unspecified).<br>84 affected and 62 unaffected.	Ultrasound with 3.7 or 5 MHz	<u>≥ 2 (uni-or bilateral) renal cyst:</u><br>sensitivity: 0.95<br>Specificity: 1.0<br>False negative results: 4/84 (aged 2,3, 5 and 26 years)<br>False positive results: 0/62	Serious[1]	Not serious	Not serious	Not serious		7
The use of ultrasonography and linkage studies for early diagnosis of autosomal dominant polycystic kidney disease (ADPKD).	Papadopoulou, D.	Ren Fail. 21(1):67-84	1999	Diagnostic study in at-risk individuals	33 children with PKD1 (n=13 < 12 years and n=23 of 12-19 years)	Ultrasound, not further specified.	<u>≥ 2 cysts in one kidney plus ≥ 1 in the other:</u><br>< 12 years: sensitivity 0.231<br>12-19 years: sensitivity 0.869<br>(unaffected children also examined, but as *n* not given, specificity, NPV and PPV analysis not possible)	Not serious	Not serious	Not serious	Not serious	Small sample size. Strict diagnostic criterion.	8
Utility of ultrasonography in the diagnosis of autosomal dominant polycystic kidney disease in children	Gabow, P.A.	Am Soc Nephrol. 8(1):105-10	1997	Diagnostic study in at-risk individuals	105 children with risk of PKD1 mutation.	Ultrasound, not further specified.	<u>≥1 renal cyst</u> (sensitivity, specificity, false negative rate, false positive rate):<br>all children: 0.77, 0.98, 0.25, 0.02<br>3 mths-5 years: 0.62, 0.89, 0.38, 0.11<br>5-10 years: 0.82, 1.0, 0,19, 0.0<br>10-15 years: 0.86, 1.0, 0.23, 0.0<br>15-17.5 years: 0.67, 1.0, 0.22, 0.0	Not serious	Not serious	Not serious	Not serious		8

[1] No separate analysis for pediatric patients

Title	First Author	Journal	Year	Study design	Nº & type of patients	Methodological details	Prediction of genetic diagnosis	Risk of bias	Indirectness	Inconsistency	Imprecision	Other con-siderations	Importance (1-9)
Diagnosis of adult polycystic kidney disease by genetic markers and ultrasonographic imaging in a voluntary family register	Elles, R.G.	J Med Genet. 31(2):115-20.	1994	Diagnostic study in at-risk individuals	56 persons at risk for PKD1 aged < 30 years.	Ultrasound with 3.5 MHz linear or sector probe.	Age group < 30 years:<br>≥ 2 cysts in one kidney plus ≥ 1 in the other:<br>Sensitivity 0.9643<br>Specificity 1.0<br>PPV 1.0, NPV 0.97<br>Age class 0-9 years:<br>Observed frequency of positive ultrasound 23.5 ± 20.1% vs predicted frequency of 49%. I.e. evidence for significant rate of false negatives.<br>Age class 10-19 years:<br>Observed frequency of positive ultrasound 60.4 ± 13.2% vs predicted frequency of 43%. I.e. no evidence for significant rate of false negatives.	Not serious	Not serious	Not serious	Not serious	Separate analysis for 0-9 year olds (but n not given for this group)	8
Evaluation of ultrasonographic diagnostic criteria for autosomal dominant polycystic kidney disease 1	Ravine, D.	Lancet. 2;343(8901):824-7.	1994	Diagnostic study in at-risk individuals	204 previously not examined at-risk individuals of PKD1 (of which 37 were aged 15-19 years)	Ultrasound with 3 or 5 MHz sector probe.	Age 15-29 years ("similar in 15-19 and 20-29yr olds"):<br>≥ 1 renal cyst: sensitivity 0.962<br>≥ 2 (uni-or bilateral) renal cyst: sensitivity 0.962<br>≥ 2 cysts in one kidney plus ≥ 1 in the other:<br>Sensitivity 0.885<br>≥ 2 cysts in each kidney: sensitivity 0.846<br>≥ 4 cysts in each kidney: sensitivity 0.808<br><br>At age 20 years: (PPV and NPV)<br>≥ 1 renal cyst: 1.0, 96.6<br>≥ 2 (uni-or bilateral) renal cyst: 1.0, 96.6<br>≥ 2 cysts in one kidney plus ≥ 1 in the other: 1.0, 90.5<br>≥ 2 cysts in each kidney: 1.0, 87.7<br>≥ 4 cysts in each kidney: 1.0, 85.1	Serious[1]	Not serious	Not serious	Not serious		8
Autosomal dominant polycystic kidney disease: new information for genetic counselling.	Bear, J.C.	Am J Med Genet. 43(3):548-53.	1992	Diagnostic study in at-risk individuals	125 children and young adults at risk of PKD1<br>(n=19 0-9 years, n=52 10-19 years, n=54 20-29 years) (overlap with Parfrey et al).	Ultrasound, not further specified.	≥ 2 cysts in one kidney plus ≥ 1 in the other:<br>Calculated rate of false negative ultrasounds:<br>0-9 years: 36%<br>10-19 years: 8%<br>20-29 years: 0%	Not serious	Not serious	Not serious	Not serious	Separate analysis for 0-9 and 10-19 year olds.<br>Overlap with Parfrey et al.	8
The diagnosis and prognosis of autosomal dominant polycystic kidney disease	Parfrey, P.S.	N Engl J Med. 323(16):1085-90	1990	Diagnostic study in at-risk individuals	Individuals at risk of PKD1 mutation aged < 30 years.<br>48 affected and 23 unaffected	Ultrasound, not further specified.	≥ 2 cysts in one kidney plus ≥ 1 in the other: 40/48 affected and 0/23 unaffected<br>Equivocal: 1/48 affected and 1/23 unaffected<br>No cysts: 7/48 and 22/23 unaffected	Serious[1]	Not serious	Not serious	Not serious		7

Title	First Author	Journal	Year	Study design	Nº & type of patients	Methodological details	Prediction of genetic diagnosis	Risk of bias	Indirectness	Inconsistency	Imprecision	Other considerations	Importance (1-9)
**PKD 2 only**													
Unified criteria for ultrasonographic diagnosis of ADPKD	Pei, Y.	J Am Soc Nephrol. 20(1):205-12	2009	Diagnostic study in at-risk individuals	41 patients with PKD2 mutations aged 15-29 years + similar number of unaffected siblings (*n* aged <18yr unspecified).	Ultrasound with 3 or 5 MHz sector probe. Simulated data set of 1000 patients bootstrapped from the original dataset	≥1 renal cyst:<br>sensitivity 0.791 (0.674 to 0.896)<br>specificity 0.966 (0.911 to 1.000)<br>≥2 renal cysts (uni- or bilateral):<br>Sensitivity 0.719 (0.593 to 0.830)<br>Specificity 1.000<br>≥3 renal cysts (uni- or bilateral):<br>Sensitivity 0.695 (0.567 to 0.812)<br>Specificity 1.000<br>3 truly pediatric patients (aged 16 and 17) are affected by PKD 2 but have NO cysts.	Serious[1]	Not serious	Not serious	Not serious	Partial overlap with Ravine et al and Parfrey et al	7
Autosomal dominant polycystic kidney disease types 1 and 2: assessment of US sensitivity for diagnosis	Nicolau, C.	Radiology. 213(1):273-6.	1999	Diagnostic study in at-risk individuals	15 individuals at risk of PKD2 aged btwn. 9 months-30 years (*n* aged <18yr unspecified).<br>6 affected and 9 unaffected.	Ultrasound with 3.7 or 5 MHz	≥ 2 (uni-or bilateral) renal cyst:<br>sensitivity: 0.67<br>Specificity: 1.0<br>False negative results: 2/16 (aged 5 and 10 years)<br>False positive results: 0/9	Serious[1]	Not serious	Not serious	Not serious		7
**Known familial ADPKD but unknown genotype**													
Unified criteria for ultrasonographic diagnosis of ADPKD	Pei, Y.	J Am Soc Nephrol. 20(1):205-12	2009	Diagnostic study in at-risk individuals	15-29 year olds out of 577 PKD1 and 371 PKD2-at-risk individuals (*n* aged <18yr unspecified).	Ultrasound with 3 or 5 MHz sector probe. Simulated data set of 1000 patients bootstrapped from the original dataset. Case-mix: 85% PKD1, 15% PKD2	≥1 renal cyst:<br>sensitivity 0.893 (0.843 to 0.940)<br>specificity 0.971 (0.943 to 0.994)<br>≥2 renal cysts (uni- or bilateral):<br>Sensitivity 0.848 (0.788 to 0.906)<br>Specificity 0.994 (0.979 to 1.000)<br>≥3 renal cysts (uni- or bilateral):<br>Sensitivity 0.817 (0.750 to 0.877)<br>Specificity 1.000	Serious[1]	Not serious	Not serious	Not serious	Partial overlap with Ravine et al and Parfrey et al	7
Autosomal dominant polycystic kidney disease types 1 and 2: assessment of US sensitivity for diagnosis	Nicolau, C.	Radiology. 213(1):273-6.	1999	Diagnostic study in at-risk individuals	161 individuals at risk of PKD1 (91%) of PKD2 (9%) aged btwn. 9 months - 30 years (37 aged <16yr).<br>90 affected and 71 unaffected.	Ultrasound with 3.7 or 5 MHz	≥ 2 (uni-or bilateral) renal cyst:<br>sensitivity: 0.93<br>Specificity: 1.0<br>False negative results: 6/90<br>False positive results: 0/71	Serious[1]	Not serious	Not serious	Not serious		7

Title	First Author	Journal	Year	Study design	Nº & type of patients	Methodological details	Prediction of genetic diagnosis	Risk of bias	Indirectness	Inconsistency	Imprecision	Other con-siderations	Importance (1-9)
**High-resolution ultrasound**													
Imaging-based diagnosis of autosomal dominant polycystic kidney disease	Pei, Y.	J Am Soc Nephrol. 26(3):746-53	2015	Diagnostic study in at-risk individuals and healthy controls	37 patients with ADPKD and 58 unaffected (partly from affected families, partly as healthy controls), aged 16-29 years. Of the patients 56% have truncating PKD1, 22% non-truncating PKD1 and 22% PKD2 mutations.	High resolution ultrasound with 3.8 to 8 MHz probes	Sensitivity, specificity, PPV and NPV:<br>≥ 1 renal cyst: 0.973, 0.845, 0.800, 0.980<br>≥ 2 (uni-or bilateral) renal cysts: 0.973, 0.948, 0.923, 0.982<br>≥ 3 (uni-or bilateral) renal cysts: 0.973, 0,983, 0.973, 0.983<br>≥ 4 (uni-or bilateral) renal cysts: 0.973, 0.983, 0.973, 0.983<br>≥ 2 cysts in each kidney: 0.973, 1.0, 1.0, 0.983<br>Compared to conventional ultrasound (Pei 2009): better sensitivity, but slightly lower specificity.	Serious[1]	Not serious	Not serious	Not serious	Exact n < 18 years not mentioned	7
**MRI**													
Imaging-based diagnosis of autosomal dominant polycystic kidney disease	Pei, Y.	J Am Soc Nephrol. 26(3):746-53	2015	Diagnostic study in at-risk individuals and healthy controls	37 patients with ADPKD mutations and 58 unaffected (partly from affected families, partly as healthy controls), Aged 16-29 years. Of the patients 56% have truncating PKD1, 22% non-truncating PKD1 and 22% PKD2 mutations.	T2 weighted, fast-spin echo sequence without gadolinium on 1.5T scanner	Sensitivity, specificity, PPV and NPV:<br>≥ 5 (uni-or bilateral) renal cysts: 1.0, 0.983, 0.974, 1.0<br>> 10 (uni-or bilateral) renal cysts: 1.0, 1.0, 1.0, 1.0<br>≥ 2 cysts in each kidney: 1.0, 0.983, 0.974, 1.0<br><br>Suggest to use >10 cysts as diagnostic criterion in individuals at risk of ADPKD, and stricter <5 criterion for exclusion of ADPKD in potential living related kidney donors.	Serious[1]	Not serious	Not serious	Not serious	Exact n < 18 years not mentioned	7
Title	First Author	Journal	Year	Study design	No & type of patients	Methodological details	Prediction of genetic diagnosis	Risk of bias	Indirectness	Inconsistency	Imprecision	Other con-siderations	Importance (1-9)
**Studies in at-risk children without genetic diagnostics but follow-up ultrasound**													
Renal ultrasonographic evaluation in children at risk of autosomal dominant polycystic kidney disease.	Reed, B.	Am J Kidney Dis. 56(1):50-6	2010	Cohort study of at-risk children	420 children (mean age 8.3 ± 4.2 years)	No genetic diagnostics.<br><br>3 groups: no cysts/ unilateral cysts/ bilateral cysts	Initially 227/420 children did not have cysts, but 10/43 (23%) with FU at age > 15 years and 14/77 (18%) with FU at age ≤ 15 years later developed bilateral cysts.<br><br>Initially 43/420 children only had unilateral cysts. Of these 2/8 (25%) with FU at age > 15 years and 17/26 (65%) with FU at age ≤ 15 years later developed bilateral cysts. However, 3/34 (7%) did not have any cysts on FU.						5
The spectrum of autosomal dominant polycystic kidney disease in children.	Fick, G.M.	J Am Soc Nephrol. 4(9):1654-60.	1994	Cohort study of at-risk children	39 children with follow-up out of 154 children from families with clinical ADPKD	No genetic diagnostics. Diagnostic criterion ≥ 1 cyst.	Of 17 children without cysts, none developed cysts on follow–up.<br>Of 4 children with only 1 cyst, 3 developed bilateral cysts on follow-up (mean 4 years later).<br>Of 22 with ≥ 1 cyst, none had no cysts on follow-up.						5

Title	First Author	Journal	Year	Study design	Nº & type of patients	Methodological details	Prediction of genetic diagnosis	Risk of bias	Indirectness	Inconsistency	Imprecision	Other con-siderations	Importance (1-9)
Autosomal dominant polycystic kidney disease in childhood: a longitudinal study.	Sedman, A.	Kidney Int. 31(4):1000-5	1987	Cohort study of at-risk children	68 children with follow-up out of 154 children from families with clinical ADPKD	No genetic diagnostics.<br><br>Diagnostic criterion > 5 cysts.	Of 37 children without cysts, 3 developed < 5 cysts and 4 developed > 5 cysts after age 18 years.<br>Of 12 children with < 5 cysts, 2 were classified normal and 4 developed > 5 cysts after age 18 years.<br>Of 19 children with > 5 cysts, none were reclassified later.	Unusually severely affected cohort (3 with ESRD)		Very strict diagnostic criterion			5
**Studies in affected children**													
**PKD 1**													
Genotype-phenotype correlation in children with autosomal dominant polycystic kidney disease.	Fencl, F.	Pediatr Nephrol. 24(5):983-9	2009	Cohort study in patients	50 Patients with PKD1 (and positive family history)	mean age at diagnosis 5.4 ± 4.3 years	Total number of cysts: mean 13.39 ± 12.53<br>Bilateral renal cysts: n=43 (86%)<br>Enlarged kidneys: n= 16 (32%)<br>Diameter of the biggest cyst: 16.79 ± 10.65 mm						3
**PKD 2**													
Genotype-phenotype correlation in children with autosomal dominant polycystic kidney disease.	Fencl, F.	Pediatr Nephrol. 24(5):983-9	2009	Cohort study in patients	10 patients with PKD2 (and positive family history)	mean age at diagnosis 7.9 ± 5.0 years	Total number of cysts: mean 3.00 ± 2.10<br>Bilateral renal cysts: n= 3 (30%)<br>Enlarged kidneys: n= 0 (0%)<br>Diameter of the biggest cyst: mean 5.08 ± 1.53 mm						3

Abbreviations: PPV: positive predictive value, NPV: negative predictive value.

Tables S3 Radiological markers of ADPKD progression, with a special focus on children

Table S3a Studies in children correlating radiological markers with disease markers

Title	First Author	Journal	Year	Study design, patients	Disease marker	Radiological marker	Findings	Risk of bias	Indirectness	Inconsistency	Imprecision	Other con-siderations	Importance (1-9)
**Correlation of Renal Volume measures to Blood Pressure in ADPKD children**													
Magnetic resonance imaging of kidney and cyst volume in children with ADPKD.<br><br>PMID: 21115621	Cadnapaphornchai, M.A.	Clin J Am Soc Nephrol. 6(2):369-76	2011	Cohort study.<br><br>77 children with ADPKD, aged 4 -21 years.	Hypertensive (HBP): BP≥95th percentile for age, height, and gender).<br>Normotensive (NBP): BP<95th pctl.	Annual MRI for 5 years (n=302 MRI studies).<br>TKV and cyst volume	TKV and total cyst volume were significantly increased in HBP versus NBP subjects (p<0.0001 and p<0.005).<br>Fractional cyst volume was similar at baseline, but higher in HBP subjects strating from year 2 until end of study (p=0.02).<br>Cyst number was similar at baseline, but higher in HBP children from year 3 until end of study (p<0.01).						7
Increased left ventricular mass in children with autosomal dominant polycystic kidney disease and borderline hypertension.<br><br>PMID: 18716604	Cadnapaphornchai, M.A.	Kidney Int. 74(9):1192-6	2008	Cohort study (baseline in RCT).<br><br>85 children with ADPKD aged 4-21 years.	3 groups:<br>Hypertensive (HBP): BP≥95th percentile.<br>Borderline (BBP): BP 75th-95th percentile.<br>Normotensive: BP < 75th pecercentile.<br><br>All had normal renal function.	2D US:<br>Kidney volumes by ellipsoid formula (mean of both sides).<br>Cyst number recorded up to 15 per kidney.	Correlations in whole group:<br>-systolic BP and log renal volume (r=0.70, P<0.0001)<br>-diastolic BP and renal volume (r=0.52, P<0.0001)<br>Multiple linear regression (covariates age, height, sex) confirmed significant predictive value of systolic BP on log of renal volume (P=0.0002), but diastolic BP was not significant (P=0.2).<br><br>HBP children had larger renal volume than BBP an NBP groups (both p<0.002).<br>Very similar renal volume in NBP and BBP groups.						7
Ambulatory blood pressure correlates with renal volume and number of renal cysts in children with autosomal dominant polycystic kidney disease.<br><br>PMID: 12900587	Seeman, T.	Blood Press Monit. 8(3):107-10.	2003	Cross sectional cohort study.<br><br>62 children with ADPKD, aged 3.4 to 19.4 (mean age 12.3 ± 4.3 years)	24h ambulatory BP monitoring (ABPM): hypertensive (untreated) n=22; n= 40 normotensive.<br><br>Normal renal function (normotensives 111 ± 15, hypertensives 115 ± 16 ml/min*1.73m^2).	2D US:<br>Kidney volumes by ellipsoid formula (mean of both sides).<br>Cyst number in both kidneys combined.	Renal volume was significantly greater in hypertensive than in normotensive children (2.7 ± 2.3 SDS versus 1.2 ± 2.5 SDS, P<0.01) despite similar anthropometric data and renal function.<br>Mean number of cysts was significantly higher in hypertensive patients than in normotensive (35 ± 15 cysts versus 23 ± 14 cysts, P<0.01).<br><br>Renal volume correlated with daytime as well as with night-time systolic and diastolic BP (r=0.41-0.47, P<0.01). Correlations with renal length and the number of renal cysts were somewhat less (r=0.29-0.43, P<0.05 and 0.01, respectively).						7

Title	First Author	Journal	Year	Study design, patients	Disease marker	Radiological marker	Findings	Risk of bias	Indirectness	Inconsistency	Imprecision	Other considerations	Importance (1-9)
Progression of autosomal-dominant polycystic kidney disease in children.<br><br>PMID: 11318935	Fick-Brosnahan, G.M.	Kidney Int. 59(5):1654-62.	2001	115 children from 131 ADPKD families: 182 affected and 127 probably unaffected (ultrasound negative). 108 affected children have ≥ 1 follow-up in childhood (of which n=30 have early severe disease).	Mean Schwartz GFRs at last visit (all ml/min/1.73m²): similar in affected vs unaffected children (135 vs.129), in early severe vs milder disease (132 vs. 136), in BP > 75th vs < 75th pctl (133 vs. 137), with vs without overt proteinuria (135 for both), and with vs without gross hematuria (141 vs. 134).	2D US: Kidney volumes by ellipsoid formula (mean of both sides).<br><br>Fitted regression models for increase of mean renal volume with age.<br><br>Group comparisons normalized to height.	Affected chidren (n=182) have faster renal growth than ultrasound-negative siblings (n=127). No difference by gender.<br>Patients with early severe disease (n=30) had faster renal growth than n=78 without (p<0.05).<br>Affected children with high blood pressure at first and last visit (n=33) had faster renal growth than those with blood pressure < 75th prctl at 1st and last visit (p<0.05).<br>Children with overt proteinuria at the last visit (n=46) had significantly larger kidneys than children without overt proteinuria (n=115. p<0.05, age adjusted p<0.005).<br>No correlation between mean renal volume and GFR.<br>The diameter of the largest cyst correlated with age (r = 0.31, P < 0.0001), but not with pain or gross hematuria. Hypertension was associated with a larger cyst diameter (age-adjusted 3.1 ± 0.2 cm vs. 2.0 ± 0.1 cm, P < 0.0001), but this was not independent from the effects of larger mean renal volumes.	In longitudonal analysis bias towards children with more follow-up visits.					7
**Comparison of MRI and US in ADPKD children**													
3DUS as an alternative to MRI for measuring renal volume in children with autosomal dominant polycystic kidney disease.<br><br>PMID: 29306987	Breysem, L.	Pediatr Nephrol. 33(5):827-835	2018	Cross sectional cohort study. 30 children with ADPKD, aged 8-18 years (median 14 years)	Mean eGFR (Schwartz formula) 109 ± 17 ml/min*1.73 m²	KV by:<br>3D US: ellipsoid method and manual contouring<br>2D US: ellipsoid<br>MRI: manual contouring	All US volumetry methods showed significantly lower mean (± SD) KV (ml), compared to MR (KV_{2DUS}: 159 (±101); $KV_{3DUS\text{-}ellipsoid}$: 169 (±105); $KV_{3DUS\text{-}contour}$: 185 (±110); KV_{MR}: 206 (±130); all p<0.001)).<br><br>Correlation of KV_{MR}: 2DUS: r=0.96; 3DUS-ellipsoid: r=0.89 and 3DUS-contour: r=0.94.<br><br>After correction factor application, Bland-Altman plots showed slightly lower variability and absolute error for $KV_{3DUS\text{-}contour}$ vs. KV_{2DUS} and $KV_{3DUS\text{-}ellipsoid\,;}$ to KV_{MR}.						
Magnetic resonance imaging of kidney and cyst volume in children with ADPKD.<br><br>PMID: 21115621	Cadnapaphornchai, M.A.	Clin J Am Soc Nephrol. 6(2):369-76	2011	Cohort study. 77 children from 4 to 21 years of age	MRI on an annual basis for 5 years.	TKV and cyst volume by MRI (n=302) by stereology and region-based thresholding method.<br>2D US: Total kidney volumes by ellipsoid formula (sum of both sides).	Correlation of MRI and ultrasound TKV r= 0.83 (p not given).<br><br>On average, TKV by ultrasound was 27 ml less than by MRI (p not given, but probably ns). Greater differences were apparent with larger kidneys.	Not all patients have same number of studies (-> bias towards children with more follow-up visits)				No assessment of TCV by ultrasound.	

<u>Abbreviations:</u> ADPKD =Autosomal dominant polycystic kidney disease, BSA = body surface area, CRISP = Consortium for Radiologic Imaging Studies of Polycystic Kidney Disease), CT = computed tomography, HtTKV = Height-adjusted TKV, log-TKV = log-converted TKV, MRI =magnetic resonance imaging, OR= odds ratio, RBF = Renal Blood Flow, TKV = Total kidney volume, US= Ultrasound

Table S3b Studies in adults correlating radiological markers with (prospective loss of) renal function

Title	First Author	Journal	Year	Study design, patients	Renal function measure and range studied	Radiological marker	Outcome prediction	Risk of bias	Indirectness	Inconsistency	Imprecision	Other considerations	Importance (1-9)
**Volume measures (Total kidney volume (TKV), Height TKV (HtTKV) and Renal Cyst Volume (RCV))**													
**Metaanalysis**													
Correlations between renal function and the total kidney volume measured on imaging for autosomal dominant polycystic kidney disease: A systematic review and meta-analysis.<br><br>PMID: 28987699	Jo, W.R.	Eur J Radiol. 95:56-65	2017	Metaanalysis of 18 studies (n=2835 patients) examining correlation of (change of) GFR with (change of) TKV.	Wide range (details not specified)	MRI (14 studies), CT (3 studies) and US (1 study).<br><br>Direct volumetry (15 studies), formula methods (3 studies).	Overall correlation of GFR and TKV: r = -0.520 (95%CI -0.60 to -0.43).<br>No significant difference in correlation of GFR to MR-TKV r=-0.49 (95%CI -0.59 to -0.40) or to CT-TKV r=-0.64 (95%CI -0.75 to -0.53).<br>No significant different in correlation of GFR to volumetry-TKV r=-0.51 (05%CI -0.60 to -0.41) or to volume estimation formula-TKV r=-0.55 (95%CI -0.72 to -0.37).<br><br>Correlation of GFR decline rate to TKV growth rate: r= -0.320 (95% CI -0.54 to -0.10).<br>The quantitative review revealed that higher baseline TKV also correlates to TKV growth rate and GFR decline rate.						9
**Longitudinal studies using MRI**													
Imaging classification of autosomal dominant polycystic kidney disease: a simple model for selecting patients for clinical trials.<br><br>PMID: 24904092	Irazabal, M.V.	J Am Soc Nephrol. 26(1):160-72	2015	Multicenter, retrospective analysis.<br><br>590 patients and 173 patients (CRISP) for validation	≥ 3 eGFR measurements over ≥ 6 months	MRI Images over ≥ 6 months. Classified radiologically as typical (n=538) or atypical (n=52).<br><br>CT and MRI KV measured using stereology (TKVs) and ellipsoid equation (TKVe) (TKVe correlated strongly with TKVs).	A longitudinal mixed regression model to predict eGFR decline showed that log2HtTKV and age significantly interacted with time in typical patients, but not in atypical patients.<br>When 1A-1E classifications were used instead of log2HtTKV, eGFR slopes were significantly different among subclasses and, except for 1A, different from those in healthy kidney donors. The equation derived from the development set predicted eGFR in both validation sets.<br>The frequency of ESRD at 10 years increased from subclass 1A (2.4%) to 1E (66.9%) in the Mayo cohort and from 1C (2.2%) to 1E (22.3%) in the younger CRISP cohort.<br>Class and subclass designations were stable.	Partial overlap of patients with previous CRISP studies					9
A comparison of ultrasound and magnetic resonance imaging shows that kidney length predicts chronic kidney disease in autosomal dominant polycystic kidney disease.<br><br>PMID: 25830764	Bhutani, H.	Kidney Int. 88(1):146-51	2015	Longitudonal cohort study.<br><br>241 patients aged 15–46 years.	Average of 5 iothalamate clearances over a mean follow-up of 9.3 years.<br><br>Creatinine clearance ≥ 70 ml/min.	MRI-htTKV and US-KL intraclass correlations to future CKD stage 3	US and MRI-based htTKV and KL predicted future CKD stage 3 similarly (AUC of 0.87, 0.88, 0.87, and 0.88, respectively).<br>US KL> 16.5 cm and htTKV > 650 ml/min had best cut point for predicting the development of CKD stage 3.<br>“Kidney length alone is sufficient to stratify the risk of progression to renal insufficiency early in ADPKD using either ultrasound or magnetic resonance imaging”						8

Title	First Author	Journal	Year	Study design, patients	Renal function measure and range studied	Radiological marker	Outcome prediction	Risk of bias	Indirectness	Inconsistency	Imprecision	Other con-siderations	Importance (1-9)
Clinical characteristics and disease predictors of a large Chinese cohort of patients with autosomal dominant polycystic kidney disease.<br><br>PMID: 24651850	Chen, D.	PLoS One. 9(3):e92232	2014	Prospective longitudinal observational study.<br><br>541 patients, aged 4-77 years (of which n=23 ≤18 years)	eGFR ≥ 30 ml/min/1.73m^2 (method not given).<br><br>Median follow-up time 14.3 ± 10.6 months.	Sequential MRI for TKV.	Significant negative correlation between baseline TKV and eGFR for the different age categories only between age 19 and 60. For all patients between age 19 and 60 the r amounted to –0.596 (p<0.001).<br>TKV correlated best with eGFR in the age group 19–30 (r= –0.622) and was least in the age group 51–60 (r =–0.382).<br>There was only a very weak correlation between the yearly eGFR change and the yearly TKV volume growth.					Patients ≤18 years excluded from correlation of GFR to TKV.<br><br>TKV not height-adjusted.	6
Kidney volume and function in autosomal dominant polycystic kidney disease.<br><br>PMID: 23864346	Higashihara, E.	Clin Exp Nephrol. 18(1):157-65	2014	Observational study.<br><br>64 patients, mean age 47 years (SD 14.1)	Mean eGFR 60.2 ml/min*1.73 m2 (SD 27.4).<br>CKD 1-2 n=31,<br>CKD 3 n=15,<br>CKD 4 n=11,<br>CKD 5 n=7.	TKV by high-resolution MRI, using a volumetric measurement of cross-sectional imaging, as described in the report from the CRISP study	TKV, HtTKV and log-converted TKV [log-TKV]) correlated with eGFR significantly (log-TKV was most significant (r = -0.6688, p < 0.001)).<br>The eGFR slope correlated negatively with TKV slope (p < 0.05). TKV increased faster and became larger as chronic kidney disease (CKD) stage advanced.<br>If baseline TKV was large, the eGFR slope was steeper (p < 0.05), which suggests that eGFR declines faster in patients with larger kidney volume.						5
Kidney volume and functional outcomes in autosomal dominant polycystic kidney disease.<br><br>PMID: 22344503	Chapman, A.B.	Clin J Am Soc Nephrol. 7(3):479-86	2012	Prospective, observational, longitudinal, multicenter study (CRISP II).<br>241 adults with ADPKD.<br>Mean follow-up 7.9 years	Iothalamate Clearance.<br>Baseline GFR >70 ml/min<br>Endpoint: CKD stage 3 (GFR <60 ml/min per 1.73 m2)	htTKV on MRI	Baseline htTKV correlated to baseline GFR r= -0.22.<br>Baseline htTKV correlated to year 8 GFR r= -0.65.<br>Baseline htTKV ≥600 cc/m predicted the risk of developing endpoint within 8 years with an odds ratio of 1.48 (95% confidence interval: 1.29 - 1.70).<br>In ROC curve analysis, baseline htTKV of 600 cc/m most accurately predicted endpoint with AUC of 0.84 (95% confidence interval: 0.79 - 0.90).<br>htTKV was a better predictor than baseline age, serum creatinine, BUN, urinary albumin, P<0.05).					Partial overlap of patients with CRISP I	7
Increases in kidney volume in autosomal dominant polycystic kidney disease can be detected within 6 months.<br><br>PMID: 18971924	Kistler, A.D.	Kidney Int. 75(2):235-41	2009	Cohort study in 100 young ADPKD patients aged 31.2 ± 6.4 years.	eGFR 109.8 ± 25.5 ml/min (Cockroft-Gault formula).	TKV by manual segmentation volumetry on T1-MRI	TKV negatively correlated with creatinine clearance (r=-0.348, P<0.001).<br>TKV/BSA adjusted for age also correlatied with creatinine clearance (P=0.002).					Probably no correlation of TKV to change in GFR (not mentioned, but data were available)	8
Comparison of methods for determining renal function decline in early autosomal dominant polycystic kidney disease: the consortium of radiologic imaging studies of polycystic kidney disease cohort.<br><br>PMID: 16452494	Rule, A.D.	J Am Soc Nephrol. 17(3):854-62	2006	Prospective outcome study (CRISP).<br><br>234 adults with ADPKD followed annually for 4 visits.<br>Median age 34 years (IQR 25 to 40)	Median (IQR): standardized iothalamate clearance (ml/min per 1.73 m^2) 95 (79 to 115);<br>MDRD equation (ml/min per 1.73 m^2) 79 (63 to 96);<br>Cockcroft-Gault equationc (ml/min) 101 (82 to 126);<br>creatinine clearance (ml/min) 109 (89 to 130)	TKV and cyst volume by MRI	<u>Correlation of log(KV) with slope of:</u><br>standardized iothalamate clearance: r=-030, p< 0.001<br>eGFR by MDRD equation: r=-0.23, p<0.001<br>eGFR by Cockroft-Gault: r=-0.28, p<0.001<br>creatinine clearance: r=-0.19, p=0.003<br><br>Each doubling of kidney volume at baseline was associated with a decline in<br>- iothalamate clearance (OR 2.4; 95% CI 1.5 to 3.7)<br>- eGFR (OR 1.7 [95% CI 1.1 to 2.6] or 2.1 [95% CI 1.4 to 3.3])<br>- creatinine clearance (OR 1.7; 95% CI 1.1 to 2.5).						8

Title	First Author	Journal	Year	Study design, patients	Renal function measure and range studied	Radiological marker	Outcome prediction	Risk of bias	Indirectness	Inconsistency	Imprecision	Other con-siderations	Importance (1-9)
Volume progression in polycystic kidney disease.<br><br>PMID: 16707749	Grantham, J.J.	N Engl J Med. 354(20):2122-30	2006	Three-year prospective study (CRISP I). 241patients (234 with slope determined). Aged15 - 46 years	Iothalamate Clearance. Baseline GFR >70 ml/min	TKV and TCV on MRI (4 measurements over 3 years)	Baseline TKV> 1500 ml in 51 patients was associated with a declining GFR (by 4.33 ± 8.07 ml per minute per year, P<0.001).<br>TKV slope (ml/year) correlates to eGFR slope (ml/year) r=−0.186 (P = 0.005).<br>In patients < 30 years, eGFR slope was:<br>TKV < 750 ml: + 288 ± 12 ml/min/year<br>TKV 750-1500: -0.38± 7.66 ml/min/year<br>TKV > 1500 ml: -2.69 ± 10.2 ml/min/year					Same patients as Rule et al 2006	9
**Cross-sectional studies using MRI**													
Relationship between renal function and renal volume in autosomal dominant polycystic kidney disease: cross-sectional study.<br>[Article in English, Spanish]<br><br>PMID: 26518512	Torres-Sánchez, M.J.	Rev Clin Esp. 216(2):62-7	2016	Cross sectional study. 67 adults with ADPKD (59.7% women, average age of 48±14.4 years)	eGFR by MDRD and Cockroft Gault formulas<br>CKD stage 1: 29.9%, stage2: 19.4%, stage 3 10.4% 3A, stage 3B 9%, stage 4: 20.9%, stage 5: 9%.	TKV on MRI (manual segmentation)	Correlation of TKV with<br>- serum creatinine r = 0.540, p < 0.05<br>- serum urea r = 0.485, p< 0.05<br><br>Patients with eGFR ≥60 had significantly smaller TKV than those with < 60 mL/min (p < 0.05):<br>1048.04 ± 533.35 mLand 2297.66 ± 1354.86 mL.					Wide range of eGFRs examined	7
Short-term Effects of Tolvaptan in Individuals With Autosomal Dominant Polycystic Kidney Disease at Various Levels of Kidney Function.<br><br>PMID: 25600953	Boertien, W.E.	Am J Kidney Dis. 65(6):833-41	2015	Short interventional study. 3 weeks tolvaptan in 27 adults with ADPKD (52% men; aged 46±10 years)	GFR by (125)I-iothalamate clearance. mGFR, 69±39mL/min; TKV, 2.15 [IQR, 1.10-2.77] L)	TKV, by magnetic resonance imaging;	Correlation of mGFR with ln(TKV)<br>at baseline: r= -0.566 (p= 0.002)<br>on tolvaptan: r= -0.574 (p=0.002)	Small study				Observation time to short for prospective evaluation	5
Urinary biomarkers at early ADPKD disease stage.<br><br>PMID: 25875363	Petzold, K.	PLoS One. 10(4):e0123555	2015	Cross sectional biomarker study. 139 adults with ADPKD, mean age 31 ± 7 years	eGFR calculated with CKD-EPI formula > 70 ml/min*1.73m[2].<br>Mean eGFR 93 ± 19 ml/min*1.73m[2]	TKV from MRI	Correlation of eGFR<br>with TKV r = -0.44508, p<0.05<br>with htTKV r = -0.45531, p<0.05<br><br>simple linear regression to predict log/htTKV):<br>eGFR β = -0.45968, p<.0001. Prognostic power of eGFR to predict htTKV is 20.6% (R^2 = 0.2055)						5
Analysis of baseline parameters in the HALT polycystic kidney disease trials.<br><br>PMID: 22205355	Torres, V.E.	Kidney Int. 81(6):577-85	2012	Baseline data from 2 RCTs (HALT A (n=558) and B (n=486))	eGFR by CKD-EPI formula.	TKV on MRI in study A	Correlation of baseline ln(htTKV) with eGFR N=528, r=-0.339, p<0.0001<br><br>Final regression model to predict ln(htTKV) (n=486) includes only BSA, ln(urine albumin) and eGFR (β=-0.286, p<0.001).<br>Final regression model to predict eGFR (n=265) includes age, ln(htTKV) (β=-0.18, p<0.001) and RBF (β=0.30, p<0.001).					Very large cohort, but (pre-) hypertensive patients only	7

Title	First Author	Journal	Year	Study design, patients	Renal function measure and range studied	Radiological marker	Outcome prediction	Risk of bias	Indirectness	Inconsistency	Imprecision	Other con-siderations	Importance (1-9)
Short-term effects of tolvaptan on renal function and volume in patients with autosomal dominant polycystic kidney disease.<br><br>PMID: 21544064	Irazabal, M.V.	Kidney Int. Aug;80(3): 295-301	2011	Short interventional study.<br>1 week tolvaptan in 20 patients ADPKD	eGFR by Cockcroft–Gault equation.<br>Mean eGFR 69.3±34.8 ml/min*1.73 m^2	TKV (automatic measurements) on MRI	Correlation of GFR and renal volume<br>Baseline: r = 0.788 (p<0.001)	Small study					5
Renal structure in early autosomal-dominant polycystic kidney disease (ADPKD): The Consortium for Radiologic Imaging Studies of Polycystic Kidney Disease (CRISP) cohort.<br><br>PMID: 12911554	Chapman, A.B.	Kidney Int. Sep;64(3): 1035-45.	2003	Baseline measures of prospective study (CRISP).<br>241 adults with ADPKD (145 women and 96 men)	GFR by (125)I-iothalamate clearance.<br>GFR > 70 ml/min*1.73 m^2	TKV, by magnetic resonance imaging	GFR was inversely related to:<br>Age-adjusted renal volume ($r = -0.31$, $P < 0.0001$)<br>Cyst volume ($r = -0.36$, $P < 0.0001$)<br>% cyst volume ($r = -0.35$, $P < 0.0001$)						7
Reliability of magnetic resonance imaging for measuring the volumetric indices in autosomal-dominant polycystic kidney disease: correlation with hypertension and renal function.<br><br>PMID: 16636585	Lee, Y.R.	Nephron Clin Pract 103(4):c173-80	2006	Cross sectional study.<br><br>56 adults with ADPKD	eGFR from Cockroft Gault formula corrected for BSA.<br>Mean eGFR 62 ± 27 (n=33, 63% eGFR < 60ml/min*1.73 m^2)	TKV and TCV on MRI (automated measurements), corrected for BSA	eGFR correlated with:<br>-TKV BSA r = –0.56 (p < 0.0001),<br>-TCVBSA , r = –0.60 (p < 0.0001)<br>-%cyst volume r = –0.57 (p < 0.0001)<br>(After age adjustment, these correlations remained signifi cant:<br>-TKV BSA r = –0.53 (p < 0.0001),<br>-TCVBSA , r = –0.57 (p < 0.0001)<br>-%cyst volume r = –0.46 (p < 0.0001)<br>No association between the Ccr and parenchymal volume.						7
**Longitudinal non-MRI studies**													
The relationship between renal volume and renal function in autosomal dominant polycystic kidney disease.<br><br>PMID: 21431900	Tokiwa, S.	Clin Exp Nephrol. 15(4):539-45	2011	Cross sectional study in 73 adults with ADPKD.<br><br>Median age 48 years, age range 21–72 years	eGFR by simplified MDRD formula with coefficient for Japanese population.<br>Median eGFR 55.3 ml/min/1.73 m^2 (range 7.2–120.6).	TKV by ellipsoid formula (MRI, CT or ultrasound).	Correlation of eGFR with TKV (all observations combined): r = -0.313, p < 0.0001<br>but no independent prediction in multiple regression<br><br>Patients with eGFR < 60 had significantly smaller TKV than those with eGFR > 60 ml/min/1.73 m^2:<br>2306.8 ± 1394.5 vs. 1640.9 ± 1334.5 ml, p = 0.005.<br><br>Correlation of ΔGFR/y with ΔTKV/y<br>r = -0.674, p < 0.0001<br>ΔTKV was significant independent predictor of ΔeGFR in multiple regression				TKV measurements by different methods	Multiple data points for single patients allowed.	6
Relationship between renal volume growth and renal function in autosomal dominant polycystic kidney disease: a longitudinal study.<br><br>PMID: 12046022	Fick-Brosnahan, G.M.	Am J Kidney Dis.39(6):1127-34.	2002	Longitudonal cohort study.<br><br>229 adult s with ADPKD	eGFR calculated with MDRD formula	Sequential ultrasound.<br>Renal volume by ellipsoid formula.	Correlation of GFR and renal volume<br>Baseline r = -0.53<br>At follow-up r = -0.50 (for both men and women)<br><br>Multiple linear regression showed a significant relationship between rate of change in GFR and renal volume growth rate, initial renal volume, proteinuria, and age at entry.						7

Title	First Author	Journal	Year	Study design, patients	Renal function measure and range studied	Radiological marker	Outcome prediction	Risk of bias	Indirectness	Inconsistency	Imprecision	Other con-siderations	Importance (1-9)
Volumetric determination of progression in autosomal dominant polycystic kidney disease by computed tomography.<br><br>PMID: 11115083	Sise, C.	Kidney Int. 58(6):2492-501	2000	Retrospective cohort study of 10 patients with ADPKD and 2 CTs ≥ 3 years apart	10 patients with mean age at the initial 33.8 years, and clearances 60 to 135 mL/min/1. 73 m2).<br><br>Outcome analysis for n=9, as 1 patient received partial nephrectomy affecting outcome.	CT (n=10): TKV (Vt) by manual tracing of renal areas in contiguous 5 to 10 mm thick axial CT slices.<br>Contrast CT (n=8) densitometry: noncystic parenchymal area (Vp), total cyst volume (Vc) = Vt-Vp.	5 patients with ESRD: mean age 35.8 years, mean follow up 11,2 years.<br>4 patients without ESRD: 29.3 years, man follow-up 8.7 years.<br><br>Group differences: initial kidney volume, rate of kidney enlargement greater in ESRD group (both ns),<br>Initial cyst/kidney volume ratio (combined for both kidneys) higher in ESRD group (p=0.02) (all ≥ 0.43)	ESRD group older and followed up for longer.			Small, retrospective study		5
Quantification and longitudinal trends of kidney, renal cyst, and renal parenchyma volumes in autosomal dominant polycystic kidney disease.<br><br>PMID: 10906164	King, B.F.	J Am Soc Nephrol. 11(8):1505-11	2000	Pilot study on retrospective cohort.	9 patients with serum creatinine < or = 1.3 mg/dl and/or an initial iothalamate clearance > or = to 60 ml/min per 1.73 m^2.<br>eGFR slope (stable in n=1, declined in n=8).	fast electron-beam **CT:** volumetric determinations of TKV, renal cyst volume, and renal parenchymal volumes by manual segmentation and semiautomatic threshold approach. 3 CTs over 3 weeks and then follow-up 8 years later.	At entry: GFR correlated negatively with total renal (r=-0.3, p=0.28) or cyst volumes (r=-0.64, p=0.06) and positively with renal parenchymal volume (r=+0.59, p=0.09). (all p=ns!)<br><br>Follow-Up: rate of decline in GFR correlated significantly with rate of increase in renal cyst volume (r=-0.71 p=0.046), and non-signigicantly with increase in renal volume (r=-0.48, p=0.19), the %/year chane in cyst volume (r=-0.21, p=0.6= and decrease of parenchymal volume (r=0.33, p=0.4).	Selected by follow-up.			Very small group		3
**Cross-sectional non-MRI studies**													
Urinary N-acetyl-β-D glucosaminidase as a surrogate marker for renal function in autosomal dominant polycystic kidney disease: 1 year prospective cohort study.<br><br>PMID: 22935351	Park, H.C.	BMC Nephrol. 13:93	2012	Prospective biomarker study.<br><br>270 adults with ADPKD, mean age 34.9 ± 9.5 years	Estimated GFR by: MDRD formula 85.0 ± 24.7 mL/min/1.73 m^2<br>and CKD-EPI GFR 91.9 ± 23.3 mL/min/1.73 m^2	Contrast enhanced CT<br>TKV by modified ellipsoid method.	<u>Correlation of eGFR to log(TKV):</u><br>r^2 = 0.334, P < 0.001						5
Volume of polycystic kidneys during reduction of renal function.<br><br>PMID: 7314325	Thomsen, H.S.	Urol Radiol. 3(2):85-9	1981	Cross sectional study in 43 patients with ADPKD aged 16 - 66 years and 12 controls (27-45 years)	24h endogenous creatinine clearance ranged from 8 to 130 ml/min	CT TKV	Correlation of creatinine clearance and renal volume r=-0.706, p<0.001<br><br>Patients with normal renal function: mean TKV 1,212 cm^3 (SD ± 411)<br>Patients with severely decreased function mean TKV 2,053 cm^3 (SD ±698), p< 0.01 to patients with normal renal function.<br>Controls: 497 cm^3 (SD ± 58) cm^3					Old study	4

Title	First Author	Journal	Year	Study design, patients	Renal function measure and range studied	Radiological marker	Outcome prediction	Risk of bias	Indirectness	Inconsistency	Imprecision	Other considerations	Importance (1-9)
**Renal texture, blood flow and vascular resistance**													
Image texture features predict renal function decline in patients with autosomal dominant polycystic kidney disease.<br><br>PMID: 28532709	Kline, T.L.	Kidney Int. 92(5):1206-1216	2017	Retrospective cohort study.<br><br>122 patients from CRISP	eGFR over 70 mL/min/1.73m² at baseline.<br>Endpoint: progression to CKD stage 3A and 3B. 30% reduction in eGFR at 8 year follow-up.	T2-weighted MRIs 9 distinct image texture features for each patient.	Adding 3 of 9 texture variables in a multiple linear regression model predicting percent change in eGFR (using age, eGFR, and HtTKV) improved Pearson correlation coefficient from -0.51 to -0.70.						7
Analysis of baseline parameters in the HALT polycystic kidney disease trials.<br><br>PMID: 22205355	Torres, V.E.	Kidney Int. 81(6):577-85	2012	Baseline data from 2 RCTs (HALT A (n=558) and B (n=486))	eGFR by CKD-EPI formula.	TKV and RBF on MRI in study A	Correlation of baseline RBF with eGFR N=265, r=-0.181, p= 0.0032<br><br>Final regression model to predict eGFR (n=265) includes age, ln(htTKV) (β=-0.18, p<0.001) and RBF (β=0.30, p<0.001).					Very large cohort, but (pre-) hypertensive patients only	8
Magnetic resonance measurements of renal blood flow and disease progression in autosomal dominant polycystic kidney disease.<br><br>PMID: 17699395	Torres, V.E.	Clin J Am Soc Nephrol. 2(1):112-20	2007	Cohort study<br><br>131 patients from CRISP aged 15-46 years	Iothalamate clearances >70 mL/min,	Measurements of RBF and (TKV and TCV) by MRI	Baseline TKV and RBF were independent predictors of TKV and TCV slopes (structural disease progression).<br><br>TKV, TCV, RVR, and MAP were negatively and RBF positively correlated with GFR slopes.<br><br>TKV and RBF were independent predictors of GFR decline (functional disease progression).<br><br>GFR negatively correlated with TKV and TCV slopes.						9
Magnetic resonance measurements of renal blood flow as a marker of disease severity in autosomal-dominant polycystic kidney disease.<br><br>PMID: 14633145	King, B.F.	Kidney Int. 64(6):2214-21	2003	Cohort study.<br><br>127 patients in CRISP I	Iothalamate clearances >70 mL/min	Renal vascular resistance (RVR) calculated from MR blood flow sequences utilizing 2D cine phase-contrast breath-hold pulse sequence perpendicular to the renal arteries.	GFR inversely correlated with kidney volume and positively correlated with RBF (r = 0.52, P < 0.001)<br><br>Regression analysis showed that age, diagnosis of hypertension, anatomic parameters and hemodynamic parameters (r^2 for RBF = 0.34) were significant predictors of GFR. Multiple linear regression analysis identified age and hemodynamic parameters only as separate predictors of GFR.					RBF stronger predictor of GFR than TKV.	7

Abbreviations: ADPKD =Autosomal dominant polycystic kidney disease, BSA = body surface area, CRISP = Consortium for Radiologic Imaging Studies of Polycystic Kidney Disease), CT = computed tomography, HtTKV = Height-adjusted TKV, log-TKV = log-converted TKV, MRI =magnetic resonance imaging, OR= odds ratio, RBF = Renal Blood Flow, TKV = Total kidney volume, US= Ultrasound

Table S4 Incidence of complications and unusual presentations of ADPKD in children and young people

Title	First Author	Journal	Year	Study design	Nº & type of patients	Incidence	Details	Risk of bias	Indirectness	Inconsistency	Imprecision	Other con-siderations	Importance (1-9)
**Renal complications**													
**Hypertension**													
Hypertension in autosomal dominant polycystic kidney disease: a meta-analysis<br><br>(PMID 27288429)	Marlais, M	Arch Dis Child 101: 1142-1147	2016	Systematic review and meta-analysis	928 children across 14 studies	20% (95% CI 15-27%) prevalence of hypertension	Meta-regression with mean age of child as the covariate shows that the prevalence of hypertension increases as the mean age in the study increases (p=0.0128).<br><br>Meta-regression of prevalence of hypertension against prevalence of proteinuria shows no evidence of a relationship (p=0.2641).	Not serious	Not serious	Not serious	Not serious	High degree of methodological heterogeneity noted across studies	8
Long-term outcomes in patients with very-early onset autosomal dominant polycystic kidney disease<br><br>(PMID 27548646)	Nowak, KL	Am J Nephrol 44: 171-178.	2016	Longitudinal retrospective cohort study	140 children with ADPKD, median age of follow up 16 years	63/140 (45%) prevalence of hypertension	Study split into 70 VEO (diagnosed before 18 months of age) and 70 non-VEO children with ADPKD<br><br>VEO patients significantly more likely to develop hypertension than non-VEO patients (p<0.0001).	Serious – possible age above 18yrs	Not serious	Serious – possible due to higher median age and selected (VEO) population	Not serious	Note that some individuals in this study will be >18yrs at last follow up	7
**Proteinuria**													
Factors relating to urinary protein excretion in children with autosomal dominant polycystic kidney disease<br><br>(PMID 9773792)	Sharp, C	JASN 9: 1908-1914	1998	Observational study	103 children with ADPKD from 189 children at risk of ADPKD, mean age 11.2 years	24/103 (23%) had overt proteinuria	No correlation between proteinuria and hypertension<br><br>Children with ADPKD had significantly higher albumin excretion rates than non-ADPKD children	Not serious	Not serious	Not serious	Not serious		6
Progression of autosomal-dominant polycystic kidney disease in children<br><br>(PMID 11318935)	Fick-Brosnahan, GM	Kidney Int 59: 1654-1662	2001	Longitudinal observational study	185 children with ADPKD from 312 children at risk of ADPKD	46/161 (29%) with proteinuria	Proteinuria was not correlated with hypertension; all children with proteinuria had normal renal function.	Not serious	Not serious	Not serious	Not serious		7
Hypertension in autosomal dominant polycystic kidney disease: a meta-analysis<br><br>(PMID 27288429)	Marlais, M	Arch Dis Child 101: 1142-1147	2016	Systematic review and meta-analysis	509 children across 8 studies reporting prevalence of proteinuria	20% (95% CI 9-40%) prevalence of proteinuria	Prevalence of proteinuria was a secondary outcome in this meta-analysis.<br><br>Meta-regression of prevalence of hypertension against prevalence of proteinuria shows no evidence of a relationship (p=0.2641).	Not serious	Not serious	Not serious	Not serious	High degree of methodological heterogeneity noted across studies	8
**Reduced GFR (<90 mL/min/1.73 m²)**													
Spectrum of autosomal dominant polycystic kidney disease in children<br><br>(PMID 8011974)	Fick, GM	JASN 4: 1654-1660	1994	Observational study	62 children affected by ADPKD from 140 children at 50% risk of ADPKD	1/62 (2%)	One child had raised creatinine and progressed to ESRD in childhood	Not serious	Not serious	Not serious	Not serious		7

Title	First Author	Journal	Year	Study design	Nº & type of patients	Incidence	Details	Risk of bias	Indirectness	Inconsistency	Imprecision	Other con-siderations	Importance (1-9)
Glomerular hyperfiltration and renal progression in children with autosomal dominant polycystic kidney disease (PMID 21903987)	Helal, I	CJASN 6: 2439-2443	2011	Observational study (with longitudinal subset)	180 children with ADPKD aged 4-18 followed up as part of Denver cohort	Absolute incidence not described in paper	Study split into 32 children with glomerular hyperfiltration (GH) and 148 children without GH<br>In 140 children with ADPKD where there was longitudinal data available, the children with GH demonstrated a faster decline in creatinine clearance than the group without GH	Not serious	Serious – paper not directed at this complication	Not serious	Not serious	Note the aim of this paper was not reduced GFR	5
Hypertension in autosomal dominant polycystic kidney disease: a meta-analysis (PMID 27288429)	Marlais, M	Arch Dis Child 101: 1142-1147	2016	Systematic review and meta-analysis	226 children across 5 studies reporting prevalence of reduced GFR	8% (95% CI 2-26%) prevalence of reduced GFR	Reduced GFR was a secondary outcome in this meta-analysis.	Not serious	Not serious	Not serious	Not serious	High degree of methodological heterogeneity noted across studies	8
Long-term outcomes in patients with very-early onset autosomal dominant polycystic kidney disease (PMID 27548646)	Nowak, KL	Am J Nephrol 44: 171-178.	2016	Longitudinal retrospective cohort study	140 children with ADPKD, median age of follow up 16 years	40/140 (29%) prevalence of reduced GFR (<90ml/min/1.73m^2)	Study split into 70 VEO (diagnosed before 18 months of age) and 70 non-VEO children with ADPKD<br>VEO patients significantly more likely to have reduced GFR than non-VEO patients (p=0.034).<br>4 cases of ESRD in study follow up period, all in VEO group	Serious – possible age above 18yrs	Not serious	Serious – possible due to higher median age and selected (VEO) population	Not serious	Note that some individuals in this study will be >18yrs at last follow up	7
**Urinary Frequency/ Nocturia/ Enuresis**													
Spectrum of autosomal dominant polycystic kidney disease in children (PMID 8011974)	Fick, GM	JASN 4: 1654-1660	1994	Prospective observational study	62 children affected by ADPKD from 140 children at 50% risk of ADPKD	15/62 (24%)	Proportion complaining of urinary frequency	Not serious	Not serious	Not serious	Not serious		7
Similar renal outcomes in children with ADPKD diagnosed by screening or presenting with symptoms (PMID 20683618)	Mekahli, D	Pediatr Nephrol 25: 2275-2282	2010	Retrospective chart review	47 children identified in specialist centre in UK, 16 presented with symptoms, 31 asymptomatic	1/16 (6%)	Out of 16 children presenting with symptoms, 1 child had enuresis at presentation<br>Unable to include data for 31 asymptomatic children as follow up data for this complication is not included in paper	Serious – small single centre study in specialized centre	Not serious	Not serious	Not serious	Note that all 16 of these children in this subset of the paper presented with symptoms	5
**Flank-/ Back-/ Abdominal Pain**													
Spectrum of autosomal dominant polycystic kidney disease in children (PMID 8011974)	Fick, GM	JASN 4: 1654-1660	1994	Prospective observational study	62 children affected by ADPKD from 140 children at 50% risk of ADPKD	10/62 (16%)	Proportion complaining of back pain	Not serious	Not serious	Not serious	Not serious		7

Title	First Author	Journal	Year	Study design	Nº & type of patients	Incidence	Details	Risk of bias	Indirectness	Inconsistency	Imprecision	Other con-siderations	Importance (1-9)
Similar renal outcomes in children with ADPKD diagnosed by screening or presenting with symptoms (PMID 20683618)	Mekahli, D	Pediatr Nephrol 25: 2275-2282	2010	Retrospective chart review	47 children identified in specialist centre in UK, 16 presented with symptoms, 31 asymptomatic	5/16 (31%)	Out of 16 children presenting with symptoms, 5 children had abdominal or back pain at presentation<br>Unable to include data for 31 asymptomatic children as follow up data for this complication is not included in paper	Serious – small single centre study in specialized centre	Not serious	Not serious	Not serious	Note that all 16 of these children in this subset of the paper presented with symptoms	5
Glomerular hyperfiltration and renal progression in children with autosomal dominant polycystic kidney disease (PMID 21903987)	Helal, I	CJASN 6: 2439-2443	2011	Observational study	180 children with ADPKD aged 4-18 followed up as part of Denver cohort	28/180 (16%)	Proportion of children with refcurrent flank pain at baseline<br>Study split into 32 children with glomerular hyperfiltration (GH) and 148 children without GH	Not serious	Serious – paper not directed at this complication	Not serious	Not serious	Only baseline data for this complication presented	5
**History of urinary tract infections**													
Spectrum of autosomal dominant polycystic kidney disease in children (PMID 8011974)	Fick, GM	JASN 4: 1654-1660	1994	Prospective observational study	62 children affected by ADPKD from 140 children at 50% risk of ADPKD	9/62 (15%)	Proportion with a history of UTIs	Not serious	Not serious	Not serious	Not serious		7
Prognosis of autosomal dominant polycystic kidney disease diagnosed in utero or at birth (PMID 17124604)	Boyer, O	Pediatr Nephrol 22: 380-388	2007	Retrospective note review	26 consecutive children with ADPKD, included in neonatal period	5/26 (18%)	Proportion with at least one UTI during follow up	Serious – single centre study	Not serious	Not serious	Not serious	Only children diagnosed antenatally or by birth included	5
Genotype-phenotype correlation in children with autosomal dominant polycystic kidney disease (PMID 19194729)	Fencl, F	Pediatr Nephol 24: 983-989	2009	Retrospective note review	60 children with PKD and confirmed genetic testing from cohort of 260 children with ADPKD	7/60 (12%)	Study split into 50 children with PKD1 and 10 children with PKD2, 12% in PKD1 group had UTI and 10% in PKD2 group had UTI	Serious – small single centre study in specialized centre	Not serious	Not serious	Not serious		6
Similar renal outcomes in children with ADPKD diagnosed by screening or presenting with symptoms (PMID 20683618)	Mekahli, D	Pediatr Nephrol 25: 2275-2282	2010	Retrospective chart review	47 children identified in specialist centre in UK, 16 presented with symptoms, 31 asymptomatic	6/16 (38%)	Out of 16 children presenting with symptoms, 6 children presented with UTIs<br>Unable to include data for 31 asymptomatic children as follow up data for this complication is not included in paper	Serious – small single centre study in specialized centre	Not serious	Not serious	Not serious	Note that all 16 of these children in this subset of the paper presented with symptoms	5
Glomerular hyperfiltration and renal progression in children with autosomal dominant polycystic kidney disease (PMID 21903987)	Helal, I	CJASN 6: 2439-2443	2011	Observational study	180 children with ADPKD aged 4-18 followed up as part of Denver cohort	43/180 (24%)	Proportion of children with history of cyst infection at baseline<br>Study split into 32 children with glomerular hyperfiltration (GH) and 148 children without GH	Not serious	Serious – paper not directed at this complication	Not serious	Not serious	Only baseline data for this complication presented	5

Title	First Author	Journal	Year	Study design	Nº & type of patients	Incidence	Details	Risk of bias	Indirectness	Inconsistency	Imprecision	Other considerations	Importance (1-9)
**Hematuria**													
Spectrum of autosomal dominant polycystic kidney disease in children<br><br>(PMID 8011974)	Fick, GM	JASN 4: 1654-1660	1994	Prospective observational study	62 children affected by ADPKD from 140 children at 50% risk of ADPKD	6/62 (10%)	Proportion with a history of haematuria	Not serious	Not serious	Not serious	Not serious		7
Progression of autosomal-dominant polycystic kidney disease in children<br><br>(PMID 11318935)	Fick-Brosnahan, GM	Kidney Int 59: 1654-1662	2001	Longitudinal observational study	185 children with ADPKD from 312 children at risk of ADPKD	19/181 (10%)	Proportion with gross haematuria	Not serious	Not serious	Not serious	Not serious		7
Autosomal-dominant polycystic kidney in infancy and childhood: Progression and outcome<br><br>(PMID 16221221)	Shamshirsaz, A	Kidney Int 68: 2218-2224	2005	Observational study	199 children with ADPKD, recruited from cohort of families at risk	30/199 (15%))	Proportion with gross haematuria<br><br>Study population split into 46 children with VEO ADPKD (<18 months) and 153 children with onset 18mo-18yrs	Not serious	Not serious	Not serious	Not serious		7
Prognosis of autosomal dominant polycystic kidney disease diagnosed in utero or at birth<br><br>(PMID 17124604)	Boyer, O	Pediatr Nephrol 22: 380-388	2007	Retrospective note review	26 consecutive children with ADPKD, included in neonatal period	2/26 (8%)	Proportion with at least one episode of gross haematuria	Serious – single centre study	Not serious	Not serious	Not serious	Only children diagnosed antenatally or by birth included	5
Genotype-phenotype correlation in children with autosomal dominant polycystic kidney disease<br><br>(PMID 19194729)	Fencl, F	Pediatr Nephol 24: 983-989	2009	Retrospective note review	60 children with PKD and confirmed genetic testing from cohort of 260 children with ADPKD	3/60 (5%)	Study split into 50 children with PKD1 and 10 children with PKD2, 6% in PKD1 group had macroscopic haematuria, none in PKD2 group	Serious – small single centre study in specialized centre	Not serious	Not serious	Not serious		6
Similar renal outcomes in children with ADPKD diagnosed by screening or presenting with symptoms<br><br>(PMID 20683618)	Mekahli, D	Pediatr Nephrol 25: 2275-2282	2010	Retrospective chart review	47 children identified in specialist centre in UK, 16 presented with symptoms, 31 asymptomatic	1/16 (6%)	Out of 16 children presenting with symptoms, 1 child had haematuria at presentation<br><br>Unable to include data for 31 asymptomatic children as follow up data for this complication is not included in paper	Serious – small single centre study in specialized centre	Not serious	Not serious	Not serious	Note that all 16 of these children in this subset of the paper presented with symptoms	5
Glomerular hyperfiltration and renal progression in children with autosomal dominant polycystic kidney disease<br><br>(PMID 21903987)	Helal, I	CJASN 6: 2439-2443	2011	Observational study	180 children with ADPKD aged 4-18 followed up as part of Denver cohort	26/180 (14%)	Proportion of children with history of macroscopic haematuria at baseline<br><br>Study split into 32 children with glomerular hyperfiltration (GH) and 148 children without GH	Not serious	Serious – paper not directed at this complication	Not serious	Not serious	Only baseline data for this complication presented	5

Title	First Author	Journal	Year	Study design	Nº & type of patients	Incidence	Details	Risk of bias	Indirectness	Inconsistency	Imprecision	Other con-siderations	Importance (1-9)
**Severe/ARPKD-like presentation/Oligohydramnios**													
Childhood onset autosomal dominant polycystic kidney disease in sibs: clinical picture and recurrence risk<br><br>(PMID 8411032)	Zerres, K	J Med Genet 30: 583-588	1993	Observational study	38 affected siblings, prospective registry, mean age of diagnosis 4.2 years	5/38 (13%) died postnatally from respiratory failure<br><br>5/38 (13%) developed renal insufficiency or required renal transplant	Postnatal death from respiratory failure attributed to ADPKD in paper, further details unclear.<br><br>Renal insufficiency defined as creatinine >100umol/L	Serious – selected population	Serious – study was not designed to detect VEO ADPKD	Not serious	Not serious	Appears to be a phenotypically very severe group, possibility of compound heterozygote raised in discussion	3
Prognosis of autosomal dominant polycystic kidney disease diagnosed in utero or at birth<br><br>(PMID 17124604)	Boyer, O	Pediatr Nephrol 22: 380-388	2007	Retrospective note review	26 consecutive children with ADPKD, included in neonatal period	5/26 (19%) had oligohydramnios antenatally<br><br>6/26 (23%) had enlarged palpable kidneys at birth	1 neonate with oligohydramnios presented with severe neonatal symptoms, pneumothorax, hypertension, renal insufficiency<br><br>2 further neonates with oligohydramnios suffered a reversible pneumothorax	Serious – single centre study	Not serious	Not serious	Not serious	Only children diagnosed antenatally or by birth included	5
Comprehensive PKD1 and PKD2 mutation analysis in prenatal autosomal dominant polycystic kidney disease<br><br>(PMID 26139440)	Audrezet, M-P	JASN 27: 722-729	2016	Retrospective study	42 patients, 40 patients diagnosed with ADPKD prenatally	7/34 (21%) had oligo/an-hydramnios	Anhydramnios in 1 patient, oligohydramnios in 6, normal amniotic fluid in 27 patients (not known in 6).<br><br>1 neonate presented with respiratory symptoms after birth related to lung hypoplasia.<br><br>Mean kidney size +3.9SD (range 0-+14SD) before birth.<br><br>Termination of pregnancy was performed at parental request in 4 cases.<br><br>Additional PKD variation inherited from unaffected parent was detected in 15/42 patients (37%).	Not serious	Not serious	Not serious	Not serious	As stated, this is a selected cohort diagnosed prenatally or at birth	6
**Extrarenal complications**													
**Hepatic cysts**													
Spectrum of autosomal dominant polycystic kidney disease in children<br><br>(PMID 8011974)	Fick, GM	JASN 4: 1654-1660	1994	Prospective observational study	62 children affected by ADPKD from 140 children at 50% risk of ADPKD	1/62 (2%)	One child had a single liver cyst	Not serious	Not serious	Not serious	Not serious		7
Phenotypic heterogeneity in paediatric autosomal dominant polycystic kidney disease at first presentation: a single-center, 20 year review<br><br>(PMID 14750095)	Tee, JB	Am J Kidney Dis 43: 296-303.	2004	Retrospective case note review	55 children with ADPKD presenting over 20 years to one institution, mean age 8.7 years	0/55 (0%)	No children were found to have hepatic, splenic or pancreatic cysts on USS.<br><br>Unable to extract data on haematuria/UTI/abdominal pain as only data at presentation is presented, rather than comprehensive data.	Serious – retrospective case note review, single centre	Not serious	Not serious	Not serious		5

Title	First Author	Journal	Year	Study design	Nº & type of patients	Incidence	Details	Risk of bias	Indirectness	Inconsistency	Imprecision	Other considerations	Importance (1-9)
Autosomal-dominant polycystic kidney in infancy and childhood: Progression and outcome<br><br>(PMID 16221221)	Shamshirsaz, A	Kidney Int 68: 2218-2224	2005	Observational study	199 children with ADPKD, recruited from cohort of families at risk	3/199 (2%)	Proportion with liver cysts on ultrasound<br><br>Study population split into 46 children with VEO ADPKD (<18 months) and 153 children with onset 18mo-18yrs	Not serious	Not serious	Not serious	Not serious		7
Prognosis of autosomal dominant polycystic kidney disease diagnosed in utero or at birth<br><br>(PMID 17124604)	Boyer, O	Pediatr Nephrol 22: 380-388	2007	Retrospective note review	26 consecutive children with ADPKD, included in neonatal period	0/26 (0%)	No children had cysts on liver ultrasound or any other extra-renal manifestations	Serious – small single centre study in specialized centre	Not serious	Not serious	Not serious	Only children diagnosed antenatally or by birth included	5
Genotype-phenotype correlation in children with autosomal dominant polycystic kidney disease<br><br>(PMID 19194729)	Fencl, F	Pediatr Nephol 24: 983-989	2009	Retrospective note review	60 children with ADPKD and confirmed genetic testing from cohort of 260 children with ADPKD	0/60 (0%)	No children had liver or pancreas cysts on ultrasound	Serious – small single centre study in specialized centre	Not serious	Not serious	Not serious		6
Similar renal outcomes in children with ADPKD diagnosed by screening or presenting with symptoms<br><br>(PMID 20683618)	Mekahli, D	Pediatr Nephrol 25: 2275-2282	2010	Retrospective chart review	47 children identified in specialist centre in UK, 16 presented with symptoms, 31 asymptomatic	2/47 (4%)	2 children had a solitary liver cyst<br><br>In addition, 1 child had a solitary pancreatic cyst	Serious – small single centre study in specialized centre	Not serious	Not serious	Not serious		5
**Mitral Valve Prolapse and other Cardiovascular Complications**													
Cardiovascular abnormalities in children with autosomal dominant polycystic kidney disease<br><br>(PMID 7579051)	Ivy, DD	JASN 5: 2032-2036	1995	Observational study	83 children affected by ADPKD from 154 children at risk of ADPKD, mean age 9.6 years	10/79 (12%) had mitral valve prolapse	3% of children not affected had mitral valve prolapse	Not serious	Not serious	Not serious	Not serious		6
Increased left ventricular mass in children with autosomal dominant polycystic kidney disease and borderline hypertension<br><br>(PMID 18716604)	Cadnapaphornchai, M	Kidney Int 74: 1192-1196.	2008	Observational study	85 children and adolescents with ADPKD (aged 4-21yrs) recruited from ongoing ADPKD cohort in Denver	LVMI significantly correlated with SBP ($p<0.0001$) and DBP ($p<0.007$)	Children with ADPKD with borderline BP and high BP had a significantly higher LVMI than children with ADPKD with normal BP.	Not serious	Not serious	Not serious	Not serious		7

Title	First Author	Journal	Year	Study design	Nº & type of patients	Incidence	Details	Risk of bias	Indirectness	Inconsistency	Imprecision	Other considerations	Importance (1-9)
Genotype-phenotype correlation in children with autosomal dominant polycystic kidney disease<br><br>(PMID 19194729)	Fencl, F	Pediatr Nephol 24: 983-989	2009	Retrospective note review	60 children with PKD and confirmed genetic testing from cohort of 260 children with ADPKD	1/60 (2%) had a mitral valve prolapse	Study split into 50 children with PKD1 and 10 children with PKD2	Serious – small single centre study in specialized centre	Not serious	Not serious	Not serious		6
**Inguinal hernia**													
Spectrum of autosomal dominant polycystic kidney disease in children<br><br>(PMID 8011974)	Fick, GM	JASN 4: 1654-1660	1994	Prospective observational study	62 children affected by ADPKD from 140 children at 50% risk of ADPKD	6/62 (10%)	Proportion of children requiring surgery for inguinal hernia	Not serious	Not serious	Not serious	Not serious		7
Autosomal-dominant polycystic kidney in infancy and childhood: Progression and outcome<br><br>(PMID 16221221)	Shamshirsaz, A	Kidney Int 68: 2218-2224	2005	Observational study	199 children with ADPKD, recruited from cohort of families at risk	16/199 (8%)	Proportion with history of inguinal hernia<br><br>Study population split into 46 children with VEO ADPKD (<18 months) and 153 children with onset 18mo-18yrs<br><br>Significantly more risk of inguinal hernia in VEO group compared to non-VEO ($p<0.01$)	Not serious	Not serious	Not serious	Not serious		7

Abbreviations: ADPKD: autosomal dominant polycystic kidney disease. ARPKD: autosomal recessive polycystic kidney disease. DBP: diastolic blood pressure. ESRD: end stage renal disease. GFR: glomerular filtration rate. GH: glomerular hyperfiltration. LVMI: left ventricular mass index. PKD: polycystic kidney disease. SBP: systolic blood pressure. USS: ultrasound scan. UTI: urinary tract infection. VEO: very early onset.

Tables S5 Literature reviews on hypertension in ADPKD

Tables S5a Incidence of hypertension in children and adolescents with ADPKD

Title	First Author	Journal	Year	Study design, patients	Hypertension measure	Prevalence of hypertension	Details	Risk of bias	Indirectness	Inconsistency	Imprecision	Other con-siderations	Importance (1-9)
**Overall**													
Hypertension in autosomal dominant polycystic kidney disease: a meta-analysis<br><br>PMID: 27288429	Marlais, M.	Arch Dis Child 101: 1142-1147	2016	Systematic review and meta-analysis.<br>928 children across 14 studies	Very variable. Only one study with 24h ABPM.	20%<br>(95%CI 15-27%)	Metaanalysis:<br>Significant meta-regression of mean participant age in study and prevalence of hypertension (p=0.0128).<br><br>Mean patient age 4 years: prevalence ~ 10%<br>Mean patient age 16 years: prevalence ~ 36%	Not serious	Not serious	Not serious	Not serious	High degree of methodological heterogeneity noted across studies	8
**Subgroups**													
**Very early onset** (VEO = diagnosis before age 18 months)													
Long-term outcomes in patients with very-early onset autosomal dominant polycystic kidney disease<br><br>PMID: 27548646	Nowak, K.L.	Am J Nephrol 44: 171-178	2016	Longitudinal retrospective cohort study.<br>70 VEO and 70 age- and sex-matched non-VEO ADPKD children.	Repeated oscillometric measures in most patients, but it ~n=20 only by questionnaire.	42/70 (61%) in VEO vs 21/70 (30%) in non-VEO patients (p<0.0001)	VEO patients significantly more likely to develop hypertension than non-VEO patients (61% in VEO and 30% in non-VEO group at last follow-up, HR: 3.15 [1.86–5.34], p<0.0001).<br><br>VEO patients developed hypertension significantly earlier (median age 20 vs 38 years, p<0.001).	Serious – possible age above 18yrs	Not serious	Serious – possible due to higher median age and selected (VEO) population	Not serious	Note that some individuals in this study will be >18yrs at last follow up	7
Autosomal-dominant polycystic kidney disease in infancy and childhood: progression and outcome.<br><br>PMID: 16221221	Shamshirsaz, A.A.	Kidney Int. 68(5):2218-24.	2005	Longitudinal retrospective cohort study.<br>46 VEO and 153 non-VEO ADPKD children.	Clinic BP measurements	52% in VEO vs<br>32% in non-VEO patients (p <0.05)	Comparisons between VEO and non-VEO children at the most recent follow-up demonstrated significantly more hypertension in non-VEO children diagnosed due to signs or symptoms versus non-VEO children diagnosed due to screening (VEO 60% vs. 50%, P = NS; non-VEO 59% vs. 23%, P < 0.0001).						
**Children identified via screening vs with presenting symptoms**													
Similar renal outcomes in children with ADPKD diagnosed by screening or presenting with symptoms.<br><br>PMID: 20683618	Mekahli, D.	Pediatr Nephrol. 25(11):2275-82	2010	Retrospective cohort study.<br>31 ADPKD children identified by asymptomatic screening vs 16 through presenting symptoms	Clinic BP measurements.<br><br>Hypertensive: 3 measurements > 95th pctl<br>Borderline: 95th to 95th pctl	Prevalence of hypertension (diagnosis → last FU):<br>Screening: 2/31 (6%) → 5/31 (16%)<br>Symptomatic: 1/16 (6%) → 2/16 (13%),<br>p= ns between groups	Borderline hypertension (diagnosis → last FU):<br>Screening: 0 → 4/31 (13%)<br>Symptomatic: 0 → 3/16 (19%), p= ns<br>SBP index (diagnosis → last FU):<br>Screening: 0.85 ± 0.03 → 0.84 ± 0.02<br>Symptomatic: 0.86 ± 0.04 → 0.88 ± 0.04, p=ns<br>DBP index (diagnosis → last FU):<br>Screening: 0.78 ± 0.02 → 0.78 ± 0.02<br>Symptomatic: 0.79 ± 0.02 → 0.79 ± 0.02, p=ns						

Title	First Author	Journal	Year	Study design, patients	Hypertension measure	Prevalence of hypertension	Details	Risk of bias	Indirectness	Inconsistency	Imprecision	Other considerations	Importance (1-9)
Autosomal-dominant polycystic kidney disease in infancy and childhood: progression and outcome.<br><br>PMID: 16221221	Shamshirsaz, A.A.	Kidney Int. 68(5):2218-24.	2005	Longitudinal retrospective cohort study.<br>46 VEO and 153 non-VEO ADPKD children.	Clinic BP measurements	VEO group:<br>Screening: 50%<br>Symtpomatic: 60% (p=ns)<br>Non-VEO group:<br>Screening: 23%<br>Symtpomatic: 59% (p <0.0001)	Prevalence of hypertension at last visit in patients identified by screening vs by presenting symptoms was different in non-VEO group, but not in VEO group.						
**Genotype**													
Genotype-phenotype correlation in children with autosomal dominant polycystic kidney disease.<br><br>PMID: 19194729	Fencl, F.	Pediatr Nephrol. 24(5):983-9	2009	Cross sectional study.<br>50 children with PKD1 and 10 with PKD2 mutations.	All have clinic BP measurements, and 38 ABPM (n=33 PKD1 and n=5 PKD2)	10/50 (20%) PKD1 vs 3/10 (30%) PKD2 (p=ns) by clinic BP.<br><br>9/33 (27%) PKD1 vs 0/5 (0%) PKD2 (p=ns) by ABPM criteria.	Clinic BP index: no difference between groups.<br><br>ABPM: systolic index significantly different both for day-time (0.93 ± 0.1 vs 0.86 ± 0.05, p=0.02) and night-time periods (0.94 ± 0.07 vs 0.89 ± 0.04, p=0.037). No difference in diastolic BP index or night-time dips.						

Table S5b Correlation of blood pressure to outcome in children with ADPKD (excluding controlled trials)

Title	First Author	Journal	Year	Study design, patients	Hypertension measure	Disease marker	Findings	Risk of bias	Indirectness	Inconsistency	Imprecision	Other considerations	Importance (1-9)
**Correlation of Blood Pressure to renal/cyst volume in ADPKD children**													
Magnetic resonance imaging of kidney and cyst volume in children with ADPKD.<br><br>PMID: 21115621	Cadnapaphornchai, M.A.	Clin J Am Soc Nephrol. 6(2):369-76	2011	Cohort study.<br><br>77 children with ADPKD, aged 4 -21 years	Repeated clinic BP measurements:<br>Hypertensive (HBP): BP ≥ 95th percentile,<br>Normotensive (NBP): BP<95th pctl. for age, height, and gender.	TKV and cyst volume on annual MRI for 5 years	TKV and total cyst volume were significantly increased in HBP versus NBP subjects (p<0.0001 and p<0.005).<br>Fractional cyst volume was similar at baseline, but higher in HBP subjects starting from year 2 until end of study (p=0.02).<br><br>Cyst number was similar at baseline, but higher in HBP children from year 3 until end of study (p<0.01).						
Increased left ventricular mass in children with autosomal dominant polycystic kidney disease and borderline hypertension.<br><br>PMID: 18716604	Cadnapaphornchai, M.A.	Kidney Int. 74(9):1192-6	2008	Cohort study (baseline in RCT).<br><br>85 children with ADPKD, aged 4-21 years.<br>All had normal renal function.	Repeated clinic BP measurements:<br>3 groups:<br>Hypertensive (HBP): BP≥95th percentile.<br>Borderline (BBP): BP 75th-95th pctl.<br>Normotensive (NBP): BP < 75th pctl	Kidney volumes (mean of both sides) on 2D US (ellipsoid formula).<br><br>Cyst number recorded up to 15 per kidney.	Correlations in whole group:<br>-systolic BP and log renal volume (r=0.70, P<0.0001)<br>-diastolic BP and renal volume (r=0.52, P<0.0001)<br><br>Multiple linear regression (covariates age, height, sex) confirmed significant predictive value of systolic BP on log of renal volume (P=0.0002), but diastolic BP was not significant (P=0.2).<br><br>HBP children had larger renal volume than BBP an NBP groups (both p<0.002).<br>Very similar renal volume in NBP and BBP groups.						
Ambulatory blood pressure correlates with renal volume and number of renal cysts in children with autosomal dominant polycystic kidney disease.<br><br>PMID: 12900587	Seeman, T.	Blood Press Monit. 8(3):107-10.	2003	Cross sectional cohort study.<br><br>62 children with ADPKD, aged 3.4 to 19.4 (mean age 12.3 ± 4.3 years).<br>Normal renal function	24h ambulatory BP monitoring (ABPM):<br>n=22 hypertensive (untreated),<br>n= 40 normotensive.	Kidney volumes (mean of both sides) on 2D US (ellipsoid formula).<br><br>Cyst number in both kidneys combined.	Renal volume was significantly greater in hypertensive than in normotensive children (2.7 ± 2.3 SDS versus 1.2 ± 2.5 SDS, P<0.01) despite similar anthropometric data and renal function.<br>Mean number of cysts was significantly higher in hypertensive patients than in normotensive (35 ± 15 cysts versus 23 ± 14 cysts, P<0.01).<br><br>Renal volume correlated with daytime as well as with night-time systolic and diastolic BP (r=0.41-0.47, P<0.01). Less correlation with renal length and the number of renal cysts (r=0.29-0.43, P<0.05 and 0.01).						
Progression of autosomal-dominant polycystic kidney disease in children.<br><br>PMID: 11318935	Fick-Brosnahan, G.M.	Kidney Int. 59(5):1654-62.	2001	182 ADPKD children from 131 families.<br><br>(n= 108 with ≥ 1 follow-up, of which n=30 have early severe disease).	Repeated clinic BP measurements:<br>High blood pressure defined ≥ 50% systolic or diastolic measurements ≥75th pctl. for age-, gender-, and height-matched children, or being on antihypertensive therapy.	Kidney volumes (mean of both sides) on 2D US (ellipsoid formula).<br><br>Group comparisons normalized to height.	ADPKD children with high blood pressure at first and last visit (n=33) had faster renal growth than those with blood pressure < 75th pctl at 1st and last visit (n=37 p<0.01) (but they also had higher baseline renal volumes p<0.0001 and renal growth adjusted for baseline renal volume did not differ).<br><br>Hypertension was associated with a larger cyst diameter (age-adjusted 3.1 ± 0.2 cm vs. 2.0 ± 0.1 cm, P < 0.0001), but this was not independent from the effects of larger mean renal volumes.	Bias towards children with more follow-up visits in longitudinal analysis				Even BP 75th to 95th pctl. defined as hypertension. (only n=17 had BP > 95th pctl)	

Title	First Author	Journal	Year	Study design, patients	Hypertension measure	Disease marker	Findings	Risk of bias	Indirectness	Inconsistency	Imprecision	Other considerations	Importance (1-9)
**Correlation of Blood Pressure to eGFR in ADPKD children**													
Ambulatory blood pressure correlates with renal volume and number of renal cysts in children with autosomal dominant polycystic kidney disease.<br><br>PMID: 12900587	Seeman, T.	Blood Press Monit. 8(3):107-10.	2003	Cross sectional cohort study.<br><br>62 children with ADPKD, aged 3.4 to 19.4 (mean age 12.3 ± 4.3 years).	24h ambulatory BP monitoring (ABPM): n=22 hypertensive (> 95th pctl., all untreated), n= 40 normotensive.	Creatinine clearance estimation (Schwartz formula)	No significant difference in Schwartz clearance between normotensives 111 ± 15 vs hypertensives 115 ± 16 ml/min*1.73m^2.					Because glomerular hyperfiltration preceeds CKD, eGFR is a poor marker in early ADPKD	
Progression of autosomal-dominant polycystic kidney disease in children.<br><br>PMID: 11318935	Fick-Brosnahan, G.M.	Kidney Int. 59(5):1654-62.	2001	182 ADPKD children (which n=30 have early severe disease) and 127 unaffected siblings.	Repeated clinic BP measurements: High blood pressure defined as ≥ 50% systolic or diastolic measurements ≥75th pctl., or being on antihypertensive therapy.	Creatinine clearance estimation (Schwartz formula) at last follow-up	Mean Schwartz GFRs at last visit (all ml/min/1.73m^2): similar in BP > 75th vs < 75th pctl (133 vs. 137) (also in affected vs unaffected children (135 vs.129), and in early severe vs milder disease (132 vs. 136)).					Because glomerular hyperfiltration preceeds CKD, eGFR is a poor marker in early ADPKD	
**Correlation of Blood Pressure to proteinuria in ADPKD children**													
Hypertension in autosomal dominant polycystic kidney disease: a meta-analysis<br><br>PMID: 27288429	Marlais, M.	Arch Dis Child 101: 1142-1147	2016	Systematic review and meta-analysis. 928 children across 14 studies	Very variable. Only one study with 24h ABPM.	Proteinuria on quantitative measurement.	Metaanalysis:<br>Meta-regression of prevalence of hypertension in a study with prevalence of proteinuria shows no evidence of a relationship ($p=0.2641$).	Not serious	Not serious	Not serious	Not serious	High degree of methodological heterogeneity noted across studies	
**Correlation of Blood Pressure to urinary concentrating ability in ADPKD children**													
Renal concentrating capacity is linked to blood pressure in children with autosomal dominant polycystic kidney disease.<br><br>PMID: 15588131	Seeman, T.	Physiol Res. 53(6):629-34.	2004	53 children with ADPKD (mean age 11.8+/-4.4 years)	24h ambulatory BP monitoring (ABPM) one day before desmopressin.	Standardized renal concentrating capacity test: nasal drop application of desmopressin	Renal concentrating capacity was decreased in 58 % of children. Prevalence of hypertension was significantly higher in children with decreased renal concentrating capacity (35 %) vs with normal renal concentrating capacity (5 %) ($p<0.05$).<br>Significant negative correlations between renal concentrating capacity, ambulatory BP and number of renal cysts ($r = -0.29$ to -0.39, $p<0.05$ to $p<0.01$).	Not serious	Not serious	Not serious	Not serious	Urinary concentrating ability is not a well-established disease marker.	

Table S5c Controlled trials of antihypertensive treatments in ADPKD

Title	First Author	Journal	Year	Study design	Nº & type of patients	Details of treatement & outcome measures	Findings	Risk of bias	Indirectness	Inconsistency	Imprecision	Other con-siderations	Importance (1-9)
**Systematic Review and Metaanalysis**													
Interventions for preventing the progression of autosomal dominant polycystic kidney disease<br><br>(PMID 26171904)	Bolignano, D	Cochrane Database Syst Rev 7:CD010294	2015	Systematic Review	30 studies (involving 2039 participants) that tested 11 different treatments in ADPKD<br><br>For those with BP data:<br><br>eGFR ranged from 38.2 to 124 mL/min in adult ADPKD patients;<br><br>102 to 142 mL/min in children	**Primary outcome**: SCr (mg/dL), measured or estimated GFR (eGFR) (mL/min or mL/min/1.73 m2), creatinine clearance (CrCl), doubling of creatinine, need for RRT or transplantation at the end of treatment<br><br>**Secondary outcome**: Blood pressure (BP): systolic BP and diastolic BP (mm Hg), mean BP (mm Hg	ACEi significantly reduced diastolic blood pressure (9 studies, 278 participants: MD -4.96 mm Hg, 95% CI -8.88 to -1.04), but had uncertain effects on kidney volumes, GFR and SCr in data largely restricted to children.<br>Data for calcium channel blockers were sparse and inconclusive.<br><br>ACEi versus no treatment [n=42]<br>systolic BP -5.44 [-14.26, 3.38; I^2 = 96%]<br>diastolic BP -4.96 [-8.88, -1.04; I^2 = 90%]<br><br>ACEi versus no treatment [n=61]<br>MAP -5.0 [-6.29, -3.71]<br><br>ACEi versus CCB [n=24]<br>systolic BP -5.0 [-8.62, -1.38]<br>diastolic BP -3.0 [-5.40, -0.60]<br>MAP -3.0 [-5.40, -0.60]<br><br>ACEi versus ARB [n=32]<br>systolic BP -3.5 [-9.75, 2.75]<br>diastolic BP -1.80 [-5.23, 1.63]<br>MAP -2.20 [-6.41, 2.01]<br><br>ACEi versus Beta blocker [n=37]<br>systolic BP -1.0 [-2.29, 0.29]<br>diastolic BP 1.0 [0.35, 1.65]<br>MAP -3.0 [-4.92, -1.08]	Not serious				Random sequence generation in one study only;<br><br>blinding was not present or not specified<br><br>>10% dropout in 3 studies<br><br>Extreme heterogeneity in study length;<br><br>none conducted using intention-to-treat analyses.	6
Antihypertensive treatments in adult autosomal dominant polycystic kidney disease: network meta-analysis of the randomised controlled trials<br><br>(PMID 26636542)	Xue, C	Oncotarget 6(40):42515-29	2015	Network meta-analysis	10 RCTs with 1386 patients	Six interventions: ACEI, ARB, combination of ACEI and ARB, CCB, β-blockers and dilazep	No difference of eGFR in all the treatments in both network and direct comparisons<br><br>No significant differences of Scr, SBP, DBP, MAP, and LVMI were found in network comparisons<br><br>Little evidence to detect differences of antihypertensive treatments on kidney disease progression in ADPKD patients<br><br>ARB may be an optimal choice in clinical practice					Unable to comment on methods	

Title	First Author	Journal	Year	Study design	Nº & type of patients	Details of treatement & outcome measures	Findings	Risk of bias	Indirectness	Inconsistency	Imprecision	Other con-siderations	Importance (1-9)
**Trials in Children**													
Prospective change in renal volume and function in children with ADPKD<br><br>(PMID 19346430)	Cadnapaphornchai, MA	Clin J Am Soc Neph 4(4):820-9	2009	Prospective randomized study - 3 subgroups of ADPKD<br><br>HTN (BP >or= 95th percentile)<br><br>BBP (BP 75 to 95th percentile)<br><br>SPKD (BP <or=75th percentile with > 10 renal cysts)	85 ADPKD with normal renal function<br><br>Mean age 14±1 [HTN]<br><br>12±1 [BBP]<br><br>12±1 [SPKD]	HTN n=28<br>BBP n=27<br>SPKD n=30<br><br>HTN randomised to two arms [BP<90th vs. <50th]<br><br>BBP randomised to two arms [BP<50th with ACEi vs. < 95th but no ACEi]<br><br>SPKD randomised to two arms [BP<50th with ACEi vs. < 75th but no ACEi]<br><br>**Primary outcome** Renal volume by ultrasound<br><br>**Secondary outcome** left ventricular mass index (LVMI) and microalbuminuria.<br><br>Secondary analysis compared hypertensive and normotensive groups.<br><br>**Follow-up** 5 years	Average change in renal volume per year across all groups was 32±10 ml/1.73 m^2/y or 9.3±3.3 percent/1.73m^2/y<br><br>In HTN [BP<90th vs. <50th]:<br>- both groups demonstrated increased renal volume over time<br>- BP indices reduced and maintained over 5-year study period<br>- <50th vs. <90th percentile BP group required more medications to meet target [2.8 vs. 1.6, P <0.05]<br>- both groups showed no significant difference between baseline and year 5 for LVMI and microalbuminuria<br><br>In BBP [BP<50th with ACEi vs. < 95th but no ACEi]:<br>- both groups demonstrated increased renal volume over time but<br>- no significant treatment effect in either arm on renal volume by ultrasound, LVMI, and microalbuminuria<br>- an increase in LVMI 66 [59–74] versus 77 [67–88] g/m2, P <0.05) from baseline to year 5 and a reduction in renal function in the BBP with < 95th but no ACEi arm<br><br>In SPKD [BP<50th with ACEi vs. < 75th but no ACEi]:<br>- both groups demonstrated increased renal volume over time<br>- no significant treatment effect in either arm on renal volume by ultrasound, LVMI, and microalbuminuria<br>- both groups demonstrated an increase in LVMI over time<br><br>HTN (n=28) versus NTN (n=57) [BP>95th vs. < 95th]:<br>- HTN consistently had higher renal volume than normotensive children throughout the 5-yr study period<br>- HTN also had higher LVMI at baseline and tended to have higher LVMI throughout the study<br>- HTN showed a significant decrease in renal function	Not serious				No power calculation as no prior data<br><br>High dropout rate to completion<br><br>From Cochrane review:<br>Systolic BP: MD - 5.44 mm Hg, 95% CI -14.26 to 3.38; I2 = 96%)<br><br>Diastolic BP -4.96 [-8.88, -1.04; I^2 = 90%]<br><br>.	7

Title	First Author	Journal	Year	Study design	Nº & type of patients	Details of treatement & outcome measures	Findings	Risk of bias	Indirec tness	Inconsist ency	Imprec ision	Other con-siderations	Import ance (1-9)
**Trials in Adults**.													
**Level of BP in ADPKD**													
Cardiac and renal effects of standard versus rigorous blood pressure control in autosomal dominant polycystic kidney disease: results of a seven-year prospective randomized trial<br><br>(PMID 12089368)	Schrier, R	J Am Soc Nephrol 13(7):1733-9	2002	prospective, randomized study	75 ADPKD patients with established hypertension and left ventricular hypertrophy (LVH) recruited between 1991 -1994<br><br>Mean age 41 (21-59) years	To examine the effect of rigorous (<120/80 mmHg) versus standard (135-140/85-90 mmHg) BP control on left ventricular mass index (LVMI) and kidney function<br><br>**Primary outcome** LVMI at baseline and at 1 and 7 yr. Renal function assessed<br><br>**Follow up**: 7 years	Average MAP during the study was 90 ±5 mmHg for the rigorous group and 101 ± 4 mmHg for the standard group (P <0.0001)<br>LVH decreased to a significantly greater extent by rigorous than standard BP control<br>The LVMI decreased by 21% in the standard group and by 35% in the rigorous group.<br>- rigorous BP control was significantly more effective in decreasing LVMI (P < 0.01)<br>- rigorous BP control was demonstrated to be particularly important for male ADPKD patients with LVH<br>- significant interaction between BP control group and drug over time (P < 0.005); enalapril better at reducing LVMI than amlodipine<br>On average, 1.4 ± 0.6 drugs were needed in the standard group and 2.7 ± 0.8 in rigorous control group (P 0.0001).<br>No statistically significant difference in renal function between the two BP groups<br>- Time to ESRD was similar standard (3.2 ±1.8 yr) and rigorous (4.0 ± 1.4 yr) (P =NS)<br>- no significant difference between enalapril and amlodipine on the 24-h creatinine clearance over time	Not serious				Randomized to either enalapril or amlodipine initially but randomization terminated after mean of 2.1 years as funding lost.<br><br>69 subjects [20 on amlodipine and 49 enalapril] who were on one of the two drugs for at least 80% of their study time had a sub-group analysis.	5
Blood pressure in early autosomal dominant polycystic kidney disease<br><br>(PMID 25399733)<br><br>HALT-PKD Study A	Schrier, R	N Engl J Med. 371(24):2267-76.	2014	Double-blind, placebo-controlled randomized controlled trial<br><br>Randomised to 1 of 4 arms (2-by-2 design)	558 ADPKD patients<br><br>15 to 49 years of age<br><br>eGFR > 60 ml/min/1.73 m²	Standard BP (n=284) [ACEi + ARB therapy, n=144; ACEi + Placebo, n=140]<br><br>Low BP (n=274) [ACEi + ARB therapy, n=133; ACEi + Placebo, n=141]<br><br>ACEi + ARB vs ACEi alone at 2 levels of BP control [standard BP target (120/70 to 130/80 mm Hg) or a low BP target (95/60-110/75 mmHg)]<br><br>**Primary outcome**: Annual % change in Total kidney Volume<br><br>**Follow up**: 5 to 8 years	Lower BP target had a significant reduction of the kidney volume growth (5.6 vs 6.6%, P = 0.006).<br>- the combination of ACEi + ARB therapy was no better than ACEi alone in reducing TKV growth<br><br>Lower BP target compared to higher BP target had<br>- significant reduction in left-ventricular-mass index (-1.17 vs. -0.57 g/m2/year, P<0.001)<br>- significant reduction in urinary albumin excretion (3.77% vs. 2.43%, P<0.001)<br>- no difference in the level of renal function (GFR annual loss -2.9 vs -3.0 ml/min/1.73 m2)<br>ACEi + ARB therapy compared with ACEi alone<br>- no difference in the growth of kidney volume, GFR loss, albuminuria and LVMI<br><br>Dizziness and light-headedness were more common in the low-blood-pressure group than in the standard-blood-pressure group (80.7% vs. 69.4%, P = 0.002)<br>Measured BP level difference between two BP groups was 13.4/9.3 mmHg [difference in home BP at 96 months]; with median 2.0 anti-HTN in Low versus 1.0 in Standard BP target group<br>SBP/DBP level on target 40-66%/58-75% in Low BP versus 32-48%/33-52% in Standard BP target group	Not serious	Not serious	Not serious	Not serious	No control arm without inhibitors of the RAAS considered but thought to be ethically questionable	9

Title	First Author	Journal	Year	Study design	Nº & type of patients	Details of treatement & outcome measures	Findings	Risk of bias	Indirectness	Inconsistency	Imprecision	Other con-siderations	Importance (1-9)
**ACE vs ACE & ARB**													
Angiotensin Blockade in Late Autosomal Dominant Polycystic Kidney Disease<br><br>(PMID 25399731)<br><br>HALT-PKD Study B	Torres, VE	N Engl J Med. 371(24):2267-76.	2014	Double-blind, placebo-controlled trial	486 ADPKD<br><br>18 to 64 years of age<br><br>GFR 25 to 60 mL/min/1.73 m^2	ACE inhibitor (lisinopril) and placebo or lisinopril and an ARB (telmisartan) with the doses adjusted to achieve a blood pressure of 110/70 to 130/80 mm Hg.<br><br>**Primary outcome** composite of time to death, end-stage renal disease, or a 50% reduction from the baseline estimated GFR.<br><br>**Follow up**: 5 to 8 years.	No difference in the incidence of the composite primary outcome (hazard ratio with lisinopril-telmisartan, 1.08; 95% confidence interval, 0.82 to 1.42)<br><br>No difference in secondary end points including GFR decline, albuminuria and adverse events like hyperkalemia and acute kidney injury<br><br>Monotherapy with an ACE inhibitor was associated with blood-pressure control in most patients with ADPKD and stage 3 chronic kidney disease.<br><br>ACEi + Placebo compared with ACEi + ARB had higher SBP 1.23 mmHg [0.24 – 2.21, P=0.02]; MAP 0.89 mmHg [0.15 – 1.63, P=0.02]<br><br>SBP level on target 73-86%; MAP level on target 70-83% and DBP 50-65% during 72 months of follow-up<br><br>Dose of ACEi was lower in Dual blockade compared to ACEi monotherapy alone but<br>- both treatments controlled blood pressure and lowered urinary aldosterone excretion similarly<br>- those with ACEi + Placebo received diuretics and beta blockade more often	Not serious	Not serious	Not serious	Not serious	No control arm without inhibitors of the RAAS considered but thought to be ethically questionable	9
**ACEi versus Beta blockers**													
Renal and cardiac effects of antihypertensive treatment with ramipril vs metoprolol in autosomal dominant polycystic kidney disease<br><br>(PMID 17984104)	Zeltner, R	Nephrol Dial Transplant 23(2):573-9	2008	prospective randomized double-blind study	46 hypertensive ADPKD adults<br><br>Mean age: 40.7±2.2 years [Ramipril] vs 40.0±2.2 years [Metoprolol]	Ramipril (n = 23) or Metoprolol (n = 23).<br><br>**Primary outcome** Twenty-four hour (24-h) ambulatory blood pressure (BP), glomerular filtration rate (eGFR) urinary albumin excretion (albumin/creatinine ratio), and left ventricular mass index (LVMI)<br><br>**Follow-up** 3 years	After the 3 years follow-up, no differences in renal function, urinary albumin excretion and LVMI were detected between those treated with ramipril or metoprolol<br><br>*Post-hoc* analysis, the effects of rigorous vs standard BP control [MAP </> 97mmHg by ABPM] were evaluated irrespective of the study medication<br><br>LVMI increased in patients with standard BP control while it remained stable in patients with rigorous BP control with significant difference in LVMI between groups after 3 years (110.5 ± 6.3 vs 90.9 ± 4.7 g/m^2; P = 0.017).<br><br>At 3 years albuminuria was lower in patients with rigorous vs standard BP control (23.5 ± 6.7 vs 94.8 ±35.4 mg/g; P = 0.05).	Not serious				Single centre<br><br>Not blinded<br><br>Shorter duration of follow up<br><br>Higher dropout rate in males	4

Title	First Author	Journal	Year	Study design	Nº & type of patients	Details of treatement & outcome measures	Findings	Risk of bias	Indirectness	Inconsistency	Imprecision	Other con-siderations	Importance (1-9)
No effect of enalapril on progression in autosomal dominant polycystic kidney disease<br><br>(PMID 14551359)	Van Dijk, MA	Nephrol Dial Transplant 18(11):2314-20	2003	Prospective Randomised study- 2 subgroups of ADPKD<br><br>Normotensive [NTN] double-blind placebo-controlled study<br><br>Hypertensive [HTN] randomised Enalapril or Atenolol	61 of 72 randomised NTN<br><br>Mean age 36±2 vs 37±2 years<br><br>28 of 35 randomised HTN<br><br>40±3vs 33±3 years [Enalapril vs. Atenolol]	NTN group treated with Enalapril or Placebo to achieve diastolic blood pressure decrease >10mmHg, or below 70mmHg<br><br>HTN group randomised to using Enalapril or Atenolol to achieve BP level <140/85 mmHg<br><br>**Primary outcome** Effect on renal function, blood pressure and microalbuminuria<br><br>**Follow-up** 3 years	No beneficial effect of ACE inhibition demonstarted on loss of renal function in ADPKD patients with normotension or hypertension<br><br>Normotensive group blood pressure decreased with enalapril versus placebo (-3±2 vs. 2±2 mmHg, P=NS), it had no effect on microalbuminuria.<br><br>Hypertensive group, antihypertensive treatment significantly decreased MAP -7±2mmHg (P<0.01) from baseline<br>- reduction in MAP Enalapril versus Atenolol -11±3 vs –3±3mmHg, P=NS<br>- those on atenolol required more additional treatment to control blood pressure but similar microalbuminuria	Not serious				Single centre<br><br>Shorter duration of follow up<br><br>Low maximal dose of Enalapril	4
**ACEi or ARB versus CCB**													
Effect of antihypertensive therapy on renal function and urinary albumin excretion in hypertensive patients with autosomal dominant polycystic kidney disease.<br><br>(PMID 10692268)	Ecder, T	Am J Kidney Dis 35(3):427-32	2000	Prospective, randomized study	24 hypertensive ADPKD<br><br>Mean Ccr 83 vs 77 [Aml vs Enal]<br><br>Mean age 43years [Amlodipine]<br><br>41 years [Enalapril]	Amlodipine (n=12) Enalapril (n = 12)<br><br>**Primary outcome** blood pressure, renal function, and urinary albumin excretion<br><br>**Follow up** 5 years	Blood pressure similar in both groups and showed significant change from baseline but no difference in BP level between groups [Aml vs Enal]<br>- Baseline MAP 109 vs 108 mmHg<br>- 1-year 96 vs 89 mmHg<br>- 5-year 97 vs 94 mmHg<br><br>Annual decline in GFR in all patients was 3.4 mL/min/1.73 m^2 with no difference between Aml vs Enal [2.8 vs 4.2 mL/min/1.73 m^2, P=NS]<br><br>Enalapril had significant effect to sustain decreased microalbuminuria over 5-year follow-up	Serious				Not blinded<br><br>Different numbers recieved additional anti-hypertensives in each group, therefore not fully comparable at follow up.	3
Calcium channel blocker versus angiotensin II receptor blocker in autosomal dominant polycystic kidney disease<br><br>(PMID 15637459)	Nutahara, K	Nephron Clin Pract 99(1):c18-23	2005	Prospective, randomized study	49 hypertensive ADPKD<br><br>Mean Ccr 71.9 vs 69.8 [Aml vs Can]<br><br>Mean age 48.4 years [Amlodipine]<br><br>47.3 years [Candesartan]	Amlodipine (n=25) Candesartan (n = 24)<br><br>**Primary outcome** renal function, urinary protein and albumin excretion<br><br>**Follow-up** 36 months (median)	Baseline characteristics were similar, and blood pressure was well controlled in both groups throughout the study period..<br>- values of BP shown in figure but not stated<br><br>Renoprotective effect of candesartan >amlodipine, independent of the level of BP<br>- Serum creatinine higher in Amlodipine group at 24 and 36 months<br>- decrease in GFR higher in Amlodipine vs. Candesartan at 36 months (Delta -20.9 +/- 13.1 vs. -4.8 +/- 13.8 ml/min, p < 0.01).<br>- Microalbuminuria lower in candesartan at 12, 24 and 36 months.	Serious				Not blinded<br><br>Different numbers recieved additional anti-hypertensives in each group, therefore not fully comparable at follow up.	3

Title	First Author	Journal	Year	Study design	Nº & type of patients	Details of treatement & outcome measures	Findings	Risk of bias	Indirectness	Inconsistency	Imprecision	Other con-siderations	Importance (1-9)
**ACEi versus ARB**													
A comparison of the effects of ramipril and losartan on blood pressure control and left ventricle hypertrophy in patients with autosomal dominant polycystic kidney disease<br><br>(PMID 20722556)	Ulusoy, S	Ren Fail 32(8):913-7	2010	Prospective, randomized study	32 hypertensive ADPKD<br><br>Mean age 51.4±10.3 [Losartan]<br><br>47.7±7.4 [Ramipril]	Losartan (n=19)<br>Ramipril (n = 13)<br><br>**Primary outcome** Effects on blood pressure (BP) control, LVH, and renal progression<br><br>**Follow-up** 12 months	Effective BP control [156.3 ± 15.7 (L) vs. 150.00 ± 19.6 (R) mmHg] to [116.6 ± 8.5 (L) vs. 120 ± 9.2 (R) mmHg] at the end of the first year (p < 0.001).<br><br>LVH was found to be regressed significantly in the hypertensive patients with ADPKD	Very serious				Small study, no power calculation, why 13:19 split across group (should be 16:16 if truly randomised)<br><br>Not blinded<br><br>No difference between the 2 drugs found, but likely due to lack of power	3
Effect of dilazep dihydrochloride on urinary albumin excretion in patients with autosomal dominant polycystic kidney disease.<br><br>(PMID 11340355)	Nakamura, T	Nephron 88(1):80-2	2001	prospective, randomized study	12 NTN & 10 HTN ADPKD	Dilazep dihydrochloride, an antiplatelet drug	No reported effect on blood pressure<br><br>dilazep dihydrochloride may be effective in reducing UAE in normotensive ADPKD patients with microalbuminuria	Very serious				Small study<br><br>Not blinded	1

Tables S6 Literature review on Non-pharmacological treatments to slow the progression of ADPKD

Table S6a Water intake and progression of ADPKD

Title	First Author	Journal	Year	Study design	Nº & type of patients	Intervention/ Group difference	Outcome results	Risk of bias	Indirectness	Inconsistency	Imprecision	Other considerations	Importance (1-9)
**Interventional studies (of high water intake on disease progression in ADPKD (GFR/TKV))**													
Does increased water intake prevent disease progression in autosomal dominant polycystic kidney disease?<br><br>PMID: 24739484	Higashihara, E.	Nephrol Dial Transplant 29(9):1710-9	2014	Interventional controlled trial (groups allocated mainly on patient preference)	34 patients with ADPKD age 20-65 y, eGFR > 50 ml/min/1.73m²	Interventional group= high water intake n=18; control group = free water intake N = 16	Primary outcome: TKV and kidney function slopes<br>- No difference between high- and free- intake group.<br>- Compared to pre-study, TKV and kidney function slopes became worse (P = 0.047 and 0.011, respectively) after high water intake (H-group) but not in the F-group<br>Secondary outcomes:<br>- urine sodium and urine solute excretion were higher and urine osmolality, plasma osmolality and plasma sodium were lower in the high intake -group.<br>- Urine volume: urine volume increased significantly more in high-intake group compared to free-intake group (p=0.001)<br>- Plasma copeptin lower in high intake group than free intake group(p=0.024)<br>- eGFR: no sgnificant difference between groups<br>>> no benefit of high water intake demonstrated. May be even detrimental.	Small cohort. Group allocation on patient preference = not randomized. High-intake group were drinking more than free-intake group before start (p=0.034)	not serious	TKV increases in high intake group over 1 year	not serious	none	7
**Interventional studies (of high water intake on surrogate measures in ADPKD)**													
Low-Osmolar Diet and Adjusted Water Intake for Vasopressin Reduction in Autosomal Dominant Polycystic Kidney Disease: A Pilot Randomized Controlled Trial.<br><br>PMID: 27663039	Amro, O.W.	Am J Kidney Dis. 68(6):882-891.	2016	Interventional randomized controlled trial with 2 week follow-up	34 Patients with ADPKD aged 18-60y with eGFR >60 ml/min/1.73m². Interventional group n=17, control group n=17.	Low osmolar diet (lowNa, low protein) and adjusted water intake to achieve urine osmolality <280mOS/kg vs no intervention	Primary outcome: change in copeptin levels:<br>- significant decline of plasma copeptin level (p=0.02) and urine osmolality (p=0.01) in intervention group<br>- nonsignificant change in control group (n=17) (p=0.2 and 0.3)<br>- total urine solute decreased only in intervention group and not in control group (p=0.03)<br>- low osmolar diet leads to reduction of water required for vasopressin reduction from 3.2 to 2.6L/d.<br>>> Dietary modification of AVP and copeptin is possible.	small cohort, short follow-up (2 weeks)	serious - endpoint is copeptin levels and not disease progression	not serious	not serious	none	4
Water prescription in autosomal dominant polycystic kidney disease: a pilot study.<br><br>PMID: 20876670	Wang, C.J.	Clin J Am Soc Nephrol, 6(1):192-7	2011	Interventional study.	8 patients with ADPKD (7 female)), age 38 ± 10 years, eGFR CKD Stage 1 - 2, normal blood pressure	Phase I - normal water intake and no diet<br>Phase II - elevated water intake and no diet	Increased water intake led to:<br>- decreased urine osmolality<br>- increased urine volume<br>- no changes were observed in serum sodium, weight and blood pressure<br>>> The amount of additional water needed to achieve a urine osmolality target can be approximated from the urine osmolar excretion in ADPKD patients	very small cohort and short follow - up (2 weeks)	serious	not serious	serious	None	4

Title	First Author	Journal	Year	Study design	Nº & type of patients	Intervention/ Group difference	Outcome results	Risk of bias	Indirectness	Inconsistency	Imprecision	Other con-siderations	Importance (1-9)
A pilot clinical study to evaluate changes in urine osmolality and urine cAMP in response to acute and chronic water loading in autosomal dominant polycystic kidney disease.<br><br>PMID: 20167686	Barash, I.	Clin J Am Soc Nephrol. 5(4):693-7	2010	Interventional study on 13 Patients with ADPKD and 10 healthy controls. Comparison after fasting, acute and chronic water loading high water intake.	13 patients with ADPKD (age 38 ± 4y, 69% female, eGFR ≥ 60 ml/min/1,73m², TKV 991± 202ml) and 10 healthy controls (35 ± 4y),	Baseline: after fasting 12h, acute water intake: 2 L after fasting, chronic water intake: 3 L/d for 1 week.	Baseline after fasting 12h<br>- urine concentration was higher in healthy controls than in ADPKD (p=0.003)<br>After acute water loading:<br>- maximal dilution of urine and reduction of cAMP in both groups, no significant difference noted between the groups<br>After chronic high water intake for 1 week (3L/d)<br>- significant increase in urine volume by 64% (p<0,001) in ADPKD<br>- significant fall of urine osmolarity by 48% (P=0,04) in ADPKD<br>- no change in 24h urine cAMP during chronic water intake	small cohort, short follow-up (1 week)	serious - endpoint is urine volume and osmolality, no data on progress in CKD or TKV	not serious		None	4
**Interventional studies (of water deprivation on surrogate measures in ADPKD)**													
Urine Concentrating Capacity, Vasopressin and Copeptin in ADPKD and IgA Nephropathy Patients with Renal Impairment.<br><br>PMID: 28081165	Zittema, D.	PLoS One. 12(1):e0169263.	2017	interventional study on 15 patients with ADPKD compared to 15 patients with IgA nephropathy	15 Patients with ADPKD (49± 7 y) and 15 patients with IgA nephropathy 18-65 (49±9y); all with eGFR > 60 ml/min/1,73 m², no medication or concomitant disease	water deprivation for 16 hours in both groups and comparison of urine concentration	At baseline:<br>- no differences in total solute, urea and creatinine excretion, plasma osmolality, copeptin and circulating vasopressin (AVP)<br>After water deprivation:<br>- plasma osmolality increased significantly in ADPKD but not in IgA-nephropathy patients<br>- copeptin and AVP increased significantly in ADPKD and IgA in a similar way<br>- urine osmolality increased in both groups, but<br>- Maximal achieved urine concentration was lower in ADPKD compared to IgAN controls (p = 0.046)	serious – small cohort, very short term observation	serious – examines water deprivation rather than high water intake.	not serious	not serious	None	3
Vasopressin, copeptin, and renal concentrating capacity in patients with autosomal dominant polycystic kidney disease without renal impairment<br><br>PMID: 22516290	Zittema, D.	Clin J Am Soc Nephrol	2012	interventional study on 15 patients with ADPKD compared with 15 healthy controls	15 patients with ADPKD (36±15 y) and 15 healthy controls (35±12 y) – all eGFR > 60 ml/min/1,73 m²	water deprivation for 14 hours in both groups and comparison of urine and plasma osmolality, vasopressin and copeptin	at baseline:<br>Vasopressin and copeptin levels higher in ADPKD but not significantly<br>after 14 hours of water deprivation:<br>- higher plasma osmolality in ADPKD (p=0,07)<br>- higher vasopressin and copeptin levels (p<0,05)<br>- urine osmolality similar in ADPKD and controls p=0,61)<br>- maximal urine concentrating capacity lower in ADPKD (p<0,001)<br>- at max urine concentrating capacity, plasma osmolality, vasopressin and copeptin levels significantly higher in ADPKD<br>- increase in urine osmolality after desmopressin administration is less in ADPKD	serious – small cohort, very short term observation	serious – examines water deprivation rather than high water intake.	not serious	not serious	none	2

Title	First Author	Journal	Year	Study design	Nº & type of patients	Intervention/ Group difference	Outcome results	Risk of bias	Indirectness	Inconsistency	Imprecision	Other con-siderations	Importance (1-9)
**Association studies (of high water intake with renal outcome)**													
High urine volume and low urine osmolality are risk factors for faster progression of renal disease.<br><br>PMID: 12722030	Hebert, L.A.	Am J Kidney Dis. 41(5):962-71.	2003	Retrospective analysis in monocentric cohort of 273 patients with CKD with and without ADPKD - analysis from the MDRD study	15 Patients with ADPKD (49± 7 y) and 15 patients with IgA nephropathy 18-65y (49±9y); all with eGFR < 60 ml/min/1,73 m², no medication or concomitant disease	retrospective analysis of data from the MDRD study and correlation of eGFR decline associated with urine volume and urine osmolalitay (comparison ADPKD vs non ADPKD)	- baseline characteristics were similar in ADPKD and non-ADPKD patients assigned to same dietary group<br>- the higher mean 24h urine volume during follow-up the greater decline in GFR<br>- the lower the urine osmolality, the more rapid the GFR decline<br>- 50% reduction of magnitude of association of urine volume and urine osmolarity with eGFR decline in non-ADPKD when corrected with covariants<br>- association in ADPKD maintained after correction with covariants<br>- no significant difference between ADPKD and non-ADPKD group<br>- not clear whether high urine output is cause or result of CKD progress	serious - retrospective analysis of extrapolated data of other study	serious - retrospective analysis of extrapolated data of other study	serious - data not consistent with other studies		Different mechanisms in ADPKD with preserved or reduced renal function?	2

Abbreviations: htTKV = height corrected total kidney volume, TKV = total kidney volume, eGFR = estimated glomerular filtration rate, AVP = circulating vasopressin

Table S6b Salt intake and progression of ADPKD

Title	First Author	Journal	Year	Study design	Nº & type of patients	Intervention/ Group difference	Outcome results	Risk of bias	Indirectness	Inconsistency	Imprecision	Other con-siderations	Importance (1-9)
**Interventional studies (low salt diet on surrogate markers in ADPKD)**													
Diet and polycystic kidney disease: A pilot intervention study.<br><br>PMID: 26811129	Taylor, J.M.	Clin Nutr. 36(2):458-466	2017	Interventional study in 12 patients with ADPKD - follow up 5 weeks	11 patients with ADPKD, 47±15 years, normotensive, eGFR > 30 ml/min/1,73m², no medication or dietary restrictions beforehand	usual diet for 1 week, then 4 weeks of low-sodium, low protein, high fruit diet, with elevated water intake (urine osmolality goal < 285 mosm/kg)	- sodium excretion decreased (p<0.05)<br>- potassium excretion increased (p=0.001)<br>- urea excretion (p<0.001) and urinary calcium decreased<br>- urine volume increased 35% (p<0.001)<br>- mean osmolality declined to 285 mosm/kg H2O (p<0.001)<br>- good compliance in most patients	Small study.	serious - no outcome data or more sophisticated biomarkers	not serious	not serious	Mainly examines feasibility of an "ADPKD diet"	4
Low-Osmolar Diet and Adjusted Water Intake for Vasopressin Reduction in Autosomal Dominant Polycystic Kidney Disease: A Pilot Randomized Controlled Trial.<br><br>PMID: 27663039	Amro, O.W.	Am J Kidney Dis. 68(6):882-891.	2016	Interventional randomized controlled trial with 2 week follow-up	34 Patients with ADPKD aged 18-60y with eGFR >60 ml/min/1.73m².<br>Interventional group n=17 control group n=17.	Low osmolar diet (low Na, low protein) and adjusted water intake to achieve urine osmolality <280mOS/kg vs no intervention	Primary outcome: change in copeptin levels:<br>- significant decline of plasma copeptin level (p=0.02) and urine osmolality (p=0.01) in intervention group<br>- nonsignificant change in control group (n=17) (p=0.2 and 0.3)<br>- total urine solute decreased only in intervention group and not in control group (p=0.03)<br>- low osmolar diet leads to reduction of water required for vasopressin reduction from 3.2 to 2.6L/d.	small cohort and short follow - up (2 weeks)	serious - endpoint is copeptin levels and not disease progression	not serious	not serious	none	5
**Association studies (of low salt diet/ urinary sodium excretion with renal outcome)**													
Dietary salt restriction is beneficial to the management of autosomal dominant polycystic kidney disease.<br><br>PMID: 27993381	Torres, V.E.	Kidney Int. 91(2):493-500	2017	Post-hoc data analysis of HALT A and B-study	Study A- eGFR > 60ml/min/1.73m², n=558;<br><br>Study B – eGFR 25-60 ml/min/1.73m², n= 486	All patients on an <100 mEq sodium diet.<br>Endpoints:<br>Study A: change in TKV or eGFR<br>Study B: composite endpoint of 50% reduction in eGFR, ESRD or death	- During the trial urinary sodium excretion significantly declined by 0.25 and 0.41 mEq/24 hour per month in studies A and B.<br><u>In Study A (eGFR > 60ml/min/1,73m²)</u><br>- Averaged and time varying urinary sodium excretions were significantly associated with kidney growth (0.43%/year and 0.09%/year, respectively, for each 18 mEq urinary sodium excretion).<br>- Averaged urinary sodium excretion was not significantly associated with faster eGFR decline (-0.07 ml/min/1.73m2/year for each 18 mEq urinary sodium excretion).<br><u>In Study B (eGFR 25-60 ml/min/1.73m²)</u><br>- averaged but not time-varying urinary sodium excretion significantly associated with increased risk for the composite endpoint (hazard ratio 1.08 for each 18 mEq urinary sodium excretion) and a significantly faster eGFR decline (-0.09 ml/min/1.73m2/year for each mEq 18 mEq urinary sodium excretion)	post-hoc analysis; other dietary variations not evaluated	not serious	not serious	serious	none	7

<u>Abbreviations:</u> htTKV = height corrected total kidney volume, TKV = total kidney volume, eGFR = estimated glomerular filtration rate, AVP = circulating vasopressin

Table S6c Caffeine intake and progression of ADPKD

Title	First Author	Journal	Year	Study design	Nº & type of patients	Intervention/ Group difference	Outcome results	Risk of bias	Indirectness	Inconsistency	Imprecision	Other con-siderations	Importance (1-9)
**Association studies (of low caffeine intake with renal outcome)**													
Caffeine intake by patients with autosomal dominant polycystic kidney disease.<br><br>PMID: 22801417	Vendramini LC	Braz J Med Biol Res. 45(9):834-40	2012	cross sectional observational study of ADPKD patients and healthy controls	102 ADPKD patients (68 fem, 34 male, 39 ±12y) and 102 healthy volunteers (74 fem, 38 ±14y)	comparison of coffee consumption between ADPKD and healthy control analysis of correlation of amount of coffee consumption and TKV and eGFR	- no significant correlation between the level of caffeine intake and renal volume (r = 0.015; P = 0.879) or eGFR (r = 0.065; P = 0.520) after adjustment of covariables<br><br>- 63% of patients were previously aware of dietary advise to reduce coffee consumption<br>- coffee intake in patients with ADPKD is significantly lower than in healthy controls (caffeine from other sources was similar)	cross sectional data analysis	not serious	not serious	not serious	Detailed analysis of coffee consumption from 3d protocols.	7
Long-term effect of coffee consumption on autosomal dominant polycystic kidneys disease progression: results from the Suisse ADPKD, a Prospective Longitudinal Cohort Study<br>PMID: 28386880	Girardat-Rotar L	J Nephrol. [Epub ahead of print] doi: 10.1007/s40620-017-0396-8	2017	Observational prospective study over 6 years	151 patients with ADPKD 32.8 ± 8.9 years, 60 females	Multivariate analysis of coffee any consumption (n=101) vs no coffee (n=50) on htTKV and eGFR over 6 years	- htTKV has lower baseline values in coffee drinkers and a steeper slope over time, but data is not significant (p=0.10)<br><br>- eGFR is constantly higher in coffee drinkers than non-coffee drinkers, but no statistically significant difference in eGFR between coffee and non-coffee drinkers (2.03 ml/min/1.73 m^2, 95% CI -0.31 to 4.31, p = 0.089).	Non-coffee drinkers were younger (28 ± 8 vs. 35 ± 8 years), had smaller kidneys at baseline (753 vs. 1118 cm^3), and better renal function (eGFR 95.8 ± 19.5 vs. 88.2 ± 19.1 ml/min/1.73 m^2).	not serious	not serious	Not serious, despite many extrapolate values on coffee consumption.	No quantitative analysis of coffee consumption (only yes/no). Unclear whether patients received dietary advise to restrict coffee.	6

Abbreviations: htTKV = height corrected total kidney volume, TKV = total kidney volume, eGFR = estimated glomerular filtration rate, AVP = circulating vasopressin

Tables S7 Literature review on vasopressin inhibitors in ADPKD

Table S7a Studies assessing the EFFICACY of vasopressin receptor 2 antagonists in reducing symptoms or progression of ADPKD

Title	First Author	Journal	Year	Study design	Nº & type of patients	Outcome measure(s)	Results	Risk of bias	Indirectness	Inconsistency	Imprecision	Other considerations	Importance (1-9)
**Randomized controlled trials**													
Tolvaptan in patients with autosomal dominant polycystic kidney disease (TEMPO 3:4)<br><br>PMID: 23121377	Torres, VE	N Engl J Med. 367(25):2407-18.	2012	Randomized, Multicenter, Double-blind, placebo – controlled. 2/3 tolvaptan; 1/3 placebo	1445 participants; ages 18-50; eGFR ≥60 ml/min; TKV ≥ 750 ml	Primary outcome:<br>-TKV slope<br>Secondary outcomes:<br>- Time to 25% reduction 1/Serum creatinine<br>-Time to significant kidney pain<br>-Time to worsening of HTN (categorical)<br>-Time to increased albuminuria (categorical)	-TKV slope was significantly reduced in Tolvaptan vs placebo. 2.8% per year vs 5.5% per year.<br>- 2 vs. 5 events per 100 person-years of follow-up, P<0.001, favors Tolvaptan<br>-5 vs. 7 events per 100 person-years of follow-up, P = 0.007), favors Tolvaptan<br>- ns<br><br>- ns	Not serious, despite relatively high dropout rate (23%) in treatment arm.	Not serious	Not serious	Not serious (largest cohort to date)	Prespecified subgroup analysis showed significant effect in all stratas of age, gender, TKV at baseline, eGFR at baseline and hypertension status.	9<br><br>9<br><br>9
Tolvaptan in later-stage autosomal dominant polycystic kidney disease (REPRISE)<br>PMID: 29105594	Torres, VE	NEJM Online Nov 4, 2017 [Epub ahead of print]	2017	Randomized withdrawal, multicenter, placebo-controlled, double-blind trial.<br>1/2 tolvaptan;<br>1/2 placebo	1370 participants; ages 18-55 with eGFR 25-65 ml/min.<br>Or ages 56-65 with eGFR between 25-44 and historical evidence of progression >2ml/min/1.73 m^2 per year	Primary outcome:<br>- Pre drug baseline of the average of 3 eGFR compared to off drug follow-up of the average of 3 eGFR.<br><br>Secondary endpoint:<br>eGFR slope of multiple eGFR values	- eGFR decreased by 2.34 ml/min/1.73 m^2/year in the tolvaptan group, vs 3.61 ml/min/1.73 m^2/year (difference 1.27 ml /min/1.73 m^2; 95% CI, 0.86 to 1.68; P<0.001)<br><br>- eGFR slope was slower in tolvaptan group: −3.16 vs. −4.17 ml/min/1.73 m^2/year; p < 0.0001<br><br>Size of treatment effect similar to patients with better eGFR (TEMPO3:4)	Not serious	Not serious	Not serious	Not serious	Fewer dropouts from aquaretic effect of tolvaptan due to randomized withdrawal design.	9
Effect of tolvaptan on renal handling of water and sodium, GFR and central hemodynamics in autosomal dominant polycystic kidney disease during inhibition of the nitric oxide system: a randomized, placebo-controlled, double blind, crossover study.<br><br>PMID: 28810844	Al Therwani, S.	BMC Nephrol. 18(1):268	2017	Randomized, placebo-controlled crossover study.	18 patients with ADPKD and GFR > 30 ml/min randomized to placebo or tolvaptan and then administered L-NMMA to inhibit NO production.	Urine Osmolality, free water clearance, sodium excretion, GFR, aquaporin2, plasma vasopressin, central diastolic blood pressure.	Tolvaptan led to a less pronounced NO-inhibition-induced decrease of urine osmolality, free water clearance (43% vs 61%) and sodium excretion (41% vs 46%) than placebo.<br>GFR and u-AQP2 decreased to the same extent; p-AVP increased three fold.<br>After NO-inhibition, GFR increased after placebo and remained unchanged after tolvaptan (5% vs −6%). Central diastolic BP increased to a higher level after placebo than tolvaptan.<br>Body weight fell during tolvaptan treatment.	Serious, small, short term study.	Serious	Not serious	Small study.	Study designed more to determine role of NO in mediating tolvaptan effects.	2

Title	First Author	Journal	Year	Study design	Nº & type of patients	Outcome measure(s)	Results	Risk of bias	Indirectness	Inconsistency	Imprecision	Other con-siderations	Importance (1-9)
**Secondary Analyses of TEMPO 3:4**													
Tolvaptan and Kidney Pain in Patients With Autosomal Dominant Polycystic Kidney Disease: Secondary Analysis From a Randomized Controlled Trial.<br><br>PMID: 27856088	Casteleijn, NF	AJKD 69(2):210-219	2017	Post hoc analysis of TEMPO 3:4 study	Same as TEMPO 3:4	Kidney pain events defined by objective medical interventions in tolvaptan vs placebo-treated participants.	Tolvaptan use resulted in a significantly lower incidence of kidney pain events when compared to placebo: 10.1% versus 16.8% (P<0.001), with a risk reduction of 36% (HR, 0.64; 95% CI, 0.48-0.86). History of urinary tract infections, kidney stones, or hematuria (all P<0.001) and female sex (P<0.001) were significantly associated with history of kidney pain.	Not serious, post-hoc analysis	Not serious	Not serious	Not serious	Post hoc analysis of previously adjudicated pain events	9
Effect of Tolvaptan in Autosomal Dominant Polycystic Kidney Disease by CKD Stage: Results from the TEMPO 3:4 Trial.<br><br>PMID: 26912543	Torres, VE	Clin J Am Soc Nephrol. 11(5):803-11	2016	Post hoc analysis of TEMPO 3:4 study	Same as TEMPO 3:4	Effect of CKD class (3 subgroups) on TKV growth and eGFR decline.	Tolvaptan-induced reduction of TKV growth/year: 1.99% (CKD1), 3.12% (CKD2), and 2.61%per year (CKD3) (all P<0.001; subgroup–treatment interaction, P=0.17)<br><br>Tolvaptan-induced reduction of eGFR decline: 0.40 in CKD1 (P=0.23), 1.13 in CKD2 (P<0.001) and 1.66ml/min per $1.73m^2$ per year inCKD3 (P<0.001) with a trend for a positive subgroup-treatment interaction (P=0.07).	Not serious, post-hoc analysis				Informative for pediatrics as most children will be CKD stage 1.	8
Urine Osmolality, Response to Tolvaptan, and Outcome in Autosomal Dominant Polycystic Kidney Disease: Results from the TEMPO 3:4 Trial<br><br>PMID 27920153	Devuyst, O	JASN 28:1592-1602	2017	Post hoc analysis of TEMPO 3:4 study	Same as TEMPO 3:4	Urine osmolality as a predictor of TKV and eGFR outcomes	Among subjects receiving tolvaptan, those with a greater suppression of Uosm had slower renal function decline<br><br>Tolvaptan reduced Uosm by 200-300 mOsm/kg over 36 months	Not serious, post-hoc analysis	Not serious	Not serious	Not serious	Post hoc analysis limits generalizability but provides some guidance as to how to prescribe tolvaptan: based on Uosm response	9
Albuminuria and tolvaptan in autosomal-dominant polycystic kidney disease: results of the TEMPO 3:4 Trial<br><br>PMID: 26681730	Gansevoort, RT	NDT 31:1887-1894	2016	Post hoc analysis of TEMPO 3:4 study	Same as TEMPO 3:4	Albuminuria as a continuous variable; TKV and eGFR slopes as a function of baseline albuminuria	Baseline median (interquartile range) ACR was 3.2 (1.7-7.1) mg/mmol. 47.9% of ADPKD patients had normal, 48.7% moderately increased and 3.4% severely increased ACR.<br>ACR rose in placebo- and decreased in tolvaptan-treated patients (+0.23 versus -0.40 mg/mmol). The difference in ACR increased over time, reaching a maximum of 24% at month 36 (P < 0.001).<br>Beneficial effect of tolvaptan on TKV growth and eGFR loss was stronger in patients with higher baseline ACR.	Not serious, post-hoc analysis	Not serious	Not serious	Not serious	Albuminuria is not a prominent finding in ADPKD and the findings will apply to limited numbers of patients	6

Title	First Author	Journal	Year	Study design	Nº & type of patients	Outcome measure(s)	Results	Risk of bias	Indirectness	Inconsistency	Imprecision	Other con-siderations	Importance (1-9)
Prognostic Enrichment Design in Clinical Trials for Autosomal Dominant Polycystic Kidney Disease: The TEMPO 3:4 Clinical Trial<br><br>PMID: 27484667	Irazabal, M	Kidney Int Rep 1, 213–220	2016	Post hoc analysis of TEMPO 3:4 study	Same as TEMPO 3:4. 9 patients excluded due to lack of baseline images, heightor incorrect diagnosis.	Effect of Mayo imaging-based classification at baseline on TKV and eGFR slope (and how this could help to select patients for future trials).<br><br>Class 1 (typical) vs class 2 (atypical). Class 1 divided A-E based on htTKV for age (where A has lowest htTKV for age an slowest progression).	Treatment effect on %TKV growth/year<br>Class 1B: -2.00 %/year, p=0.02<br>Class 1C: -3.27%/year, p<0.001<br>Class 1D: -2.52%/year, p<0.001<br>Class 1E: -2.66%/year, p<0.001<br>Class 2A/2B: -0.21%/year, p=0.88<br>Treatment effect on eGFR decline/year<br>Class 1B: -0.31 ml/min*1.73m²/year, p=0.64<br>Class 1C: 1.27 ml/min*1.73m²/year, p<0.001<br>Class 1D: 0.89 ml/min*1.73m²/year, p=0.007<br>Class 1E: 1.47 ml/min*1.73m²/year, p=0.002<br>Class 2A/2B: 0.32 ml/min*1.73m²/year, p=0.75<br><br>Restricting enrollment to classes 1C to E would have reduced TKV and eGFR slopes from 5.78% to 2.91% per year and from -3.93 to -2.82 ml/min/1.73 m² per year, and the risk of the composite endpoint (hazard ratio = 0.84, P = 0.003), with 10.5% fewer patients.	Not serious	Not serious	Not serious	Not serious	Prognostic enrichment criterion for a study that defined those more likely to respond; TEMPO 3:4 population already enriched by large TKV	6
Can we further enrich autosomal dominant polycystic kidney disease clinical trials for rapidly progressive patients? Application of the PROPKD score in the TEMPO trial.<br><br>PMID: 28992127	Cornec-Legaul, E.	Nephrol Dial Transplant [Epub ahea of print]	2017	Post hoc analysis of TEMPO 3:4 study	749 subjects for whom genotype was known<br><br>Low risk (score 0-3 points) n = 132, intermediate risk (4-6 points) n = 344 and high risk (7-9 points) n= 273	Effect of PROPKD risk score classification at baseline on TKV and eGFR slope (and how this could help to select patients for future trials).<br><br>PROPKD score: sum of: male (1 point), hypertension < 35 years (2 points), cyst infection, macrohematuria or flank pain < 35 years (1 point), PKD2 mutation (0 points),non-truncating PKD1 (2 points), truncating PKD1 (4 points).	Treatment effect on %TKV growth/year<br>Low risk: reduced by 45.8%, p=0.0022<br>Intermediate: reduced by 51.8%, p<0.0001<br>High risk: reduced by 38%, p<0.001<br><br>Treatment effect on eGFR decline/year<br>Low risk: reduced by 6.9%, p=0.72<br>Intermediate: reduced by 30.3%, p=0.008<br>High risk: reduced by 30.6%, p=0.002<br><br>Excluding the low risk subjects from the analysis improved the apparent treatment effect of tolvaptan on eGFR decline.	Not serious	Not serious	Not serious	Not serious		6
**Non-randomized studies**													
Multicenter, open-label, extension trial to evaluate the long-term efficacy and safety of early versus delayed treatment with tolvaptan in autosomal dominant polycystic kidney disease: the TEMPO 4:4 Trial.<br><br>PMID: 28379536	Torres, VE	Nephrol Dial Transplant . 2017 Mar 31. [Epub ahead of print]	2017	2 year open-label extension study for efficacy and safety.<br>No control group.	871 (60.3% of TEMPO 3:4 participants). eGFR > 30 ml/min.<br>(n=557 with prior tolvaptan (=early treatment) and n=314 with prior placebo (=delayed treatment).<br>13-829 days off treatment.	primary endpoint:<br>- change in TKV from TEMPO 3:4 baseline to TEMPO 4:4 Month 24 in early- versus delayed-treated subjects<br><br>secondary endpoints:<br>- changes in eGFR from TEMPO 3:4 baseline to TEMPO 4:4 Month 24<br><br>- TKV and eGFR slopes during TEMPO 4:4 in early- and delayed-treated subjects.	-Percent changes in TKV were 29.9% in early vs 31.6% in delayed treatment (P = 0.38).<br>Adjusting for baseline covariates improved TKV treatment difference at Month 24 from -1.70% to -4.15% between the groups (P = 0.04).<br><br>- persistent effect on eGFR (3.15 mL/min/1.73 m², P < 0.001)<br><br>- non-inferiority in eGFR slopes btwn early and delayed treated.<br>- higher TKV growth slopes in early- vs delayed-treatment groups (6.16% vs 4.96%/year, P = 0.05).						8

Title	First Author	Journal	Year	Study design	Nº & type of patients	Outcome measure(s)	Results	Risk of bias	Indirectness	Inconsistency	Imprecision	Other con-siderations	Importance (1-9)
**Short term effects**													
Short-term Effects of Tolvaptan in Individuals With Autosomal Dominant Polycystic Kidney Disease at Various Levels of Kidney Function<br><br>PMID: 25600953	Boertien, WE	AJKD 65(6):833-41	2015	Before and after 3 weeks of tolvaptan in ADPKD subjects; no control group	27 participants with mGFR of 18-148 mL/min. Ages 46 ± 10	Markers of aquapheresis (free-water clearance, urine and plasma osmolality, 24-hour urine volume, and plasma copeptin) and kidney injury (TKV and kidney injury biomarkers)	Sgnificant increase in urine volume and free-water clearance and a decrease in urine osmolality.<br>Short term decrease in mGFR (mL/min) from 69.1±38.6 to 64.4±35.7 (-5.4%±8.0%, p=0.002) and TKV (mL) from 2,147 [1,100 to 2,767] to 2,052 [1,040 to 2,690] (p=0.001).<br>Changes in urine volume and osmolality less in patients with lower baseline mGFRs (both P<0.01).	Not serious	Not serious	Not serious	Not serious	Small study; short duration but confirms short-term effect of tolvaptan	6
Short-term effects of tolvaptan on renal function and volume in patients with autosomal dominant polycystic kidney disease<br><br>PMID: 21544064	Irazabal, M	Kidney Int. 80(3):295-301	2011	Before and after 8 days of tolvaptan in ADPKD subjects; no control group	20 participants Mean age 48; Mean GFR 69; Mean TKV 2316 ml	Pre- vs post measures of GFR, TKV, free water clearance	Tolvaptan induced aquaresis (free water clearance increased by 92%).<br><br>Significant acute reduction in GFR by 8.6% and TKV decreased by 3.1%.	Not serious	Not serious	Not serious	Not serious	Small study; short duration but confirms short-term effect of tolvaptan	6
**Case report in children**													
Tolvaptan treatment for severe neonatal autosomal-dominant polycystic kidney disease.<br><br>PMID: 28194574	Gilbert, R.D.	Pediatr Nephrol. 32(5):893-896	2017	Case report. Female with biallelic *PKD1*. Prenatally enlarged kidneys and low-normal amniotic fluid.	Postnatal resuscitation for pulmonary hypoplasia with massively enlarged kidneys. Neonatal hyponatremia and oedema. Later hypertension	Narrative.<br><br>Initial dose 0.15 mg/kg/day, started at day 31.<br>Increased to 0.5 mg/kg at 3 months, 0.7 mg/kg at 6 months, 1 mg/kg at 9 months.<br><br>Initial 4 hourly monitoring.	Tolvaptan produced the expected aquaresis and blood pressure reduction.<br>Electrolyte supplements needed adjusting.<br><br>At 18 months: stable GFR around 60 ml/min*1.73m², kidney size stable since birth.<br>Respiratory distress much improved.<br>Normal development.<br>No problems with hypernatraemia, hepatotoxicity or polyuria.	Serious, single case report without control.	Not serious	Not serious	Serious,	ARPKD-like presentation is much rarer than mild courses	4

Table S7b Studies on the SAFETY of vasopressin receptor 2 antagonists in ADULTS with ADPKD

Title	First Author	Journal	Year	Study design	Nº & type of patients	Outcome measure(s)	Results	Risk of bias	Indirectness	Inconsistency	Imprecision	Other considerations	Importance (1-9)
**Randomized controlled trials**													
Tolvaptan in patients with autosomal dominant polycystic kidney disease (TEMPO 3:4)<br><br>PMID: 23121377	Torres, VE	N Engl J Med. 367(25):2407-18.	2012	Randomized, Multicenter, Double-blind, placebo – controlled. 2/3 tolvaptan; 1/3 placebo	1445 participants; ages 18-50; eGFR ≥60 ml/min; TKV ≥ 750 ml	Adverse events categorized according to Medical dictionary for regulatory activities.<br><br>4-monthly liver function tests.	adverse events overall:<br>tolvaptan group 97.9%, placebo 97.1% (p=ns)<br>aquaresis related side effects:<br>thirst, polyuria, nocturia, frequency, polydipsia<br>tolvaptan group: 55%, 38%, 29%, 23%, 10% vs placebo: 21%, 17%, 13%, 5% and 4% (all p<0.001)<br>ADPKD related adverse events:<br>Renal pain, hematuria, UTI<br>Tolvaptan group:27%, 8%, 8% vs Placebo: 35% (p<0.05), 14% (p<0.001), 13% (p<0.05)<br>drug discontinuation due to side effects:<br>tolvaptan group 15.4%, placebo 5%%<br>Serious adverse events (all p=ns):<br>More common with tolvaptan:<br>AST and ALT elevation (0.9 vs 0.4% for both), chest pain (0.8 vs 0.4%) and headache (0.5 vs 0%).<br>More common with placebo:<br>Pyelonephritis, renal cyst infection and hemorrhage, renal pain, appendicitis, nephrolithiasis, UTI and hypertension.<br>Hypernatremia > 150 mmol/l:<br>Tolvaptan 4%, vs placebo 1.4%	Not serious	Not serious	Not serious	Not serious (largest cohort to date)	No deaths during the trial.<br>In the tolvaptan group 8.3% of patients discontinued due to aquaresis related symptoms and 1.2% due to liver-function abnormalities.<br>2 patients with concurrent elevation of AST or ALT (≥ 3x ULN) and bilirubin (≥ 2x ULN) which resolved in all cases.	9
Tolvaptan in later-stage autosomal dominant polycystic kidney disease (REPRISE)<br>PMID: 29105594	Torres, VE	NEJM Online Nov 4, 2017 [Epub ahead of print]	2017	Randomized withdrawal, multicenter, placebo-controlled, double-blind trial.<br>1/2 tolvaptan;<br>1/2 placebo	1491 received tolvaptan dose-adjustment run-in (5 weeks), and 681 received tolvaptan for 12 months.	Adverse event monitoring.<br><br>Monthly monitoring of liver function tests for 18 months; quarterly thereafter	ALT > 3x upper limit of normal of 5.6% vs 1.2%, all reversible after stop of tolvaptan; no cases meeting Hy's law of serious drug-induced liver injury with monthly monitoring<br><br>Fewer dropouts from aquaretic effect of tolvaptan due to randomized withdrawal design.	Not serious	Not serious	Not serious	Not serious		9
**Secondary Analyses of TEMPO 3:4**													
Polyuria due to vasopressin V2 receptor antagonism is not associated with increased ureter diameter in ADPKD patients.<br><br>PMID: 27339446	Casteleijn, NF	Clin Exp Nephrol. 21(3):375-382	2017	Retrospective analysis of TEMPO 3:4 study and 284 trial at one study site.	70 ADPKD (51 tolvaptan and 19 placebo). 65.7 % male, age 41 ± 9 years, mGFR 74 ± 27 mL/min/1.73 m^2 and TKV 1.92 (1.27–2.67) L	At baseline and after 3 years of treatment ureter diameter at the levels of renal pelvis and fifth lumbar vertebral body (L5) was determined by MRI (taken for TKV).	Baseline:<br>no differences in 24-h urine volume or ureter diameter (renal pelvis: 4.0 vs. 4.2 mm, p = 0.4 and L5: 3.0 vs. 3.1 mm, p = 0.3).<br><br>After 3 years of treatment<br>24-h urine volume was higher in tolvaptan-treated patients compared to placebo (4.7 vs. 2.3 L, p<0.001),<br>no differences in ureter diameter (renal pelvis: 4.2 vs. 4.4 mm, p = 0.4 and L5: 3.1 vs. 3.3 mm, p = 0.4).	Not serious, post-hoc analysis	Not serious	Not serious	Not serious		7

Title	First Author	Journal	Year	Study design	Nº & type of patients	Outcome measure(s)	Results	Risk of bias	Indirectness	Inconsistency	Imprecision	Other con-siderations	Importance (1-9)
Clinical Pattern of Tolvaptan-Associated Liver Injury in Subjects with Autosomal Dominant Polycystic Kidney Disease: Analysis of Clinical Trials Database<br><br>PMID: 26188764	Watkins, PB	Drug Safety 38:1103-13	2015	Post hoc analysis of TEMPO 3:4 study also reviewing other tolvaptan trials in heart failure and hyponatremia	6155 subjects were enrolled (tolvaptan, 3403; placebo, 2752) across all non-ADPKD trials, and 4664 subjects (tolvaptan,2414; placebo, 2250) were enrolled in long-term placebo-controlled trials	Blinded Evaluation of Drug-Induced Serious Hepatotoxicity (eDISH) approach by hepatologists with expertise in drug induced liver injury (DILI)	In TEMPO 3:4, the 16 cases with ALT elevations >3x ULN probable or highly likely attributable to tolvaptan were detected 3 to 18 months after initiation.<br>For the two Hy's Law cases, ALT elevations >3x ULN first occurred between 5 and 9 months after start of tolvaptan.<br>All 35 cases adjudicated in the tolvaptan group returned to <3x ULN. Most who discontinued tolvaptan (14/35) returned to <3x ULN within 40 days, while those who continued therapy (21/35) returned to <3x ULN within 120 days.<br>> Appearance of possible hepatic injury is during the first 18 months of exposure to tolvaptan Monthly monitoring and treatment interruption appears to reverse the elevated ALT.<br>> Serious DILI did not occur with tolvaptan for non-ADPKD indications.	Not serious	Not serious	Not serious	Not serious	Definition of time frame for hepatotoxicity of tolvaptan; important guidance for treatment	8
Effect of Tolvaptan in Autosomal Dominant Polycystic Kidney Disease by CKD Stage: Results from the TEMPO 3:4 Trial.<br><br>PMID: 26912543	Torres, VE	Clin J Am Soc Nephrol. 11(5):803-11	2016	Post hoc analysis of TEMPO 3:4 study	Same as TEMPO 3:4	Same as TEMPO 3:4	Aquaresis-related adverse events (more frequent in the tolvaptan group) and ADPKD-related adverse events (more frequent in the placebo group) were not associated with CKD stage.<br>Hypernatremia events in tolvaptan-treated patients with CKD3 and plasma aminotransferase elevations in tolvaptan-treated patients across CKD stages 1–3 occurred more frequently than in placebo recipients.	Not serious, post-hoc analysis	Not serious	Not serious	Not serious	clinically similar beneficial effects of tolvaptan in ADPKD across CKD stages 1–3.	7
**Non-randomized studies**													
Multicenter, open-label, extension trial to evaluate the long-term efficacy and safety of early versus delayed treatment with tolvaptan in autosomal dominant polycystic kidney disease: the TEMPO 4:4 Trial.<br><br>PMID: 28379536	Torres, VE	Nephrol Dial Transplant. 2017 Mar 31. [Epub ahead of print]	2017	2 year open-label extension study for efficacy and safety.<br>No control group.	871 ADPKD patients with eGFR > 30 ml/min.<br>(n=557 with prior tolvaptan and n=314 with prior placebo (=delayed treatment).	Adverse event monitoring.<br><br>Liver function tests at 0,3,6 months and 6-monthly thereafter.	Similar safety profile to TEMPO 3:4.<br>Aquaretic adverse events were more frequently reported in the delayed-treatment group who had not been previously exposed to tolvaptan.<br>ALT or AST ≥ 3 x ULN: 2.5% in early treatment vs 3.8% in delayed treatment groups (similar to TEMPO 3:4.<br>Drug-induced liver injury fulfilling Hy's law (ALT >3x ULN, BT >2x ULN, alkaline phosphatase <2x ULN and no alternative explanation): 1 in delayed treatment group. Recovered completely after drug discontinuation.					4 deaths all occurred after discontinuation of tolvaptan.	8
Long-term safety profile of tolvaptan in autosomal dominant polycystic kidney disease patients: TEMPO Extension Japan Trial.<br><br>PMID: 29123425	Muto, S	Drug Healthc Patient Saf 25;9:93-104	2017	3 year open-label extension study for safety.<br>No control group.	135 Japanese patients from TEMPO 3:4	Adverse event monitoring.<br><br>Liver function tests at week 1,2, and 3, month 3 and every 3 months thereafter.	Similar side effect profile to TEMPO 3:4 without unexpected adverse events.<br>Hyperuricaemia in 15%.<br>Fourteen patients (10.4%) experienced hepatic events, and 8 (5.9%) experienced >3x ULN of AST or ALT between 3 and 9 months following tolvaptan initiation, which recovered after drug interruption. No cases met Hy's law.	Not serious	Not serious	Not serious	Not serious		8
**Case reports**													
Acute pulmonary thromboembolism occurring during treatment with tolvaptan in a patient with autosomal-dominant polycystic kidney disease.<br><br>PMID: 28509130	Morimoto, K	CEN Case Rep. 6(1):61-65	2017	Case report	60 year old male	Narrative	During 8th month of treatment.<br>Sudden onset of chest pain and dyspniea after 6 days of persistent watery diarrhea. Pulmonary embolisms diagnosed on contrast-enhanced CT with corresponding laboratory abnormalities.<br>Tx: Discontinuation of tolvaptan, i.v. fluid loading, monteplase. Thrombi resolution on day 14, discharge on day 18.	Serious, case report					3

Table S7c Studies on the SAFETY of vasopressin receptor antagonists IN CHILDREN (for any indication)

Title	First Author	Journal	Year	Study design & indication	Nº & type of patients exposed	Dosing, reported effects & side effects	Risk of bias	Indirectness	Inconsistency	Imprecision	Other considerations	Importance (1-9)
**Renal indications**												
Tolvaptan treatment for severe neonatal autosomal-dominant polycystic kidney disease.<br><br>PMID: 28194574	Gilbert, R.D.	Pediatr Nephrol. 32(5):893-896	2017	Case report	1 Female with biallelic *PKD1* mutation and ARPKD-like presentation (details see above).<br><br>Unstable effects with crushed tablets. Much more stable effects with administration of a suspension	Initial dose 0.15 mg/kg/day, started at day 31.<br>Increased to 0.5 mg/kg at 3 months, 0.7 mg/kg at 6 months, 1 mg/kg at 9 months.<br>Acute blood pressure reduction with onset of aquaresis.<br>Electrolyte supplements needed adjusting.<br><br>At 18 months: stable GFR around 60 ml/min*1.73m². Normal development.<br>No problems with hypernatraemia, hepatotoxicity or polyuria.	Serious, single case.	Not serious	Not serious	Serious,	Only medium length of follow-up.<br>Wide dose range.	4
Tolvaptan therapy for massive edema in a patient with nephrotic syndrome.<br><br>PMID: 24240509	Shimizu, M.	Pediatr Nephrol. 29(5):915-7	2014	Case report. Refractory edema in nephrotic syndrome.	16 year old girl with relapse of steroid-dependent nephrotic syndrome & acute renal failure, pulmonary oedema, pleural effusion and ascites. Failure of albumin infusions, diuretics and extracorporeal ultrafiltration.	After tolvaptan 7.5 mg/day did not increase serum sodium, dose was increased to 15 mg after 3 days. Dramatic improvement of edema with reduction of body weight from 54 kg to 38 kg (dry weight).<br><br>No hypernatremia or liver function abnormality.<br>Proteinuria remained stable (very large).	Serious, case report.				Length of treatment and follow-up not mentioned.	3
Tolvaptan in a pediatric patient with diuretic-resistant heart and kidney failure.<br><br>PMID: 25711263	Hirano, D.	Pediatr Int. 57(1):183-5	2015	Case report. Congestive heart failure and advanced CKD	10-year-old boy with trisomy 18, congestive heart failure, CKD (creatinine 6.8 mg/dl). Edema, hypertension and pleural effusion.	Tolvaptan (0.1 mg/kg/day) led to a maintained urine volume of approximately 1000 mL/day and low serum excretion (<40 mmol/day), as well as increased free water clearance.<br>Sodium increased to 138 mmol/L, bodyweight decreased to 15 kg, and cardiac function improved, doses of furosemide and spironolactone could be reduced to one-third initial dose.	Serious, case report.				Length of follow-up unclear (only 12 days?)	3
**Hyponatremia in Heart failure [licensed indication in adults]**												
Efficacy and safety of tolvaptan for pediatric patients with congestive heart failure. Multicenter survey in the working group of the Japanese Society of PEdiatric Circulation and Hemodynamics (J-SPECH).<br><br>PMID: 26710331	Higashi, K.	Int J Cardiol. 15;205:37-42	2016	Multicenter, retrospective, observational study. Congestive heart failure	34 children with mean age 49.1 ± 60.8 months (range 2–202 months). 24 patients (70.6%) < 48 months.<br><br>Prevalence of class III and IV NYHA heart failure 79%.<br><br>Indication: hyponatremia (n = 23), severe congestion (n = 17), oliguria (n = 15), and pulmonary effusions (n = 9).	Mean starting dose of tolvaptan 0.25 ± 0.18 mg/kg/day (range 0.02–0.76 mg/kg/day).<br>Observation period: 1 month, but 59% were stopped beforehand.<br>Significant increases of:<br>- urinary volume at day 1, day 3, week 1, and month 1 (all p<0.05).<br>- serum sodium at day 1, day 3, week 1, and month 1 (all p<0.05).<br>- serum osmolality at day 3 and month 1 (p < 0.05)<br>Significant decreases of:<br>- body weight at day 1, day 3, week 1, and month 1 (all p<0.05).<br>- urinary osmolality at day 1 and day 3 (p < 0.05)<br>- urinary specific gravity at day 1, day 3, and week 1 (all p < 0.02)<br>8/34 (24%) non-responders (urinary volume not increased on day 1).<br>Logistic regression showed urinary osmolality as only significant predictor of tolvaptan-response.<br>Adverse drug reactions were observed in 7 patients (20.6%):<br>6 had thirst and dry month, 1 mild increase in AST and ALT.	Small, retrospective, non-controlled study.	Serious, different medical setting to ADPKD in seriously ill patients already on multiple drug therapy.			Liver function tests not reported.	4

Title	First Author	Journal	Year	Study design & indication	Nº & type of patients exposed	Dosing, reported effects & side effects	Risk of bias	Indirectness	Inconsistency	Imprecision	Other con-siderations	Importance (1-9)
Tolvaptan increases serum sodium in pediatric patients with heart failure.<br><br>PMID: 23463133	Regen, R.B.	Pediatr Cardiol. 4(6):1463-8	2013	Single center, retrospective, observational study.	28 patients, median age 2 years (1 month to 18 years).<br><br>Single dose	Median tolvaptan dose 0.3 mg/kg (range 0.1-1.3 mg/kg.<br>Observation period 48 hours.<br>Mean increases of serum sodium 2.5 mmol/L at 12 h, 5 mmol/L at 24 h, 4 mmol/L at 48 h, and 5 mmol/L at 72 h (all p < 0.001).<br>Urine output was increased at 24 h (p < 0.001) and 48 h (p = 0.03), and fluid balance changes were significantly different at 24 h (p = 0.004).	Small, retrospective, non-controlled study.	Serious, different medical setting to ADPKD.			Very short observation period. Liver function tests not analyzed.	3
Use of Tolvaptan in a Patient With Palliated Congenital Heart Disease.<br><br>PMID: 27613391	Chakravarti, S,	World J Pediatr Congenit Heart Surg. [Epub ahead of print]	2016	Case report. Chronic heart failure	1 adolescent with chronic heart failure in the setting of palliated congenital heart disease prior to definitive surgical intervention	Improvement in hyponatremia and a decrease in body weight, without any adverse effects.<br>Short-term use.<br>No side effects	Serious, case report.	Serious, different medical setting to ADPKD.				2
Effects of tolvaptan on congestive heart failure complicated with chylothorax in a neonate.<br><br>PMID: 26508187	Sato. N.	Pediatr Int. 57(5):1020-2	2015	Case report. Congestive heart failure and chylothorax.	1 neonate after palliative surgery for transposition of the great arteries.	Slow up-titration to 0.1 mg/kg.<br>Started on day 31 of life and continued until 6 months of age.<br>Increased urine output and improved refractory congestive heart failure. Bodyweight and chylothorax decreased gradually<br>Furosemide could be reduced.<br>No hypernatremia or other adverse effects.	Serious, case report	Serious, different medical setting to ADPKD.			Very low dose. Liver function tests not reported but probably done	2
Novel Use of Tolvaptan in a Pediatric Patient With Congestive Heart Failure Due to Duchenne Muscular Dystrophy and Congenital Adrenal Hyperplasia.<br><br>PMID: 26472954	Sami, S.A.	J Pediatr Pharmacol Ther. 20(5):393-6	2015	Case report. Congestive heart failure due to muscle disease and hyponatremia due to hypoadrenalism.	17-year-old Caucasian male with severe Duchenne muscular dystrophy causing congestive heart failure, and congenital adrenal hyperplasia causing repeated hyponatremias.	Tolvaptan started in non-emergency setting with 7.5 mg at ~38 kg body weight.<br>Observation period: 28 months.<br>Normalization of serum sodium within 48 hours.<br>No further hyponatremia-related admissions (repeatedly before) and no adverse reactions or significant change in liver function profile.	Serious, case report	Serious, different medical setting to ADPKD.			Liver function tests not reported but probably done.	2
Effect of the oral vasopressin receptor antagonist tolvaptan on congestive cardiac failure in a child with restrictive cardiomyopathy.<br><br>PMID: 23388069	Horibata, Y.	Cardiol Young. 24(1):155-7	2014	Case report. Congestive heart failure.	6-year-old boy with advanced congestive heart failure due to restrictive cardiomyopathy with severe pleural effusions, ascites and hyponatremia.	Initial dose 0.17 mg/kg/day, gradually up-titrated to 0.42 mg/kg/day.<br>Observation time: 47 days.<br>Gradual improvement of body weight and effusions, normalization of serum sodium.<br>No changes in serum creatinine, blood urea nitrogen, and potassium.	Serious, case report	Serious, different medical setting to ADPKD.			Liver function tests not reported	2

Title	First Author	Journal	Year	Study design & indication	Nº & type of patients exposed	Dosing, reported effects & side effects	Risk of bias	Indirectness	Inconsistency	Imprecision	Other con-siderations	Importance (1-9)
**SIADH (syndrome of inappropriate ADH secretion) [licensed indication in adults]**												
Tolvaptan utilization in children with chronic hyponatremia due to inappropriate antidiuretic hormone secretion (SIADH). Three case reports and review of the literature.<br><br>PMID: 28515029	Tuli, G	J Clin Res Pediatr Endocrinol. 2017 May 17. [Epub ahead of print]	2017	Case series of 3 pediatric cases.	Case1: 8 year old with hypothalamic ADH dysregulation in syndromic disease<br>Case 2: 4 year old with SIADH due to sellar tumor.<br>Case 3: 5 year old with SIADH due to sellar tumor. Symptomatic hyponatremia with headache, nausea, and asthenia.<br>All 3 children had hyponatremic seizures and also central hypoadrenalism	Case 1: tolvaptan started at 3.75 mg (0.06 mg/kg/day), increased over few days to 11.25 mg (0.2 mg/kg/d).<br>Observation period: 4 years: stable serum sodium levels no acute or severe symptoms due to hyponatremia have been observed.<br>Case 2: tolvaptan was started at 0.1 mg/kg/day, then increased to 0.22 mg/kg/day. 3 years later 11.25 mg/day (0.32 mg/kg/day).<br>Observation period: 3 years: normalization of serum sodium, no seizures, no hypernatremia, despite new central hypoadrenalism due to tumor growth.<br>Case 3: tolvaptan started at 0.05 mg/kg/day. After 3 months, the tolvaptan dosage has been increased to 7.5 mg (0.09 mg/kg/day).<br>Observation period: 3 months:<br>prompt normalization of serum sodium, reduction of sodium supplements.	Serious, case series	Serious, different medical setting to ADPKD.			Liver function tests not reported but probably done.	2
Tolvaptan use during hyperhydration in paediatric intracranial lymphoma with SIADH.<br><br>PMID: 27857840	Willemsen, RH	Endocrinol Diabetes Metab Case Rep. 2016. pii: 16-0066. Epub 2016 Nov 1.	2016	Case report. Malignant SIADH	11-year-old boy with severe SIADH due to intracranial B-cell lymphoma	Tolvaptan started at 0.14 mg/kg od and titrated up to 0.28 mg/kg twice a day. Total length about 14 days.<br>Observation time: 2 years<br>Tolvaptan gradually increased sodium levels and allowed liberalization of fluid intake and hyperhydration for chemotherapy.<br>Urine output increased up to 8 mL/kg/h, requiring close monitoring of fluid balance, serum sodium and fluid intake.<br>Stopped after ~14 days due to improvement of SIADH.<br>SE: desquamative rash on the dorsal side of both hands (while also receiving chemotherapy).	Serious, case report	Serious, different medical setting to ADPKD.			Short term use	
**Hyponatremia in cirrhosis of the liver [licensed indication in adults]**												
No reports found												
**Capillary leak after cardiopulmonary bypass surgery**												
Safety and effectiveness of tolvaptan for fluid management after pediatric cardiovascular surgery.<br><br>PMID: 28647800	Katayama, Y.	Gen Thorac Cardiovasc Surg. 65(11):622-626	2017	Retrospective case-control study. Postoperative fluid retention after surgery with cardiopulmonary bypass.	Children with uncomplicated congenital heart disease undergoing open heart surgery, > 4 kg body weight. N=18 standard care (oral diuretics) vs n=25 with additional single dose of tolvaptan (0.45 mg/kg).<br>Mean age 27.5 ± 37.8 months (median 12 months, range 2–192 months).	Single dose of tolvaptan 0.45 mg/kg in combination with the initial conventional oral diuretics.<br>Observation time: 24 hours.<br>Treated group had lower:<br>- dose of additional intravenous diuretics (p = 0.001).<br>- decrease in central venous pressure (p = 0.019).<br>Treated group had higher:<br>- urinary output (54.3 ± 4.5 vs 47.3 ± 19.1 mL/kg; p = 0.043),<br>No difference between groups in<br>- electrolyte concentrations in blood and urine<br>- urine gravity (increased in both groups)<br>- pre-operative data, length of OP, intubation & hospital stay.	Small study, short follow-up.	Serious, different medical setting to ADPKD.			Single dose. Historical control group	3

Tables S8 Literature review on statin therapy in ADPKD, with a special focus on children and adolescents

Table S8a Trials of statins in ADPKD

Title	First Author	Journal	Year	Study design	Nº & type of patients	Outcome measure(s)	Results	Risk of bias	Indirectness	Inconsistency	Imprecision	Other con-siderations	Importance (1-9)
**Children**													
Effect of pravastatin on total kidney volume, left ventricular mass index, and microalbuminuria in pediatric autosomal dominant polycystic kidney disease<br><br>PMID 24721893	Cadnapaphornchai, M.A.	Clin J Am Soc Nephrol 9:889-96	2014	Randomized prospective double-blind placebo controlled phase 3 clinical trial	110 pediatric and young adults with ADPKD 8-22 y with Schwartz eGFR > 80 mL/min/1.73m² treated with lisinopril targeting 50-75th percentile BP	Baseline, 18 month, 36 month assessment of:<br>Primary combined outcome of ≥20% increase in any of HtTKV (MRI), LVMI, or UAE<br>Secondary outcomes of ≥20% increase and percent change over study in individual variables (HtTKV, LVMI, or UAE)<br><br>Pravastatin dose determined by age:<br>20 mg/d in 8-12y,<br>40 mg/d in ≥13y	83% overall completion rate<br>-Statin enrolled n=56, completed n=49<br>-Placebo enrolled n=54, completed n=42<br><br>Significant decrease in total and LDL cholesterol in statin-treated group<br><br>34 participants (69%) in statin group demonstrated of ≥20% increase in any of HtTKV, LVMI, or UAE as compared with 37 (88%) in placebo group (p=0.03)<br>-this finding was related primarily to change in HtTKV with 46% of statin group and 68% of placebo group demonstrating ≥20% increase over study (p=0.03)<br>--Percentage demonstrating ≥20% increase in LVMI was not significant between statin and placebo groups (p=0.18)<br>--Percentage demonstrating ≥20% increase in UAE was not significant between statin and placebo groups (p=0.5)<br><br>With adjustment for age, sex, and baseline hypertension status (Y/N), percent change in HtTKV over study was 31 ± 3 statin vs 23 ± 3% over 3 years placebo (p=0.01)<br><br>No significant difference in eGFR over course of study<br>No correlation between LDL or total cholesterol and any of TKV, HtTKV, UAE, LVMI, creatinine clearance	Not serious	Not serious	Not serious (no change in eGFR detected similar to Fassett study but decreased eGFR would be unusual in pediatric population;)	Not serious? (overall small sample size compared to adult studies; Utilized MRI; Drop out rate)		9
Pravastatin therapy and biomarker changes in children and young adults with autosomal dominant polycystic kidney disease<br><br>PMID 26224879	Klawitter, J.	Clin J Am Soc Nephrol 10:1534-41	2015	Analysis of samples from pediatric participants in CJASN publication above	See above	Change in plasma and urinary biomarkers of endothelial dysfunction, oxidative stress, inflammation by mass spectometry over 3 year study in pravastatin vs placebo groups	ADPKD associated with increased plasma level of proinflammatory and oxidative stress markers 9-HODE, 13-HODE, 15-HETE. These markers increased in placebo group but declined with pravastatin treatment over time<br><br>Urinary 8-HETE, 9-HETE, 11-HETE positively associated with change in HtTKV in statin group, ? enhanced elimination in statin group	Not serious	Serious		Potentially serious due to limited norms and limited sample size	88/110 plasma and 85/110 urine samples available for analysis<br><br>Limited availability of normal and age-specific ranges for markers<br><br>Causality/clinical relevance not established	3

Title	First Author	Journal	Year	Study design	Nº & type of patients	Outcome measure(s)	Results	Risk of bias	Indirectness	Inconsistency	Imprecision	Other con-siderations	Importance (1-9)
**Adults**													
**Effects on disease progression (GFR or TKV)**													
Renal hemodynamic effects of the HMG-coA reductase inhibitors in autosomal dominant polycystic kidney disease<br><br>PMID 26614268	Zand, L.	Nephrol Dial Transplant 31:1290-5	2016	Open label	32 adults including:<br>11 ADPKD with “mild” renal dysfunction (SCr ≤ 1.3 mg/dL, iothalamate clearance ≥ 75 mL/min/1.73m²)<br>11 ADPKD with “moderate” renal dysfunction (SCr ≤ 1.6 mg/dL, iothalamate clearance ≥ 65)<br>11 healthy volunteers<br><br>Simvastatin 40 mg/d for 4 weeks	Baseline and 4 week assessment of GFR/estimated renal plasma flow (ERPF) by iothalamate/para-amino-hippurate (PAH) clearance and renal blood flow (RBF) by contrast MRI	At baseline:<br>-Mean age 49y (control), 47 y (mild ADPKD), 47 y (moderate ADPKD)<br>-Similar baseline BP (hypertensive patients could be included with controlled BP < 140/90)<br>-Significant decrease in iothalamate clearance and renal blood flow (PAH & MRI) from control to mild ADPKD to moderate ADPKD<br><br>After 4 week simvastatin:<br>-All groups showed significant decrease in LDL cholesterol<br>-Statistically significant but clinically small effect on renal blood flow in ADPKD patients after treatment<br>--mild ADPKD RBF by PAH pre 717 ± 190 vs post 669 ± 194 mL/min (p=0.05)<br>--mod ADPKD RBF by PAH pre 370 ± 89 vs post 378 ± 94 mL/min (p<0.0001)	Serious (small sample size with short duration of study)	Not serious	Not serious? (Contradicts vanDijk findings but this population older with more renal dysfunction): could statin effect be more impressive in younger patients / less advanced disease?	Serious (MRI data)	Limited MRI study of RBF (2/11 controls, 2/11 mild ADPKD, 3/10 mod ADPKD not studied due to technical difficulties)<br><br>Hydrophilic vs lipophilic statin?	7
Effect of simvastatin on renal function in autosomal dominant polycystic kidney disease<br><br>PMID 11682660	van Dijk, M.A.	Nephrol Dial Transplant 16:2152-7	2001	Double blind randomized placebo-controlled crossover	10 untreated ADPKD adult patients with normal serum cholesterol and Cockroft Gault eGFR > 50 mL/min including 3 smokers<br><br>Mean age 39 ± 4 yr	After 4 weeks simvastatin 40 mg daily vs. placebo with study periods separated by 4 week washout:<br>-GFR by inulin clearance<br>- ERPF by PAH clearance<br>-Forearm vascular reactivity by intra-arterial cumulative dose infusion of acetylcholine and sodium nitroprusside	Simvastatin treatment for 4 weeks associated with:<br>-Increase in GFR (132 ± 6 vs. 124 ± 4 mL/min, p<0.05)<br>-Increase in estimated renal plasma flow (619 ± 67 vs. 494 ± 30 mL/min, p<0.05)<br>-Enhanced vasodilator response to acetylcholine with response to sodium nitroprusside unchanged<br>-Reduced serum cholesterol levels<br>-No effect on serum CK<br><br>Results included 9 patients for GFR/ estimated renal plasma flow measurements and 6 patients for vasodilator response due to technical issues	Serious (short term study with small sample size and technical limitations on data obtained)	Not serious	Serious for kidney effects; not serious for endothelial effects	Serious (limited success in obtaining data from study subjects due to technical issues)	subsequent studies by others showing limited reproducibility of kidney effect	6

Title	First Author	Journal	Year	Study design	Nº & type of patients	Outcome measure(s)	Results	Risk of bias	Indirectness	Inconsistency	Imprecision	Other con-siderations	Importance (1-9)
Effect of statin therapy on the progression of autosomal dominant polycystic kidney disease. A secondary analysis of the HALT PKD trials.<br><br>PMID: 28460625	Brosnahan, G.	Curr Hypertens Rev 2017 Apr 27	2017	Post hoc analysis of adult HALT-PKD clinical trials	HALT-PKD A and B trials<br><br>Compared outcomes between participants who never used statins versus used statins for at least 3 years (self-reported as concomitant med in 6-month intervals; not necessarily consecutive use)	Effect of statin on: Study A: percent change in TKV and total liver volume and rate of decline of eGFR<br><br>Study B: time to composite endpoint of death, ESRD, or 50% decline in eGFR	Due to imbalanced groups, applied propensity score model with inverse probability of treatment weighting (IPTW) to create cohorts well balanced on baseline characteristics<br>HALT Study A:<br>59/558 (10%) participants on statin (male and older)<br>Baseline statin vs no statin: age 41 vs 36 year [p<0.0001], htTKV 786 ± 434 vs 680 ± 401 mL/m [p=0.06], CKD EPI GFR 84 ± 15 vs 93 ± 18 mL/min/1.73m² [p=0.0004]<br>Excluded 83 total from IPTW<br>➔ No difference in between statin and no statin in change in TKV (6.5% per year statin and 6.2% per year no statin)<br>HALT Study B:<br>118/486 (24%) participants on statin (male and older)<br>Baseline statin vs no statin: age 52 vs 47 y [p<0.0001], CKD-EPI GFR 48 ± 11 vs 49 ± 12 mL/min/1.73m² [p=NS]<br>Excluded 58 total from IPTW<br>➔ No difference in composite endpoint of death, ESRD, 50% decline in eGFR	Very serious (study not designed to look at statin use)	Very serious	Serious	Serious	Clinical trial was not designed to assess effect of statin<br><br>Statin use, type, dose, indications not randomized/controlled; cholesterol not followed (? Compliance)<br><br>Limited number of patients with preserved eGFR received statins<br><br>Advanced kidney disease in Study B	4
**Effects on surrogate markers**													
Improvement of endothelial dysfunction with simvastatin in patients with autosomal dominant polycystic kidney disease<br><br>PMID 17365910	Namli, S.	Ren Fail 29:55-9	2007	Prospective open label	16 ADPKD (Ravine criteria) adult patients with Cockroft-Gault clearance > 80 mL/min/1.73m² including 9 smokers and 7 on BP meds<br><br>Mean age 39 ± 11 years	Endothelial-dependent dilatation by brachial artery US, serum IL6 and high sensitivity CRP at baseline and 6 months after simvastatin 40 mg daily	Simvastatin treatment associated with:<br>Increased endothelial-dependent dilatation:<br>-baseline (pre statin) 11.3 ± 6.9%<br>-one month Rx 14.6 ± 4.6% (p=0.016)<br>-six month Rx 18.9 ± 7.5% (p=0.011 vs baseline)<br><br>Decreased total and LDL cholesterol<br>Decrease in IL6 from 21.6 ± 21.7 to 9.1 ± 3.5 pg/mL (p=0.002)<br><br>No change in high sensitivity CRP	Unlikely	Not serious	Serious (varied population with BP and smoking status)	Not serious	Small participant numbers limit ability to assess effect of hypertension, BP meds, smoking	6
Effect of pravastatin on kidney function and urinary protein excretion in autosomal dominant polycystic kidney disease<br><br>PMID 20034362	Fassett, R.G.	Scand J Urol Nephrol 44:56-61	2010	Prospective randomized open label	49 adult ADPKD (ultrasound + family history)<br>All levels of kidney function<br>Randomized to pravastatin 20 mg/d or no treatment for 2 years<br><br>Mean age 53 ± 15 y (statin); 49 ± 12 y (control)	Quarterly assessment over 2 years:<br><br>Primary outcome: kidney function by MDRD equation<br><br>Secondary outcomes: urinary protein excretion and creatinine clearance by 24h urine	No significant change in markers of kidney function or urinary protein excretion over 2-year study period despite significant decrease in serum total cholesterol in statin-treated patients	Serious (all levels of kidney function assessed)	Serious (low dose of pravastatin 20 mg/d could have limited effect)	Not serious	Serious (more sensitive method to assess primary outcome?)	Limited sensitivity of eGFR markers but clinically obtainable<br><br>Small sample size but larger than other studies<br><br>Large variability in baseline protein excretion	5

Table S8b Studies reporting rates of side-effects of statins in children

Title	First Author	Journal	Year	Study design	Nº & type of patients exposed	Reported side effects	Risk of bias	Indirectness	Inconsistency	Imprecision	Other con-siderations	Importance (1-9)
**In ADPKD**												
Effect of pravastatin on total kidney volume, left ventricular mass index, and microalbuminuria in pediatric autosomal dominant polycystic kidney disease<br>PMID 24721893	Cadnapaphornchai, M.A.	Clin J Am Soc Nephrol 9:889-96	2014	Randomized prospective double-blind placebo controlled phase 3 clinical trial	110 pediatric and young adults with ADPKD 8-22 y with Schwartz eGFR > 80 mL/min/1.73m^2 treated with lisinopril targeting 50-75th percentile BP<br><br>-Statin enrolled n=56, completed n=49<br>-Placebo enrolled n=54, completed n=42	-No participants discontinued statin/placebo due to side effects<br><br>-No significant differences between study groups for serum CK, AST, ALT during study<br>--1 participant in each study group with elevated AST > 2XULN, both obese and demonstrated to have NAFLD<br><br>-3 pregnancies<br>-1 statin/Lisinopril discontinued meds at 4 weeks gestation with subsequent delivery of healthy term infant<br>-1 placebo participant on no medication subsequently delivered a healthy term infant<br>-1 statin/Lisinopril participant with > 1 year noncompliance resumed meds 2 weeks before final study visit, with positive pregnancy test 1 week later leading to discontinuation of medications at that time; however a local ultrasound did not show an embryo and she was believed to have miscarrried	Not serious	Not serious	Not serious	Not serious (largest pediatric cohort)	High drop out rate but not related to adverse effects	9
**In hypercholesterolemia**												
Efficacy and safety of pitavastatin in children and adolescents at high future cardiovascular risk<br><br>PMID 26059337	Braamskamp, M.J.A.M.	J Pediatr 167:338-43	2015	Randomized double-blind placebo-controlled study of pitavastatin (1, 2, 4 mg/d) vs placebo for children with hyperlipidemia followed by 52 week open label	106 participants ages 6-17y with hyperlipidemia (most familial) and additional CV risk factor treated for 12-week period targeting LDL-C < 110 mg/dL<br><br>Safety labs every 4 weeks	<u>Double blind phase:</u> Total adverse events in 16 of 106 subjects (15%) including abdominal pain (4.7%), headache (2.8%), abdominal discomfort (1.9%), all similar between treatment groups. No patients with CK > 5X ULN or LFT > 3X ULN<br><u>Open label phase:</u> Total adverse events in 67% of subjects with drug related adverse events affecting 10 subjects (8.9%): musculoskeletal (n=3), nervous system disorders (n=3), rash (1). Routine lab monitoring showed 1 patient with ALT > 3X ULN possibly related to drug; 1 patient with AST > 3X ULN; No patients with CK > 5X ULN<br><br>No apparent impact on growth or pubertal development	Not serious	Not serious	Not serious	Not serious		6
A randomized, double blind, placebo-controlled pilot trial of the safety and efficacy of atorvastatin in children with elevated low-density lipoprotein cholesterol (LDL-C) and type 1 diabetes<br><br>PMID 25418907	Canas, J.A.	Pediatr Diabetes 16:79-89	2015	Randomized double-blind placebo controlled to assess effect of atorvastatin on LDL-C in children with type 1 diabetes	42 children with type 1 diabetes, mean age 15 ± 3 years, mean diabetes duration 6.8 ± 0.5 y with mean LDL-C 124 ± 4 mg/dL randomized to atorvastatin (initial dose 10 mg/d increasing to 20 mg/d if 3 month LDL-C remained > 100 mg/dL) or placebo for 6 months<br><br>Assessed serum CK and LFT's at baseline, 1, 3, 6 months	One participant had asymptomatic elevation of CK to > 10X ULN on atorvastatin 10 mg/d which normalized with atorvastatin discontinuation<br><br>4/21 statin and 4/21 placebo had musculoskeletal complaints, none of which correlated with serum CK nor required discontinuation of medication<br><br>Atorvastatin significantly lowered LDL-C, apoB, and atherogenic lipoprotein subparticles without worsening insulin resistance as assessed by insulin sensitivity score using SEARCH ISS model	Not serious	Not serious	Not serious	Serious (short term)	Short term study cannot assess long-term effects<br><br>Small sample size	6

Title	First Author	Journal	Year	Study design	Nº & type of patients exposed	Reported side effects	Risk of bias	Indirectness	Inconsistency	Imprecision	Other con-siderations	Importance (1-9)
A 3-year study of atorvastatin in children and adolescents with heterozygous familial hypercholesterolemia<br><br>PMID 27678432	Langslet, G.	J Clin Lipidol 10:1153-62	2016	Prospective open label study of atorvastatin in children 6-15y with heterozygous familial hypercholesterolemia	272 participants initiated atorvastatin at 5-10 mg/d increasing up to 80 mg/d based on LDL-C studied for 3 years	No adverse effect on growth or maturation<br><br>6 subjects (2%) discontinued because of adverse events (Ewing sarcoma, increased bilirubin, abdominal pain/fatigue/headache, myalgia, eosinophilia, intravascular endothelial papillary hyperplasia)<br><br>No participants with LFT > 3xULN; 23 subjects (8.6%) with CK > 2xULN including 1 participant with CK > 10x ULN after "hard physical exercise"	Not serious	Not serious	Not serious	Not serious	76% completion rate with dropouts largely related to no longer willing to participate in study or "low LDL-C"	5
Long-term follow-up of statin treatment in a cohort of children with familial hypercholesterolemia: efficacy and tolerability<br><br>PMID 21692550	Carreau, V.	Paediatr Drugs 13:267-75	2011	Retrospective chart review of hypercholesterolemic children referred to 2 French tertiary care centers and treated with pravastatin	185 pravastatin-treated children with familial hypercholesterolemia (168 with genetic diagnosis) and cholesterol > 300 mg/dL treated with pravastatin for mean 26 months (range 3-84 months) starting dose 10 mg increased up to 40 mg depending on age/response	13% (n=24) reported any side effects including 4 children with musculoskeletal complaints thought to possibly be related to statin treatment<br><br>Asymptomatic elevation in CK < 2X ULN in 8 usually after playing sports which normalized spontaneously	Serious (chart review)	Not serious	Not serious	Serious	Small study with no control group	5
**In fetus'**												
Safety and pharmacokinetics of pravastatin used for the prevention of preeclampsia in high-risk pregnant women: a pilot randomized controlled trial<br><br>PMID 26723196	Costantine, M.M.	Am J Obstet Gynecol 214:720.e1-720.e17	2016	Multicenter double-blind placebo-controlled randomized trial of pravastatin 10 mg/d vs placebo in pregnancy starting at 12-17 weeks gestation	10 women statin and 10 placebo	Umbilical cord cholesterol concentration (when assessed) and infant birthweight not different between groups<br><br>Congenital anomalies:<br>1 statin (hypospadias, aortic coarctation)<br>1 placebo (polydactyly ? familial, ventriculomegaly)	Serious (very small study with limited power)	Serious		Serious (very small study at recommendation of FDA for pilot trial)	Statin low dose and started in 2nd trimester<br><br>Pravastatin chosen as hydrophilic statin anticipated to have less transplacental transfer<br><br>No long-term follow up of infants although this is planned	5
**Important trials in adults**												
Cardiovascular benefits and diabetes risks of statin therapy in primary prevention: an analysis from the JUPITER trial<br><br>PMID 22883507	Ridker, P.M.	Lancet. 380:565-71.	2012	Assessment of cardiovascular benefits vs diabetes risk of rosuvastatin therapy in randomized double blind placebo controlled JUPITER trial in adults	17603 adult participants without previous cardiovascular disease or diabetes assigned to 20 mg/d rosuvastatin or placebo followed for 5 years for primary cardiovascular endpoints stratified for analysis by diabetes risk factors including metabolic syndrome, impaired fasting glucose, BMI > 30 kg/m2, A1C > 6%	Individuals with one or more diabetes risk factors:<br>Statin allocation was associated with 39% reduction in primary endpoint and 28% increase in diabetes (HR 1.28 [1.07-1.54] p=0.01) i.e. a total of 134 vascular events/deaths were avoided for every 54 new cases of diabetes diagnosed.<br><br>Individuals with no major diabetes risk factors:<br>Statin allocation associated with 52% reduction in primary endpoint and 22% reduction in total mortality and no increase in diabetes (HR 0.99 [0.45-2.21] p=0.99) i.e. a total of 86 vascular events/deaths were avoided with no new cases diabetes diagnosed with statin therapy	Not serious	Not serious (original trial not designed to answer statin diabetes question but large adult study)	Not serious	Not serious		7

Title	First Author	Journal	Year	Study design	Nº & type of patients exposed	Reported side effects	Risk of bias	Indirectness	Inconsistency	Imprecision	Other con-siderations	Importance (1-9)
Statin use and risk of developing diabetes: results from the Diabetes Prevention Program<br><br>PMID 29081977	Crandall, J.P.	BMJ Open Diab Res Care doi10.1136/bmjdrc-2017-000438	2017	Analysis of statin-diabetes association using data from Diabetes Prevention Program which studied a cohort of overweight/ obese adults at high risk of diabetes followed specifically for incident diabetes	3234 adult participants (20% were 60 years or older) who were overweight/obese with both fasting plasma glucose between 95-125 mg/dL and impaired glucose tolerance test randomized to intensive lifestyle intervention, metformin or placebo for mean follow up 3.2 years then continued in open label lifestyle intervention with metformin group continuing on metformin<br><br>Statin use determined by self-report of concomitant medications. Cumulative statin use defined as number of semiannual visits with reported use.	Statin use increased from 4% at baseline to > 30% over 10 year follow up period<br><br>Mean age reporting statin use 53 years<br><br>Statin users had higher baseline LDL-C and triglycerides, fasting glucose & HbA1c and lower insulinogenic index and more common history of CVD and hypertension than non statin users<br><br>Statin use was associated with greater diabetes risk regardless of treatment group (pooled HR 1.36 with 95% CI 1.17-1.58)	Serious	Serious	Not serious	Very serious (statin treatment was not randomized or protocol driven, statin dose not available)	Adults with comorbid conditions warranting statin therapy but does not clarify indications for statin<br><br>Baseline parameters suggest high risk of diabetes in those who eventually received statin treatment	6
The effect of lipophilicity and dose on the frequency of statin-associated muscle symptoms: A systematic review and meta-analysis<br><br>PMID 28943224	Irwin, J.C.	Pharmacol Res. 2017 Sep 21. [Epub ahead of print]	2017	Meta-analysis of adult trials	135 adult RCT using statin monotherapy vs placebo/standard care examining relationship between statin lipophilicity/dose and adverse muscle symptoms (pain, weakness, myositis, myalgia, myopathy, rhabdomyolysis)<br>-6 crossover<br>-129 parallel<br>-after quality assessment, total of 121 studies included	Total 192977 participants (100431 statin, 92546 placebo or usual care)<br>-Adverse muscle symptoms reported in 8775 statin, 7885 placebo<br><br>Statins associated with increased risk of adverse muscle symptoms (RR 1.05 with 95% CI 1.014-1.089, p=0.007)<br>-Mild increase in RR appeared to relate to inclusion of RCT which recruited participants with prior history of statin intolerance<br><br>No relationship between lipophilicity or dose and symptoms	Serious (included those with prior history of statin intolerance)	Not serious meta-analysis of studies designed primarily to look at cardiovascular outcomes, not adverse events;	Not serious	Meta-analysis		5

<u>Abbreviations:</u> CK, creatinine kinase; EDD, endothelium dependent dilation; ERPF, effective renal plasma flow; FH, family history; HETE, hydroxyeicosatetraenoic acid; HODE, hydroxyoctadecadienoic acid; HtTKV, height adjusted total kidney volume; LVMI, left ventricular mass index; NAFLD, nonalcoholic fatty liver disease; RR relative risk; UAE, urinary albumin excretion; ULN, upper limit of normal

Table S9 mTOR inhibitors for delaying progression of ADPKD

Title	First Author	Journal	Year	Study design	Nº & type of patients	Outcome measure(s)	Results	Risk of bias	Indirectness	Inconsistency	Imprecision	Other considerations	Importance (1-9)
**Randomized trials in adults**													
Everolimus in patients with autosomal dominant polycystic kidney disease<br><br>PMID: 20581392	Walz, G.	N Eng J Med; 363:830-40	2010	Randomized double blind, placebo controlled	431 adult patients with ADPKD and an eGFR of 30-89 ml/min/1.73 m^2 or eGFR >90 ml/min/1.73 m^2 and single kidney volume > 1000 ml	Change in TKV (MRI), Cyst volume (MRI) and eGFR at 12 and 24 months	Change in TKV at 1 yr: 102 ml (Ev) vs 157 ml (Plac) (p=0.02)<br>Change in TKV at 2 yr: 230 ml (Ev) vs 301 ml (Plac) (p=0.06)<br>Change in CV at 1 yr: 76ml (Ev) vs 98 ml (Plac) (p=0.27)<br>Change in CV at 2 yr: 181 ml (Ev) vs 215 ml (Plac) (p=0.28)<br><br>Change in eGFR at 2 yr: -8.9 ml/min/1.73 m^2 (Ev) vs -7.7 ml/min/1.73 m^2 (Plac) (p=0.15)	Not serious	Not serious	Not serious	Not serious	Use of Everolimus associated with high rate of side effects	9
Rapamycin for treatment of type I autosomal dominant polycystic kidney disease (RAPYD-study):a randomized, controlled study<br><br>PMID: 22785114	Stallone, G.	Nephrol Dial Transplant 27:3560-3567	2012	Prospective, open label, randomized, controlled	55 ADPKD patients (18-65yrs; GFR 40-80 ml/min/1,73m^2)<br><u>Group A:</u> ramipril<br><u>Group B:</u> ramipril + high-dose rapamycin<br><u>Group C:</u> ramipril + low-dose rapamycin	Change in TKV, cyst volume and eGFR (MDRD formula) after 24 months	<u>Change in TKV after 24 months:</u><br>Group A: 1869±668 → 1905±650 ml (p=0.03 to baseline)<br>Group B: 1493±672 → 1508±674 ml (p=0.02 to baseline)<br>Group C: 1712±634 → 1726±628 ml<br><br><u>Change in cyst volume after 24 months:</u><br>Group A: 166±49 → 169±88 ml (p<0.001 to baseline)<br>Group B: 140±95 → 125±93 ml (p<0.0001 to baseline)<br>Group C: 157±108 → 141±98 ml (p<0.0001 to baseline)<br><br><u>Change in eGFR after 24 months:</u><br>Group A: 62.7±13.6 → 59.1±15.1 ml/min/1.73m^2 (p=0.01 to BL)<br>Group B: 61.4±17.4 → 65.9±24.0 ml/min/1.73m^2<br>Group C: 62.1±13.9 → 62.9±26.2 ml/min/1.73m^2	Serious<br><br>(kidney volumes not corrected for variables such as age or gender, as proposed by the CRISP study)	Not serious	Not serious	Not serious	Side effects in high–dose vs low-dose: hyperlipidemia (47% vs. 11%), proteinuria (0.65 vs. 0.25 g/24h), oral ulcers (31.6% vs. 11.1%) and anemia (10.5% vs. 0%), all p <0.01	8
Sirolimus and kidney growth in autosomal dominant polycystic kidney disease<br><br>PMID: 20581391	Serra, A.L.	N Eng J Med; 363:820-9	2010	Open-label, randomized controlled	100 patients with ADPKD (18-40 years) eGFR> 70 ml/min (Cockcroft-Gault)	Change in TKV (MRI), eGFR and urinary albumin excretion at 18 months	Change in TKV at 18 months: 99 ml (Sir) vs 97 ml (Control) (p=0.26)<br>eGFR at 18 months: 0. ml (Sir) vs -3.5 ml (Control) (p=0.07)<br>Urinary albumin excretion at 18 months: +1.1 mg/l (Sir) vs 0.0 mg/l (Control)<br>Oral mucositis 82% Sir vs 14% control	Not serious. 18 months possibly too short to see effects	Not serious	Not serious	Not serious	Sirolimus dose 25% lower than the intended 2 mg due to side effects	8
Everolimus does not further reduce polycystic liver volume when added to long acting octreotide: results from a randomized controlled trial.<br><br>PMID: 23499726	Chrispijn M.	J of Hepatol. 59(1):153–9	2013	Randomized controlled trial<br><br>(ELATE trial)	44 patients (29 ADPKD/15 ADPLD) >18 years, liver volume > 4000 ml receiving 48 weeks of octreotide monotherapy or octreotide plus everolimus	Change in total liver volume (TLV) was primary outcome.<br><br>Change in TKV secondary outcome.	<u>Change in TLV after 48 weeks</u>: (p=0.73 between groups)<br>Octreotide mono (n=23) -3.5 ± 5.2% (p<0.01 to baseline)<br>Octreotide + Eve (n=21) -3.8 ± 4.7% (p<0.01 to baseline)<br><br><u>Change in TKV after 48 weeks (n=12)</u>: (p=1.0 between groups)<br>Octreotide mono (n=6) 798 ml → 811 ml (p=ns to baseline)<br>Octreotide + Eve (n=6): 623 ml → 602ml (p=ns to baseline)	serious	Not serious	Not serious	Not serious	1 suspected case of cyst infection due to octreotide. 89% female.	7

Title	First Author	Journal	Year	Study design	Nº & type of patients	Outcome measure(s)	Results	Risk of bias	Indirectness	Inconsistency	Imprecision	Other considerations	Importance (1-9)
Sirolimus therapy to halt the progression in ADPKD<br><br>PMID: 20466742	Perico, N.	J Am Soc Nephrol 21(6):1031-1040	2010	Randomized crossover study<br><br>(SIRENA study)	15 ADPKD patients > 18 yrs and eGFR > 40 ml/min/1.3m^2<br><br>Sirolimus followed by conventional therapy (n=7); Convent. Therapy followed by Sirolimus (n=8)	Change in TKV/TCV and GFR after 6 months	Change in TKV after 6 months: (p=0.45)<br>Sirolimus: +46±81 ml (+2.2%)<br>Conventional: +70 ±72 ml (+3.7%)<br>Change in TCV after 6 months: (p=0.023)<br>Sirolimus: 4±52 ml (+0.3%)<br>Conventional: 55 ±75 ml (+5.5%)<br>Change in parenchymal volume after 6 months: (p=0.094)<br>Sirolimus: +26±30 ml (+8.7%)<br>Conventional: -2 ±20 ml (-0.4%)<br>GFR: no changes in measured GFR observed in both treatment periods (Sir: 75.8 →73.4; convent.73.7→74.3 ml/min/1.73m^2)	Serious: Small short study, carryover effects in group staring with Sirolimus followed by conventional therapy	Not serious	Not serious	Not serious	Increase in albuminuria in Sirolimus group	7
Sirolimus reduces polycystic liver volume in ADPKD patients<br><br>PMID:18199797	Quian, Q.	J Am Soc Nephrol 19:631-638	2008	Prospective single center randomized trial	16 adult ADPKD patients undergoing kidney transplantation Sirolimus (n=7) Tacrolimus (n=9)	Change in TKV (primary endpoint) and liver volume (retrospectively assessed 11 months before kidney Tx (1.) and at least 7 months after kidney Tx (2.)	Change in liver volume: (*P* = 0.009)<br>Sirolimus: −11.85% ± 0.03<br>Tacrolimus: +14.13 ± 0.09<br><br>Change in total kidney volume: (*P* = 0.38)<br>Sirolimus: -14.76 ± 0.08% (right kidney);<br>-15.03 ± 0.08% (left kidney)<br>Tacrolimus: -10.9 ± 0.06% (right kidney);<br>-9.0 ± 0.06% (left kidney)	Not serious	serious	Not serious	Not seroius	Liver volume was no endpoint of the study and was assessed in an additional retrospective study on the same cohort	7
**Non-randomized studies**													
Therapeutic mTOR Inhibition in Autosomal Dominant Polycystic Kidney Disease: What is the appropriate serum level?<br><br>PMID: 20642692	Canaud, G.	Am J Tranplant 10(7):1701-1706.	2010	Case-control Report	2 patients receiving a transplant kidney (Tx) from the same ADPKD donor, treated with sirolimus (Sir+) and without (Sir-).	Change in TKV/TCV between 4 and 5 years after Tx	TKV/TCV at 4 yrs after Tx: 255/23.5 (Sir-) vs 246/21.4 (Sir+)<br>TKV/TCV at 5 yrs after Tx: +9.8%/13.6% (Sir-) vs +12.9%/17.2% (Sir+)<br><br>No difference in eGFR decrease 4 yrs after Tx<br><br>Despite complete mTOR inhibition in monocytes, no difference in mTOR activity in kidney tissue in Sir+ and Sir-.	Serious	Serious	Not serious	Not serious		5
The mTOR pathway is regulated by polycystin-1, and its inhibition reverses renal cystogenesis in polycystic kidney disease<br><br>PMID: 16567633	Shillingford J.M.	Proc NatlAad Sci USA; 103:5466-5471	2006	Retrospective single center study	7 ADPKD patients receiving renal transplant: main immunosuppressive agent Rapamycin (Rap; n=4) or Cyclosporine (CSA; n=3)	TKV (CT) assessed 11 months before - 5 months after Tx (1.) and at least 11 months after Tx (2.)	TKV of remaining native kidneys: (p=0.03 between groups)<br>Rapamycin: -24.8% ± 9.7% over 24 months (p<0.001 to pre-Tx)<br>Control (CSA): -8.6% ± 11.2% over 40 months (p=ns to pre Tx).	serious	serious	serious	serious	Small number of patients and with quite different characteristics Inconsistent findings with prospective clinical trials	4

Abbreviations: ADPKD = autosomal dominant polycystic kidney disease; CV= Cyst volume; mTOR = mammalian target of Rapamycin; TKV= total kidney volume; Tx= transplantation

Tables S10 somatostatin analogues for ADPKD

Table S10a somatostatin analogues for delaying progression of kidney disease in ADPKD

Title	First Author	Journal	Year	Study design	Nº & type of patients	Outcome measure(s)	Results	Risk of bias	Indirectness	Inconsistency	Imprecision	Other considerations	Importance (1-9)
**Randomized trials**													
Effect of Lanreotide on Kidney Function in Patients With Autosomal Dominant Polycystic Kidney DiseaseThe DIPAK 1 Randomized Clinical Trial<br><br>doi:10.1001/jama.2018.15870	Meijer, E	JAMA, published online 25 Oct	2018	Randomized controlled, open-label trial	153 ADPKD patients with lanreotide vs 152 ADPKD patients with standard care	Change in slope of eGFR loss	Annual rate of eGFR decline:<br>Lanreotide −3.53 mL/min/1.73 m2 per year<br>control group was vs −3.46 mL/min/1.73 m2 per year (difference, −0.08; [95% CI, −0.71 to 0.56]; p = 0.81)	Not serious	Not serious	Not serious	Not serious	No children included.	9
Effect of longacting somatostatin analogue on kidney and cyst growth in autosomal dominant polycystic kidney disease (ALADIN): a randomised, placebo-controlled, multicentre trial.<br><br>PMID: 23972263	Caroli, A.	Lancet 382(9903): 1485-95	2013	Randomized placebo controlled, single-blind, multicenter trial<br><br>(ALADIN trial)	79 ADPKD patients > 18 yrs; eGFR > 40 ml/min/1.73m²	Effect long acting release (LAR)-octreotide treatment on kidney and cyst growth (MRI at 1 and 3 years) and renal function decline	Change in TKV at 1 year: (p=0.032)<br>LAR (n=38): 46.2 ± 18.2 ml<br>Placebo (n=37): 143.7 ± 26.0 ml<br>Change in TKV at 3 years: (p=0.25)<br>LAR (n=35): 220.1 ± 49.1 ml<br>Placebo (n=35): 454.3 ± 80.8 ml<br>Change in TCV at 1 year: (p=0.017)<br>LAR (n=38): 33.0 ± 14.7 ml<br>Placebo (n=37): 108.5 ± 18.3 ml<br>Change in TCV at 3 years: (p=0.11)<br>LAR (n=35): 183.8 ± 41.7 ml<br>Placebo (n=35): 394.7 ± 62.9 ml<br>Change in eGFR at 1. year: (p=ns)<br>LAR (n=34): 88.7 → 77.86 ml/min/1.73 m²<br>Placebo (n=32): 77.77 → 72.16 ml/min/1.73 m²<br>Change in eGFR at 3. year: (p=ns)<br>LAR (n=36): 88.7 → 76.33 ml/min/1.73 m²<br>Placebo (n=31): 77.77 → 64.64 ml/min/1.73 m²	Not serious	Not serious	Not serious	Not serious	4 cases of probably treatment-related cholelithiasis or acute cholecystitis occurred in the octreotide-LAR group. Otherwise well tolerated.	8
Randomized clinical trial of long-acting somatostatin for autosomal dominant polycystic kidney and liver disease<br><br>PMID: 20431041	Hogan, M.C.	J Am Soc Nephrol 21(6): 1052–1061	2010	Randomized, double-blind, placebo-controlled trial (2:1)<br><br>(MAYO trial)	42 patients (34 ADPKD/ 8 ADPLD) ≥18 years of age; >4000 mL hepatic volume) receiving Octreotide-LAR or placebo for 1 year	Change in TKV (MRI)<br><br>Change in GFR	Change in TKV after 12 months: (p=0.045)<br>LAR (n=28): +0.25 ±7.53%<br>Placebo (n=14): +8.61 ±10.07%<br>Change in GFR over 12 months: (p=0.98)<br>LAR (n=21): 68.1 → 64.6 ml/min/1.73m² = -5.1%<br>Placebo (n=9): 70.1 → 65.7 ml/min/1.73m² = -7.2%<br>Change quality of life over 12 months: (p=0.045)<br>LAR (n=28): +0.25 ±7.53%<br>Placebo (n=14): +8.61 ±10.07%<br>Patients on LAR reported an improved perception of bodily pain and physical activity	Not serious	Not serious	Not serious	Not serious	No serious side effects.	9

Title	First Author	Journal	Year	Study design	Nº & type of patients	Outcome measure(s)	Results	Risk of bias	Indirectness	Inconsistency	Imprecision	Other con-siderations	Importance (1-9)
Lanreotide reduces the volume of polycystic liver: a randomized, double-blind, placebo-controlled trial<br><br>PMID: 19646443	Keimpema, L.	Gastroenterology. 137(5):1661–8 e1–2	2009	Randomized, double-blind, placebo-controlled trial<br><br>(LOCKCYST Trial)	54 patients (n=32 ADPKD and n=22 ADPLD, aged >18 years; >20 liver cysts) receiving either lanreotide or placebo for 6 months	TKV (CT) at 0 and 24 weeks	Change in TLV after 6 months: (p<0.01)<br>LAR (n=27): 4606 (547-8665) → 4471(542-8401) ml, -2.9%<br>Placebo (n=27): 4689 (613-8765) → 4896 (739-9035) ml, +1.6%<br><br>Change in TKV after 6 months: (p=0.02)<br>LAR (n=27): 1000 (39-2039) → 983(62-2028) ml -1.5%<br>Placebo (n=27): 1115 (519-2748) → 1165 (541-2870) ml + 1.6%<br><br>No significant change of serum creatinine in both groups	Not serious	Not serious	Not serious	Not serious	No serious side effects;<br><br>Short duration of treatment	8
Safety and efficacy of long-acting somatostatin treatment in autosomal-dominant polycystic kidney disease.<br><br>PMID: 15954910	Ruggenenti, P.	Kidney Int 68(1):206–16.	2005	Randomized, crossover, placebo-controlled trial	12 ADPKD patients (> 18 yrs; Serum creatinine >1.2 mg/dl and <3 mg/dl) receiving LAR octreotide for 6 months followed by 6 months placebo	Change in TKV (CT) after 6 month of octreotide-LAR or placebo<br><br>eGFR after 6 month of octreotide-LAR or placebo	Change in TKV after 6 months: (p<0.01)<br>LAR (n=12): +71 ±107 ml<br>Placebo (n=12): +162±114 ml<br><br>Change in parenchymal volume after 6 months:<br>LAR (n=12): -10 ±24 ml<br>Placebo (n=12): +9±22 ml<br><br>eGFR before and after 6 months: (p=ns)<br>LAR (n=12): 59.5±25.2→ 54.0±23.6 ml/min/1.73m^2<br>Placebo (n=12): 57.9±22.4 → 57.7±25.7 ml/min/1.73m^2	Not serious. Small study.	Not serious	Not serious	Not serious	No serious side effects	8
**Non-randomized studies**													
Somatostatin analog therapy for severe polycystic liver disease: results after 2 years<br><br>PMID: 22773240	Hogan, M.C.	Nephrol Dial Transplant 27:3532-3539	2012	Open label extension of MAYO trial (Hogan 2010)	41 patients (34 ADPKD/ 8 ADPLD), aged ≥18 years; >4000 mL hepatic volume).<br>(O-O): 1st and 2nd year octreotride<br>(P-O): 1st year placebo, 2nd year octreotide	Change in TKV (MRI)<br><br>Change in GFR	Change in TKV in 2nd year:<br>O →O (n=19): +0.42 ±7.61% (1st year) (p=0.81)<br>+6.49±7.08% (2nd year) (p=0.0008)<br>P → O (n=8): +8.61 ±10.07% (1st year) (p=0.046)<br>+0.41±9.45% (2nd year) (p=0.90)<br>Change in GFR in 2nd year:<br>O →O (n=21): -5.1±15.5% (1st year) (p=0.15)<br>-7.9±13.8% (2nd year) (p=0.019)<br>P → O (n=9): -7.2±13.2% (1st year) (p=0.14)<br>-6.3±13% (2nd year) (p=0.19)	Not serious	Not serious	Not serious	Not serious	One hepatic cyst infection associated with octreotide	8
Alkaline phosphatase predicts response in polycystic liver disease during somatostatin analogue therapy: a pooled analysis<br><br>PMID: 26481454	Gevers, T.J.	Liver Int. 36(4):595-602.	2016	Pooled analysis of 4 clinical trials (LOCKCYST (n=51), ELATE (n=32); RESOLVE (n=28), Mayo (n=42))	153 patients with polycystic liver disease (100 ADPKD)	Prediction of kidney volume reduction	ADPKD subgroup (n=100): elevated alkaline phosphatase predicted liver volume reduction (−3.2%, P = 0.03) but did not predict kidney volume reduction (+0.1%, P = 0.97).	Not serious	Not serious	Not serious	Not serious	Post-hoc analysis. Overlap with previous papers.	7

Title	First Author	Journal	Year	Study design	Nº & type of patients	Outcome measure(s)	Results	Risk of bias	Indirectness	Inconsistency	Imprecision	Other con-siderations	Importance (1-9)
Efficacy of 4 years of octreotide long-acting release therapy in patients with severe polycystic liver disease.<br><br>PMID: 26166166	Hogan, M.C.	Mayo Clin Proc.; 90(8):1030-7.	2015	Open label observational extension of Hogan MC. 2012	21 ADPKD patients formerly enrolled in a (1 year RCT (Hogan 2010 (13 initially on LAR, 8 initially on placebo) and 2 year open-label-extension (all on LAR). Re-enrolment on another 2 years of LAR-octreotide after being mean pause of 8.3 months	Change in TKV (MRI)<br><br>Change in GFR	Change in TKV on LAR in 1st trial: (p=0.5)<br>LAR (n=15): 777 → 792 ml +4.0±6.8%<br><br>Change in TKV off LAR: (p=0.2)<br>LAR (n=15): 819 → 862 ml +5.5±7.7%<br><br>Change in TKV on LAR in 2 year extension : (p=0.02)<br>LAR (n=15): 862 → 879 ml +0.7±13.6%<br><br>No significant loss of eGFR (58.2 →54.5 ml/min/1.73 m^2 over 4 year period) compared to placebo group	Serious	Not serious	Not serious	Not serious		7
The long-term outcome of patients with polycystic liver disease treated with lanreotide<br><br>PMID: 22111942	Chrispijn, M.	Aliment Pharmacol Ther 35:266-274	2012	Open-label observational extension trial of the LOCKCYST trial	41 patients (ADPKD n=25; ADPLD n=16), aged >18 years; >20 liver cysts; on lanreotide for 12 months in total	TKV (CT) at 0 and 12 months	Change in TKV after 12 months: (p=0.33)<br>LAR (n=25): 1156 (735-1751) → 1114(666-1670) ml<br>-1.0%	Not serious	Not serious	Not serious	Not serious		7
Effect of lanreotide on polycystic liver and kidneys in autosomal dominant polycystic kidney disease: an observational trial.<br><br>PMID: 25369108	Gevers, T.J.	Liver Int. 35(5):1607–14.	2015	Open label clinical trial<br><br>(RESOLVE trial)	43 ADPKD; 18 - 70 years of age; eGFR > 30 ml/min/1.73 m^2. 6 months of lanreotide (120mg)	Change in TKV (MRI)<br><br>Change in eGFR (MDRD and CKD-EPI formula)	Change in TKV after 6 months: (p=0.006)<br>LAR (n=43): 1023(619-2365) → 1012(597-2378) ml<br>-1.7 ±3.4%<br>Change in eGFR after 6 months: (p=0.006)<br>LAR (n=43): 63 ±17 → 60 ±17 ml/min/1.73 m^2<br>Main GFR decrease in the first 4 weeks; thereafter stable GFR.<br>Initial decline in eGFR was associated with a small, although not significant, increase in mean arterial pressure (MAP).	Not serious	Not serious	Not serious	Not serious	Single-arm, no placebo control<br><br>Short duration	6
The use of lanreotide in polycystic kidney disease: a single center experience.<br><br>PMID: 24707279	Treille, S.	Case Rep Nephrol Urol 4:18-24	2014	Case reports	6 ADPKD patients; > 18 yrs of age	Change in TKV (CT) after 6 months(n=2), 12 months (n=2) or 18 months (n=2)	Mean decrease in TKV 4%<br><br>18 months (n=2): -16% and -3.7%<br>12 months (n=2): + 1% and -0.2%<br>6 months (n=2): -5.4% and -0.1%	Not serious	Not serious	serious	Not serious	Very small case series	4

Abbreviations: ADPKD = autosomal dominant polycystic kidney disease; CV= Cyst volume; TKV= total kidney volume; Tx= transplantation

Table S10b somatostatin analogues for delaying progression of liver disease in ADPKD

Title	First Author	Journal	Year	Study design	N^o & type of patients	Outcome measure(s)	Results	Risk of bias	Indirectness	Inconsistency	Imprecision	Other con-siderations	Importance (1-9)
**Randomized trials**													
Randomized clinical trial of long-acting somatostatin for autosomal dominant polycystic kidney and liver disease<br><br>PMID: 20431041	Hogan, M.C.	J Am Soc Nephrol 21(6): 1052–1061	2010	Randomized, double-blind, placebo-controlled trial (2:1)<br><br>(MAYO trial)	42 patients (34 ADPKD/ 8 ADPLD) ≥18 years of age; >4000 mL hepatic volume) receiving Octreotide-LAR or placebo for 1 year	Change in total liver volume (TLV) (MRI)	Change in TLV after 12 months: (p=0.048)<br>LAR (n=28): -4.95 ±6.77%<br>Placebo (n=14): +0.92 ±8.33%<br><br>Change quality of life over 12 months: (p=0.045)<br>LAR (n=28): +0.25 ±7.53%<br>Placebo (n=14): +8.61 ±10.07%<br>Patients on LAR reported an improved perception of bodily pain and physical activity	Not serious	Not serious	Not serious	Not serious	No serious side effects.	9
Long-term Effects of Octreotide on Liver Volume in Patients With Polycystic Kidney and Liver Disease. (ALADIN)<br><br>PMID: 26844873	Pisani, A.	Clin Gastroenterol Hepatol.14 (7):1022-1030	2016	Randomized, placebo-controlled, single center study	27 ADPKD patients (> 18 yrs; eGFR > 40 ml/min/1,73m^2) receiving LAR octreotide (n=14) or placebo (n=13)	Total liver volume after 3 years of treatment and 2 years after end of treatment	Change in TLV at 3 years: (p=0.003)<br>LAR (n=14): -130.2 ±133.2 ml<br>Placebo (n=13): +144.3±316.8 ml<br><br>Change in TLV at 2 years after end of treatment: (p=0.046)<br>LAR (n=14): -14.4± 138.4 mL<br>Placebo (n=13): +224.4±331.7 ml<br>(i.e. reduction maintained for 2 years after treatment end)	Not serious	Not serious	Not serious	Not serious	No relevant side effects	8
Reducing polycystic liver volume in ADPKD: effects of somatostatin analogue octreotide.<br><br>PMID: 20185596	Caroli, A.	Clin J Am Soc Nephrol. 5(5):783-9	2010	Post hoc analysis of prospective, randomized, double-blind, crossover study (Ruggenenti, P. et al)	12 ADPKD patients > 18 yrs (median age 44.5 years); eGFR > 40 ml/min/1.73m^2	Change in TLV (CT)	TLV after 6 months: (p<0.005)<br>LAR: 1595 ± 478 ml → 1524 ± 453 ml<br>Placebo: 1580 ± 487 → 1594 ± 480 ml<br>Liver cyst volume after 6 months: (p=ns)<br>LAR: 89 ± 146 →93 ± 156<br>Placebo: 89 ± 148 → 89 ± 146<br>Liver parenchymal volume after 6 months: (p<0.005)<br>LAR: 1506 ± 431 → 1432 ± 403<br>Placebo: 1490 ± 439 → 1504 ± 433<br><br>Changes in liver volumes were significantly correlated with concomitant changes in kidney volumes (r = 0.67) during octreotide but not during placebo treatment.	Not serious, small study	Not serious	Not serious	Not serious	No serious side effects;<br><br>Short duration of treatment	8
Lanreotide reduces the volume of polycystic liver: a randomized, double-blind, placebo-controlled trial<br><br>PMID: 19646443	Keimpema, L.	Gastroenterology. 137(5):1661–8 e1–2	2009	Randomized, double-blind, placebo-controlled trial<br><br>(LOCKCYST Trial)	54 patients (n=32 ADPKD and n=22 ADPLD, aged >18 years; >20 liver cysts) receiving either lanreotide or placebo for 6 months	Total liver volume (TLV) (CT) at 0 and 24 weeks	Change in TLV after 6 months: (p<0.01)<br>LAR (n=27): 4606 (547-8665) → 4471(542-8401) ml, -2.9%<br>Placebo (n=27): 4689 (613-8765) → 4896 (739-9035) ml, +1.6%	Not serious	Not serious	Not serious	Not serious	No serious side effects;<br><br>Short duration of treatment	8

Title	First Author	Journal	Year	Study design	Nº & type of patients	Outcome measure(s)	Results	Risk of bias	Indirectness	Inconsistency	Imprecision	Other considerations	Importance (1-9)
**Non-randomized studies**													
Somatostatin analog therapy for severe polycystic liver disease: results after 2 years<br><br>PMID: 22773240	Hogan, M.C.	Nephrol Dial Transplant 27:3532-3539	2012	Open label extension of MAYO trial (Hogan 2010)	41 patients (34 ADPKD/ 8 ADPLD), aged ≥18 years; >4000 mL hepatic volume)<br>(O-O): 1st and 2nd year octreotide<br>(P-O): 1st year placebo, 2nd year octreotide	Change in TLV (MRI)	Change in TLV in 2nd year:<br>O → O (n=27): -5.23 ±6.22% (1st year) (p=0.0002)<br>-0.77±6.82% (2nd year) (p=0.57)<br>P → O (n=14): +0.9±8.35% (1st year)<br>-6.08±7.58% (2nd year) (p=0.001)	Not serious	Not serious	Not serious	Not serious	One hepatic cyst infection associated with octreotide	8
Alkaline phosphatase predicts response in polycystic liver disease during somatostatin analogue therapy: a pooled analysis<br><br>PMID: 26481454	Gevers, T.J.	Liver Int. 36(4):595-602.	2016	Pooled analysis of 4 clinical trials (LOCKCYST (n=51), ELATE (n=32); RESOLVE (n=28), Mayo (n=42))	153 patients with polycystic liver disease (100 ADPKD)	Prediction of liver volume reduction	Elevated baseline AP was associated with increased liver volume reduction during therapy with somatostatin analoga: Overall (n=153): -2.7% (-5.1 to -0.2%) mean reduction in TLV<br><br>ADPKD subgroup (n=100): elevated alkaline phosphatase predicted liver volume reduction (−3.2%, P = 0.03) but did not predict kidney volume reduction (+0.1%, P = 0.97).	Not serious	Not serious	Not serious	Not serious	Post-hoc analysis. Overlap with previous papers.	7
Efficacy of 4 years of octreotide long-acting release therapy in patients with severe polycystic liver disease.<br><br>PMID: 26166166	Hogan, M.C.	Mayo Clin Proc.; 90(8):1030-7.	2015	Open label observational extension of Hogan MC. 2012	21 ADPKD patients formerly enrolled in a (1 year RCT (Hogan 2010 (13 initially on LAR, 8 initially on placebo) and 2 year open-label-extension (all on LAR). Re-enrolment on another 2 years of LAR-octreotide after being mean pause of 8.3 months	Change in TLV (MRI)	Change in TLV off LAR: (p=0.11)<br>LAR (n=21): 5304 →5368 ml +3.4±8.2%<br><br>Change in TLV on LAR in 2 year extension : (p=0.02)<br>LAR (n=21): 5368 →5138ml -4.7 ±6.1%<br><br>Change in TLV after 4 years (baseline vs.4 years) : (p=0.06)<br>LAR (n=21): 5863 →5138ml -11.75 %	Serious	Not serious	Not serious	Not serious		7
Lanreotide reduces liver volume, but might not improve muscle wasting or weight loss, in patients with symptomatic polycystic liver disease.<br><br>PMID: 26073493	Temmerman, F.	Clin Gastroenterol Hepatol; 13(13): 2353-9	2015	Oberservational trial.<br><br>Lanreotide for 18 months: 90 mg for 6 months, then dose adjustment	51 ADPKD, 8 ADPLD; > 18 years of age<br>Responder: 90 mg for another 12 months<br>Non-responder: 120 mg for another 12 months	Change in TLV<br><br>Responder = TLV reduction > 100 ml, non-responder: TLV reduction < 100 ml	Responder (n=21): mean TLV reduction -430 ± 92 ml after 18 months<br><br>Non-responder (n=32): mean TVL increase 120 ± 42 ml at 6 months ; no further increase after 18 months	Not serious	Not serious	Not serious	Not serious	Single-arm, no placebo control	7

Title	First Author	Journal	Year	Study design	Nº & type of patients	Outcome measure(s)	Results	Risk of bias	Indirectness	Inconsistency	Imprecision	Other con-siderations	Importance (1-9)
The long-term outcome of patients with polycystic liver disease treated with lanreotide<br><br>PMID: 22111942	Chrispijn, M.	Aliment Pharmacol Ther 35:266-274	2012	Open-label observational extension trial of the LOCKCYST trial	41 patients (ADPKD n=25; ADPLD n=16), aged >18 years; >20 liver cysts; on lanreotide for 12 months in total	TLV (CT) at 0 and 12 months	Change in TLV after 12 months: (p<0.05)<br>LAR (n=41): 4974 (2982-6299) → 4711(2977-6355) ml<br>-4.0%	Not serious	Not serious	Not serious	Not serious		7
Effect of lanreotide on polycystic liver and kidneys in autosomal dominant polycystic kidney disease: an observational trial.<br><br>PMID: 25369108	Gevers, T.J.	Liver Int. 35(5):1607–14.	2015	Open label clinical trial<br><br>(RESOLVE trial)	43 ADPKD; 18 - 70 years of age; eGFR > 30 ml/min/1.73 m^2. 6 months of lanreotide (120mg)	Change in TLV (MRI)	Change in TLV after 6 months: (p<0.001)<br>LAR (n=43): 4859 (3110-7822) → 4595 (3172-7910) ml<br>-3.1 ±4.6%	Not serious	Not serious	Not serious	Not serious	Single-arm, no placebo control<br><br>Short duration	6

Abbreviations: ADPKD = autosomal dominant polycystic kidney disease, CV= Cyst volume,TKV= total kidney volume; Tx= transplantation

Table S12 Patient perspectives and quality of life in patients with ADPKD

Title	First Author	Journal, Year	Study design	Nº & type of patients	Main results	Issues relevant to children & parents & bias
**Patient perspectives on ADPKD**						
**Patient-focused outcomes for guidelines and research**						
Identifying and integrating consumer perspectives in clinical practice guidelines on autosomal-dominant polycystic kidney disease.<br><br>PMID: 26235729	Tong, A	Nephrology (Carlton). 2016 21(2):122-32	Workshop with 3 focus groups for developing patient-centered outcomes (and comparison to physican's priorities.	15 patients with ADPKD and 3 caregivers.	22 priority topics for guidelines were identified. Patients' concerns focused around:<br>- non-pharmacological management (diet, fluid intake, physical activity, complementary medicine)<br>- pain management and psychosocial care (mental health, counselling, cognitive and behavioural training, education, support groups).<br><br>26 patient focused outcomes including: quality of life (QoL), progression of kidney disease, kidney function, cyst growth and nephrotoxity.<br>Six themes reflected reasons for their choices: clarifying ambiguities, resolving debilitating pain, concern for family, preparedness for the future, taking control and significance of impact.<br>Almost all topics and outcomes suggested were identified by health professionals with the exception of five topics/outcomes.	13/18 participants had children themselves, and 10/15 affected adults had positive family history but only 3/15 diagnosed in childhood themselves.
A painful inheritance-patient perspectives on living with polycystic kidney disease: thematic synthesis of qualitative research.	Tong, A	Nephrol Dial Transplant. 2015 30(5):790-800.	Meta-analysis of qualitative studies exploring the perspectives of adults with ADPKD.	21 studies with 247 ADPKD patients with all stages of CKD and post-TPL	5 overarching themes:<br>unvalidated pain (medical trivialization, inadequacy of pain management);<br>persisting uncertainties and ambiguities (lacking diagnostic clarity, disempowerment in self-care, unpredictable daily disruptions, inability to plan ahead, financial discrimination);<br>genetic guilt and resentment (blaming parents, selfblame, constant burden of guilt);<br>precariousness in pursuing parenthood (prognostic uncertainty, owning the decision, needing directive counselling);<br>defining parental responsibility for genetic testing and disclosure (preserving normality, doubting necessity of genetic testing, respecting the child's autonomy and hope in future technologies, facilitating preparedness).<br><br>Summary: The erratic onset of pain contributes to the substantial unpredictability of daily living and prevents patients from establishing long-term life goals. Decisions about family planning, genetic testing of children and disclosure involves making profoundly difficult judgments about ethical parental responsibility.<br><br>Patient engagement in pain management, strategies for self-care, counselling to reduce the burden of genetic guilt' and specific family planning decision support tools may be priorities for care to improve patient-centered outcomes in ADPKD.	Genetic guilt and resentment, as well as responsibility for genetic testing and disclosure (i.e. 2 of 5 issues) are key issues which are likely to impact children and parent-child relationships.<br><br>Uncertainties about the future and parenthood as well as difficulty in establishing long-term life goals are likely to affect young adults.<br><br>Pain is already a relevant finding in childhood (see literature review on Complications in childhood ADPKD, prevalence 16-31%).
Patient-reported outcomes in clinical trials of CKD-related therapies: report of a symposium sponsored by the national kidney foundation and the U.S. Food and Drug Administration.<br><br>PMID: 23988757	Perrone, RD	Am J Kidney Dis. 2013 62(6):1046-57.	Report from a symposium on patient-reported outcomes in clinical trials, with a special section on ADPKD.	Not clear if/how many ADPKD patients attended.	All health-related quality of life measures should be regarded as patient-reported outcomes, but should be adapted to the disease in question.<br><br>Suggest dividing the symptoms of ADPKD into 2 categories:<br>- Direct symptoms (of kidney growth or dysfunction): eg, pain, abdominal fullness (satiety, body image, nausea, constipation), polyuria, nocturia, dysuria, hematuria, dyspnea<br>- Indirect symptoms (of associated abnormalities): eg. Headache from hypertension, insomnia, depression<br><br>Provides 2 end point models for clinical trials that incorporate patient-reported outcomes.	

Title	First Author	Journal, Year	Study design	Nº & type of patients	Main results	Issues relevant to children & parents & bias
**Patient and Clinician's attitudes towards (very) early diagnosis**						
Attitudes of at-risk and affected individuals regarding presymptomatic testing for autosomal dominant polycystic kidney disease.<br><br>PMID: 2333880	Sujansky, E	Am J Med Genet. 1990 35(4):510-5.	Cross-sectional study.<br><br>Questionnaire on knowledge about ADPKD, its inheritance, own family planning & willingness to utilize gene testing for oneself or offspring.<br>Age group 18-40 years only: willingness to terminate pregnancy	141 ADPKD affected and 137 unaffected but at-risk individuals. Consecutive recruiting from a specialist clinic.<br><br>ADPKD defined by self-reporting.<br><br>No disease markers.<br><br>Mean age of affected 42.2 ± 1 years. Study included 3 patients < 18 years, and 59 (21%) 18-30 years old.	Both groups of individuals displayed a high level of knowledge about the disease.<br>66% of ADPKD and 74% of at-risk individuals were worried about passing on the gene to their children (NS).<br><br>87% of affected individuals considered the recurrence risk of ADPKD high, but only 11% of ADPKD did not have children for this reason after diagnosis.<br>18% of ADPKD and 10% of at-risk individuals would not have had children if they had known they were at-risk for ADPKD (p=ns).<br><br>97% of at-risk individuals would utilize gene testing for themselves (no influence of gender, age, ethnicity, or disease severity in the family).<br>88% of ADPKD and 89% of at-risk individuals would test offspring (not significant, NS)<br><br><u>Individuals aged 18 to 40 years:</u><br>18% of ADPKD and 8% of at-risk individuals will not have more children because of the genetic risk (NS).<br>65% of ADPKD and 50% of at-risk individuals would use prenatal testing (NS).<br>4% of ADPKD and 8% of at-risk individuals would terminate a pregnancy for ADPKD.<br>A greater percentage would terminate a pregnancy for a serious medical problem.	High worry about passing on the disease, even in at-risk individuals.<br>Acceptance of genetic testing: v. high for onself, high for offspring and medium for pregnancy.<br><br>Risk of bias:<br>Virtually no young people included.<br>Old study (conducted 1985-1988), which may not reflect current patient attitudes.<br>At-risk individuals had a greater percentage of Catholics, lower level of education and lower level of income compared to affected (all p<0.05).
Attitudes in Patients with Autosomal Dominant Polycystic Kidney Disease Toward Prenatal Diagnosis and Preimplantation Genetic Diagnosis<br><br>PMID: 28961265	Swift, O.	Genet Test Mol Biomarkers. 2016 Dec;20(12):741-746	Cross-sectional study.<br><br>Questionnaire on attitudes towards preimplantation genetic diagnosis	96 ADPKD patients (38 with ESRD and 58 with CKD stage 1-4).<br><br>Mean age 51.5 years (only 9 < 30 years, all with CKD).	Would consider prenatal diagnosis and termination of pregnancy:<br>17 % of ADPKD patients with CKD and 18% of ADPKD patients with ESRD<br><br>Would have opted for PGD (or might consider it in the future) were it available and funded by the UK NHS:<br>50% of ADPKD patients with CKD and 63% in the ESRD group (p = 0.33).<br><br>Believe that PGD should be offered to other patients:<br>69% of ADPKD patients with CKD and 68% in the ESRD group<br><br>There was a spectrum of attitudes among this cohort.	
Clinicians' attitude towards family planning and timing of diagnosis in autosomal dominant polycystic kidney disease<br><br>PMID: 28961265	De Rechter, S.	PLoS One. 2017 Sep 29;12(9):e0185779.	Cross-sectional study.<br><br>Online questionnaire to different medical specialists	410 clinicians (53% male, mean (SD) age of 48 (10) years).<br><br>216 pediatric nephrologists, 151 adult nephrologists, and 43 clinical geneticists.	While the 3 groups agreed to encourage clinical testing in asymptomatic ADPKD minors and adults, only geneticists would recommend genetic testing in asymptomatic at-risk adults (P<0.001).<br><br>Statistically significant disagreement between disciplines was observed regarding the ethical justification of prenatal genetic diagnosis, termination of pregnancy and pre-implantation genetic diagnosis (PGD) for ADPKD. Particularly, PGD is ethically justified according to geneticists (4.48 (1.63)), whereas pediatric (3.08 (1.78); P<0.001) and adult nephrologists (3.66 (1.88); P<0.05) appeared to be less convinced.	Most clinicians supported clinical testing of at-risk minors, which is partially ad odds with exisiting recommendations (which recommend not to test asymptomatic minors).

Title	First Author	Journal, Year	Study design	Nº & type of patients	Main results	Issues relevant to children & parents & bias
**Quality of life in adults with ADPKD**						
The effect of disease severity markers on quality of life in autosomal dominant polycystic kidney disease: a systematic review, meta-analysis and meta-regression.<br><br>PMID: 28545401	Neijenhuis, MK	BMC Nephrol. 2017 18(1):169	Metaanalysis of trials assessing quality of life with SF36 in patients with ADPKD.<br><br>Normal population score: 50 ± 10.	9 studies including 1594 non-dialysis ADPKD patients with GFR values available.<br>7 studies reported kidney volume (n=1238), 5 studies reported liver volume (n=1057).<br>Mean age 44 years, 45% male.<br>Pooled mean eGFR 58, mean kidney volume 1,465ml (~4x normal), mean liver volume 3,599 ml (~2.5x normal).	Physical component summary score:<br>45.7 (95% CI 42.7 to 48.7) = different from reference population (p<0.001), but with significant heterogeneity btwn studies. Comparison to age-corrected reference values also highly significant (p<0.001).<br>Impact of markers of severity of disease (meta-regression):<br>Liver volume had closest association to physical component score p<0.01, but no significant association to eGFR, kidney volume, or total liver+kidney volume. In studies with mild to moderate liver involvement only, liver volume and renal function were correlated, but not kidney volume.<br><br>Mental component summary score:<br>47.8 (95% CI 45.7 to 49.8) = different from reference population (p<0.05), but with significant heterogeneity btwn studies. Comparison to age-corrected reference values highly significant (p<0.001).<br>Impact of markers of severity of disease (meta-regression):<br>Liver and total liver+kidney volume were significantly association to mentall component score p<0.01, with no significant association to eGFR or kidney volume (same in studies with mild to moderate liver involvement only).	ADPKD adults have lower physical and mental quality of life scores, and physical domain is affected more.<br><br>Liver volume has the largest impact on QOL. In patients with mild to moderate liver involvement, GFR has a closer relationship to physical QOL than kidney volume.<br><br>Bias:<br>Adult studies only. Hepatic cysts are rare (< 5%) in children.<br>Comorbidities, pain, presence of cerebral aneurysms, education level and genetic guilt are not accounted for. Many studies from controlled trials leads to pre-selected patients.
Increased psychosocial risk, depression and reduced quality of life living with autosomal dominant polycystic kidney disease.<br><br>PMID: 26268712	Simms, RJ	Nephrol Dial Transplant. 2016 31(7):1130-40.	Cross sectional, single center study.<br><br>Questionnaires:<br>1. QOL: general (SF36) and kidney disease quality-of-life short form (KDQOL-SF1.3)<br><br>2. Depression: 9 item patient health questionnaire (PHQ-9)<br><br>3. Social support: Multidimensional Scale of Perceived Social Support (MSPSS)<br><br>4. ADPKD-Tool: modified genetic psychosocial risk questionnaire (GPRI-ADPKD).	Patients > 18 years with ADPKD, but without ESRD.<br><br>53% (158/299) responded to invitation, 19 not analyzed → n=139<br><br>Mean age 8.9 ± 16.0 years, 51.8% female.<br>eGFR 7–141 mL/min, (analyzed in 3 groups of <30, 30-60 and > 60ml/min)<br><br>Mean kidney length (9.6–26.0 cm) (analyzed in 2 groups >/< 17 cm).<br><br>83.6% were under follow-up.<br><br>Non-respondents were younger, but ♀=♂ and equally often under follow-up.	Quality of life, general:<br>- Patients in the lower eGFR group scored significantly lower for physical functioning, role physical, general health, sexual function, vitality, but not for bodily pain, emotional well-being and role emotional.<br>- Patients with larger kidneys scored significantly lower for physical functioning, role physical and general health, but not for other items.<br>- Multiple regression for physical component summary score associated independently with eGFR, education, smoking and comorbidity (accounting 40% variance)<br>Quality of life, kidney disease-specific health concerns<br>- Patients in the lower eGFR group scored significantly lower for effects of kidney disease, burden of kidney disease, work status and sexual function, but not symptoms, cognitive function, quality of social interaction, sleep and social support.<br>- Patients with larger kidneys scored significantly lower for effects of kidney size only.<br>- Multiple regression for burden of kidney disease: liver cysts and having a known intracranial aneurysm (ICA) were independent predictors.<br>- Multiple regression for effects of kidney disease: eGFR, gender and known ICA.<br>Depression<br>- Clinically significant depression was reported in 22%.<br>- Multiple regression analysis for depression reveled younger age, physical component summary score in QOL questionnaire and pain as independent predictors<br>Social support<br>- Patients with lower eGFR reported significantly lower scores.<br>- Non-significant trend for kidney-size groups.<br>Genetic psychosocial risk instrument –ADPKD (GPRI-ADPKD)<br>- 74% reported more problems in their life because of ADPKD, 72% concerned about progression to ESRD, 62% felt guilty about passing ADPKD on to their children, 37% had lost a first degree relative to ADPKD.<br>- Multiple regression for GPRI-ADPKD score: loss of 1st° relative, kidney size and depression were independent predictors.<br>Multiple logistic regression for all of the above with age, gender, eGFR and kidney length:<br>- Female gender is independent predictor for physical component summary of QOL score, mental component summary of QOL score, depression score and GPRI-ADPKD	Large proportion who feels guilty about passing ADPKD on to their children is likely to affect family relationships and children.<br><br>Younger age was significantly and independently associated with depression risk.<br><br>Non-responders were younger, which may suggest better psychological well-being. However, some did not wish to think about the psychosocial consequences (verbal communication).<br><br>Quality of life associated more with eGFR than with kidney size.<br>Change in QOL scores across eGFR groups similar to that of patients with CKD of any cause.<br><br>Bias:<br>Adult-only study.<br>Women were uniformly more vulnerable to each of the patient-reported outcomes, which may reflect a gender bias in willingness to admit symptomatology. (or the fact that liver size is most important for QOL, and women are more affected by polycystic liver disease?)

Title	First Author	Journal, Year	Study design	Nº & type of patients	Main results	Issues relevant to children & parents & bias
Health-related quality of life in patients with autosomal dominant polycystic kidney disease and CKD stages 1-4: a cross-sectional study.<br><br>PMID: 24183837	Miskulin, DC	Am J Kidney Dis.<br>2014<br>63(2):214-26	Cross-sectional, multicenter study.<br><br>Quality of life questionnaire (SF36).<br><br>Modified Wisconsin brief pain survey.	99% HALT-PKD study A and B participants → n=1031 for SF36 and n=1035 for pain scale.<br><br>3 GFR groups (22-44, 45-60, > 60 ml/min).<br><br>Mean age GFR > 60: 37 ± 9, GFR 45-60 47 ± 8, GFR 20-44: 49 ± 8 years.<br><br>TKV by MRI only routinely measured in patients with GFR > 60)	Pain:<br>- 51% had back pain in the last 3 months (30% sometimes, 21% often, usually or always).<br>Back pain frequency did not vary by eGFR subgroup in men or women.<br>- 28% had abdominal pain in the last 3 months (16% sometimes, 12% often, usually or always).<br>Abdominal pain frequency increased by eGFR subgroups in men (p=0.05), but not in women.<br>- Pain interfering moderately to extremely for the below increased with increasing eGFR:<br>Men: work (p=0.04), strenuous physical activity (p=0.02), social activities (p=0.02)<br>Women: walking ability (p=0.03)<br>Abdominal fullness:<br>- 17% had abdominal fullness interfering with usual activities (11% sometimes, 6% often, usually or always).<br>No association with GFR group<br>- 22.5% ate less due to abdominal fullness (13.5% sometimes, 9% often, usually or always)<br>Associated with GFR group in women (p=0.05)<br>- 11% had poor appetite because of nausea (9% sometimes, 3% often, usually or always)<br>Quality of live:<br>- In all 3 GFR groups patients scored the same as or higher than the general age-matched population except general Health, where they scored significantly less.<br>Relationship to htTKV and htTLV (n=609, GFR > 60 ml/min group only):<br>No correlation of GFR and pain, except in subgroup of TKV > 1L/m.<br>No correlation of TLV to pain. No correlation of htTKV to QOL.	Findings relate mainly to more advanced disease.<br><br>In group with GFR > 60 kidney size does not correlate to pain.<br>The absence of an effect on high reported frequency of pain on HRQoL suggests that either in most patients, these symptoms are mild or that patients have a tremendous resilience to adapt to their physical discomfort. This may be because they are comparing themselves to their family members with ADPKD who are on dialysis therapy or have died and thus view their current life circumstances favorably relative to them.<br>Bias:<br>Adults only, participants of randomized clinical trial represent a highly pre-selected group, both by willingness to take part in study and trial exclusion criteria<br>Familial and social factors not included.
Quality of life of patients with ADPKD-Toranomon PKD QOL study: cross-sectional study.<br><br>PMID: 23978051	Suwabe, T	BMC Nephrol.<br>2013<br>14:179-	Cross-sectional, single center data from a prospective observational study on QOL.<br><br>1. Quality of life questionnaire (SF36)<br>2. Own 12-item QOL questionnaire	N= 219 Japanese ADPKD patients (58% female).<br><br>Mean age 55.1 ± 11 years (all > 20 years)<br><br>N= 111 with CKD (mean creatinine 2.08 ± 1.9 mg/dl)<br>N=108 on dialysis (all HD, mean 75 ± 62 months).<br><br>Current laboratory values.<br>Abdominal MRI or CT in n=173	Age group of 30s only (n=17):<br>Physical and mental component summary score: not different to Japanese population in all ADPKD, CKD-only andHD-only groups<br>Role/social component summary score: not different to Japanese population in all & CKD-only groups, and lower in HD group (but n=2)<br><br>All age groups combined:<br>Physical component summary score: sig. lower than in Japanese population (p<0.001) and significantly lower in HD than in CKD patients.<br>Mental and role/social component summary scores: sig. lower than in Japanese population (p=0.005 and p<0.001)<br>Stepwise multiple regression for physical component summary score: Hb, serum albumin, ascites and cerebrovascular disease were sign. Independent predictors.<br>Stepwise multiple regression for mental component summary score: mental disease<br>Stepwise multiple regression for role/social component summary score: serum albumin.<br>No correlation of total liver and kidney volume with SF-36 scores.	Analysis by age<br><br>Bias:<br>Single center with expertise in transcatheter arterial embolization (TAE) for reduction of kidney and liver volumes in symptomatic patients. Thus probably biased population towards more pain and greater organ sizes (mean kidney volume in CKD patients much bigger than in Riwz. Et al study).<br><br>Familial and social factors not included in analysis.
Anxiety, depression, and quality of life in patients with familial glomerulonephritis or autosomal dominant polycystic kidney disease.<br><br>PMID: 21789424	de Barros, BP	J Bras Nefrol.<br>2011<br>33(2):120-8.	Cross-sectional, single center study.<br><br>Questionnaires:<br>1. State Trait Anxiety Inventory (STAI),<br>2. Beck Depression Inventory (BDI),<br>3. QOL-Short-Form SF-36,<br><br>& short interview	38 Brazilian ADPKD and 52 familial GN patients. All had ≥ 1 1st degree relative with CKD.<br><br>ADPKD group: mean age 38.5 ± 12 years, 76% female, 5 with ESRD, mean serum creatinine 1.1 mg/dl (range 0.7-1.9).<br>No demographic differences to familial GN group, except longer time since diagnosis.	Moderate anxiety was detected in both groups (worse than populations with diabetes and women with a family history of breast cancer in the UK).<br><br>Depression was found in 34.6% of familial GN and 60.5% of ADPKD patients (p=0.039). Both groups had a greater proportion of depressed patients than CKD, hemodialysis and CAPD groups from Turkey)<br><br>Anxiety and depression were more associated with female gender in familial GN, and with poorer schooling in ADPKD.<br>Patients of both groups presented two quality of life unfavorable dimensions: emotional role function and general health perception.<br><br>In addition, quality of life was worse among females, unmarried, and Caucasian subjects, and those individuals with a poorer educational level.<br><br>No correlation between serum creatinine or proteinuria with anxiety, depression or quality of life.	ADPKD appears to be more associated with depression than in familial GN.<br><br>Bias:<br>Adults only,

Title	First Author	Journal, Year	Study design	Nº & type of patients	Main results	Issues relevant to children & parents & bias
Quality of life in autosomal dominant polycystic kidney disease patients not yet on dialysis.<br><br>PMID: 19261830	Rizk, D	Clin J Am Soc Nephrol. 2009 4(3):560-6	Cross-sectional, multicenter study.<br><br>Quality of life questionnaire (SF36)	210 American ADPKD patients completed questionnaire, <18 years excluded, 16 not analyzable → n=152<br><br>Mean GFR 65 ± 33 ml/min*1.73 m^2<br><br>Groups: gender, pain-medication (yes/no), renal complications (yes/no), renal volume (</> 1L), hypertension (normal/high), renal function (eGFR </> 80ml/min) and education level (2 levels)	Physical and mental component summary score not different to general US population and African American study of Kidney Disease (eGFR 20-65 ml/min), but higher than in dialysis patients both of a clinical trial hemodialysis group and a community-based hemodialysis population.<br><br>Group taking pain medications scored significantly lower on physical function index, bodily pain index and physical component summary score.<br><br>Groups with college education scored higher on physical and mental component summary scores.<br><br>Group with GFR > 80 scored higher on physical component summary scores (PCS). And GFR correlated with physical function index (r=0.27, p=0.001), general health index score (r=0.29, p<0.001) and physical component summary score (r=0.29, p=0.0005).<br>Renal volume did not correlate to any of the scores.<br><br>Multiple regression analysis for physical component summary score: age, BMI, education level, pulse pressure and pain medication were independent predictors (32% variance explained).	Bias:<br>Adults only, participants of clinical trial represent a pre-selected group, both by willingness to take part in study and trial exclusion criteria (which included other systemic diseases, pregnant or lactating women, those with previous renal surgery).<br>Lack of difference between ADPKD and general CKD group suggest insensitivity of SF36.<br><br>Familial and social factors not included in analysis.
**Effect of interventions on quality of life**						
Effect of renal transcatheter arterial embolization on quality of life in patients with autosomal dominant polycystic kidney disease.<br><br>PMID: 28873973	Suwabe, T	Nephrol Dial Transplant. 2017 32(7):1176-1183	Prospective interventional study without control group.<br><br>1. Quality of life questionnaire (SF36)<br>2. Own 15-item QOL questionnaire	188 Japanese ADPKD ESRD patients before and up to 12 months after renal embolization procedure for symptomatic enlarged kidneys.<br><br>Mean age 56.7 ± 9 years.<br>Mean duration of dialysis 50 (18-101 months).	<u>Before → 1 year after renal transcatheter arterial embolization scores:</u><br>physical component summary (PCS) 38.21 (95% CI 36.50–39.91) → 42.0 (40.22–43.77; P<0.001)<br>mental component summary (MCS) 48.45 (47.05–49.86) → 51.25 (49.78–52.71; P=0.001)<br>role/social component summary (RCS) 43.04 (40.70–45.37) → 49.67 (47.22–52.12; P<0.001)<br><br>Scores for abdominal fullness, poor appetite and heartburn showed marked improvement after renal TAE, while scores for fever, bodily pain and sleep disorder also improved slightly, but significantly. Scores for constipation and use of analgesics/sleeping medications/laxatives did not improve significantly.<br><br>All of the SF-36 scores and the scores for specific symptoms (except bodily pain, snoring and constipation) were significantly correlated with the sequential decrease of the height-adjusted total kidney volume.	Risk bias:<br>Adults with ESRD and severe symptoms referred to tertiary center. Thus not a setting that is relevant for pediatrics.<br><br>Mental score increases before physical score (indicating a significant placebo effect?)
Laparoscopic cyst decortication in autosomal dominant polycystic kidney disease: impact on pain, hypertension, and renal function.<br><br>PMID: 12965058	Lee, DI	J Endourol. 2003 17(6):345-54	Observational study before and after laparoscopic cyst decortication procedure.<br><br>Pain relief (analog scale score).<br>Quality of life questionnaire (SF36)	29 ADPKD patients with chronic pain (n=19), hypertension (n=21), and renal insufficiency (n=10).<br><br>Mean age 45.5. years<br><br>Mean follow-up 32.3 months (range 6-72 months).	Relative pain reduction was 58% at 12 months, 47% at 24 months and 63% at 36 months.<br><br>Qualtiy of life:<br>Pain index, general health perception, vitality and mental health index increase up to 24months after treatment.<br>Physical functioning, social functioning and role emotional decrease at 1 month and increase to over-pre-treatment afterwards<br>Role physical decreases at 1 and 6 months and returns to pre-treatment levels afterwards.	Not very relevant for children.<br><br>Risk of bias:<br>No significances calculated for QoL scores.<br>Drop-out not accounted for in longitudinal analysis.
Somatostatin analogues improve health-related quality of life in polycystic liver disease: a pooled analysis of two randomised, placebo-controlled trials.<br><br>PMID: 26129925	Neijenhuis, MK	Aliment Pharmacol Ther. 2015 42(5):591-8	2 prospective, randomized, placebo-controlled trials of somato-statin analoge on polycystic liver disease.<br><br>Quality of life questionnaire (SF36)	87 Dutch and American patients with polycystic liver disease of which n=58 (67%) had ADPKD, with a mean eGFR of 63 ml/min.<br><br>Mean age 49 years,<br>89% female.	Baseline scores with and without ADPKD did not differ.<br><br>Physical component scores improved with somatostatin analogues, but remained unchanged with placebo (3.41 1.29 vs. 0.71 1.54, P = 0.044).<br>Treatment had no impact on the mental component score.<br>Large liver volume was independently associated with larger HRQL decline during follow up (-4-04 ± 2.02 points per logarithm liver volume, P = 0.049).<br><br>ADPKD patients with large liver and kidney volumes had larger decline in HRQL (5.36 ± 2.54 points per logarithm liver volume; P = 0.040 and -4.00 ± 1.88 per logarithm kidney volume; P = 0.039).	Risk bias:<br>Adult with severe disease<br>Includes:<br>Hogan et al, Somatostatin analog therapy for severe polycystic liver disease: results after 2 years. NDT 2012; 27(9):3532-9.<br>and<br>van Keimpema et al, Lanreotide reduces the volume of polycystic liver: a randomized, double-blind, placebo-controlled trial. Gastroenterology. 2009;137(5):1661-8.e1-2

Title	First Author	Journal, Year	Study design	Nº & type of patients	Main results	Issues relevant to children & parents & bias
**Quality of life scales developed for ADPKD patients**						
Development of the Autosomal Dominant Polycystic Kidney Disease Impact Scale: A New Health-Related Quality-of-Life Instrument. PMID: 29150246	Oberdhan, D	Am J Kidney Dis. 2017 Nov 14. pii: S0272-6386(17)30951-4.[Epub ahead of print]	Development of a patient-reported outcome questionnaire to assess ADPKD-related QoL	285 patients contributed in focus groups to generate questionnaire content. 15 patient debriefing interviews. 1374 patients as validation cohort (n=298 repeated after 1 month).	Scores on the physical, emotional, and fatigue domains of the ADPKD-IS differed significantly between patients in CKD stage 3b versus CKD stage 1 at baseline. Comparator questionnarire (SF12) only increased from CKD stage 3a onwards. Test-retest reliability coefficients (traditional correlations) for the 3 domains were high (0.89 for physical, 0.92 for fatigue, and 0.86 for emotional).	
Development and validation of a polycystic liver disease complaint-specific assessment (POLCA). PMID: 24996047	Temmerman, F.	J Hepatol. 2014 Nov;61(5):1143-50.	Development of symptom score for polycystic liver disease based on creation cohort and validation cohorts.	129 patients with polycystic liver disease (110 of which have ADPKD). Mean age 51 years. 111/129 (86%) female.	New score (POLCA) was validated in 2nd cohort before and after 6 months lanreotide. Score reduced more in group with greater liver volume change.	PCLD not relevant prior to puberty. After lanreotide only POLCA but no SF36 score given.
**Quality of life in children with CKD**						
No papers found						
